# Cell specific photoswitchable agonist for reversible control of endogenous dopamine receptors

**DOI:** 10.1038/s41467-021-25003-w

**Published:** 2021-08-06

**Authors:** Prashant Donthamsetti, Nils Winter, Adam Hoagland, Cherise Stanley, Meike Visel, Stephan Lammel, Dirk Trauner, Ehud Isacoff

**Affiliations:** 1 Molecular and Cell Biology, University of California, Berkeley, Berkeley, CA USA; 2 Department of Chemistry, Ludwig-Maximilians University, München, Germany; 3 Department of Chemistry, New York University, New York City, NY USA; 4 Helen Wills Neuroscience Institute, University of California, Berkeley, CA USA; 5 Molecular Biophysics & Integrated Bioimaging Division, Lawrence Berkeley National Laboratory, Berkeley, CA USA

**Keywords:** Biological techniques, Neural circuits, Neurophysiology

## Abstract

Dopamine controls diverse behaviors and their dysregulation contributes to many disorders. Our ability to understand and manipulate the function of dopamine is limited by the heterogenous nature of dopaminergic projections, the diversity of neurons that are regulated by dopamine, the varying distribution of the five dopamine receptors (DARs), and the complex dynamics of dopamine release. In order to improve our ability to specifically modulate distinct DARs, here we develop a photo-pharmacological strategy using a Membrane anchored Photoswitchable orthogonal remotely tethered agonist for the Dopamine receptor (MP-D). Our design selectively targets D1R/D5R receptor subtypes, most potently D1R (MP-D1_ago_), as shown in HEK293T cells. In vivo, we targeted dorsal striatal medium spiny neurons where the photo-activation of MP-D1_ago_ increased movement initiation, although further work is required to assess the effects of MP-D1_ago_ on neuronal function. Our method combines ligand and cell type-specificity with temporally precise and reversible activation of D1R to control specific aspects of movement. Our results provide a template for analyzing dopamine receptors.

## Abstract (web summary)

In this study, the authors develop a photo-pharmacological strategy using a Membrane anchored Photoswitchable orthogonal remotely tethered agonist for the Dopamine receptor (MP-D) to selectively and reversibly modulate the D1R receptor subtype.

## Introduction

Dopamine (DA) plays important roles in health (movement, reward, aversion, motivation) and disease (Parkinson’s, schizophrenia, addiction)^1,2^. To understand how DA action generates behavior, we need methods that can control specific aspects of DA function. DA neurons can be functionally interrogated by optogenetic stimulation of their cell bodies or axon terminals (Supplementary Fig. 1). However, DA neurons have diverse downstream targets, making it difficult to identify the casual basis of specific behavioral outcomes. For example, the striatum is a hub region that receives dense dopaminergic innervation from the midbrain^1^. Multiple distinct, spatially intermixed and inter-connected striatal cell types express various combinations of four of the five DA receptor (DAR) subtypes, the G_s/olf_-coupled D1-like receptors D1R and D5R and the G_i/o/z_-coupled D2-like receptors D2R and D3R (Supplementary Fig. 1e)^1^. Beyond the complex localization and distinct signaling properties of the different DARs, DA signals operate over phasic (hundreds of milliseconds) to slowly varying (tens of seconds to minutes) temporal domains^3^. Moreover, DA can be co-released with glutamate^4^ and/or GABA^5^, which may contribute to dynamic area-specific modulation^6^.

The relationship between DA release and the activation of specific striatal DARs in different cells is not well understood. In addition, there is evidence that local regulation of DA release in the striatum can occur independently of the action potential firing in DA neurons and is triggered or modulated by axo-axonal inputs onto the dopaminergic axons^7,8^. Indeed, different levels of DA input activity appear to selectively target D2R over D1R, according to distinct receptor affinities for DA^9^ but could also reflect targeted signaling.

Recent studies indicate that dopaminergic input from the substantia nigra compacta (SNc) to the dorsal striatum (dStr) initiates but does not sustain movement. SNc DA neurons become more active just before movement initiation^6,10^, and the brief optogenetic stimulation of these neurons increases the probability of movement initiation^11^. This raises the question: Which of the many targets of SNc DA neurons in the dStr (Supplementary Fig. 1e) triggers movement? A compelling candidate is D1R expressed in direct pathway medium spiny neurons (dMSNs) of the dStr. The activation of dMSNs has long been known to promote movement^12–14^. While some studies indicate that D1R can reduce dMSN excitability^15–17^, it is generally accepted that the activation of these receptors promotes dMSN excitability^18–22^. Moreover, a recent model suggests that D1R increases in activation in the dStr prior to movement initiation^10^, and the systemic activation of D1R with a synthetic agonist increases the probability of movement initiation^23^. For these reasons, D1R is a putative target for treatment of motor deficits in Parkinson’s disease^24^, where dopaminergic inputs from the SNc to the dStr degenerate. However, D1R agonists do not differentiate between D1R in dMSNs and the terminals of glutamatergic inputs in the dStr (Supplementary Figs. 1e and 2)^1^, and they also bind its close homolog D5R^25^, which is found in the four major classes of striatal interneurons (Supplementary Fig. 1e). Even if applied directly to the dStr, D1R agonists could diffuse and activate D1R and D5R in other brain areas with unpredictable kinetics. Alternatively, receptor knockout studies do not clearly define the role of D1R in movement (Supplementary Fig. 3)^26–29^ and suggest that D2R, which is also abundant in the dStr, can drive movement initiation^30^. Nevertheless, genetic modifications cannot be applied with temporal specificity and could result in system compensation, highlighting the need for a new method that can precisely control specific DARs.

Several approaches offer greater cell type and spatiotemporal specificity over GPCR signaling than conventional methods. Engineered DREADD and RASSL GPCRs enable orthogonal receptor activation through their use of an artificial ligand (Supplementary Fig. 2)^31^, but are non-native proteins that lack the localization and biochemical selectivity of endogenous receptors. Opto-XR chimeras of an opsin and the intracellular portions of a receptor of interest^32^ can be activated by light, but lack an endogenous ligand binding site and much of the native GPCR. DREADDs, RASSLs, and opto-XRs are more suited to overdrive signaling than substitute for the normal receptor. In contrast, t-toxins, DARTs, and Lumitoxins enable control of the endogenous receptor by tethering a ligand to a genetically-encoded plasma membrane protein anchor (Supplementary Fig. 2), but these are either persistently active^33^ or take minutes to hours to turn on or off^34,35^.

To examine D1R function in vivo, we implemented a photo-pharmacological strategy, along the lines that we developed recently for metabotropic glutamate receptors (mGluRs)^36^. In this strategy, a genetically-encoded membrane anchor (M) is selectively expressed in specific cells. The M captures a synthetic photoswitched ligand (P) by selective chemical conjugation to a protein tag, thereby restricting the P to a target class of cells in a specific location in the brain. Between the protein tag attachment and ligand ends of the P lies a flexible chain that is long enough to extend from the M anchor to a native receptor to which the ligand is suited. This approach follows the logic of DARTs^34,35^, with the key and unique feature that the P is photoswitchable^37–39^ and thus turns its target receptor on and off rapidly and reversibly in response to light, providing spatiotemporal control. We engineered the M to adapt the approach from the Family C mGluR, which has a large extracellular clamshell ligand binding domain “above” the transmembrane domain, to the Family A DAR, whose ligand binds within the transmembrane domain.

We developed a novel D1R/D5R selective-MP agonist that is most potent on D1R (hence called MP-D1_ago_). We targeted MP-D1_ago_ to dMSNs, where it would selectively activate D1R and avoid D5Rs in other striatal neurons (Supplemental Fig. 1e). Photo-activation of MP-D1_ago_ in the dMSNs of the dStr promoted movement initiation. Strikingly, this effect was as potent as optogenetic stimulation of dopaminergic nerve terminals that project to the dStr from the substantia nigra compacta (SNc). The MP method, which derives specificity from a combination of the P ligand and cell-specific expression of the M, should be applicable to other DARs and to neuronal receptors in general, providing insight into neural circuit function and neuromodulation.

## Results

### Design of a D1R MP

**Fig. 1:**
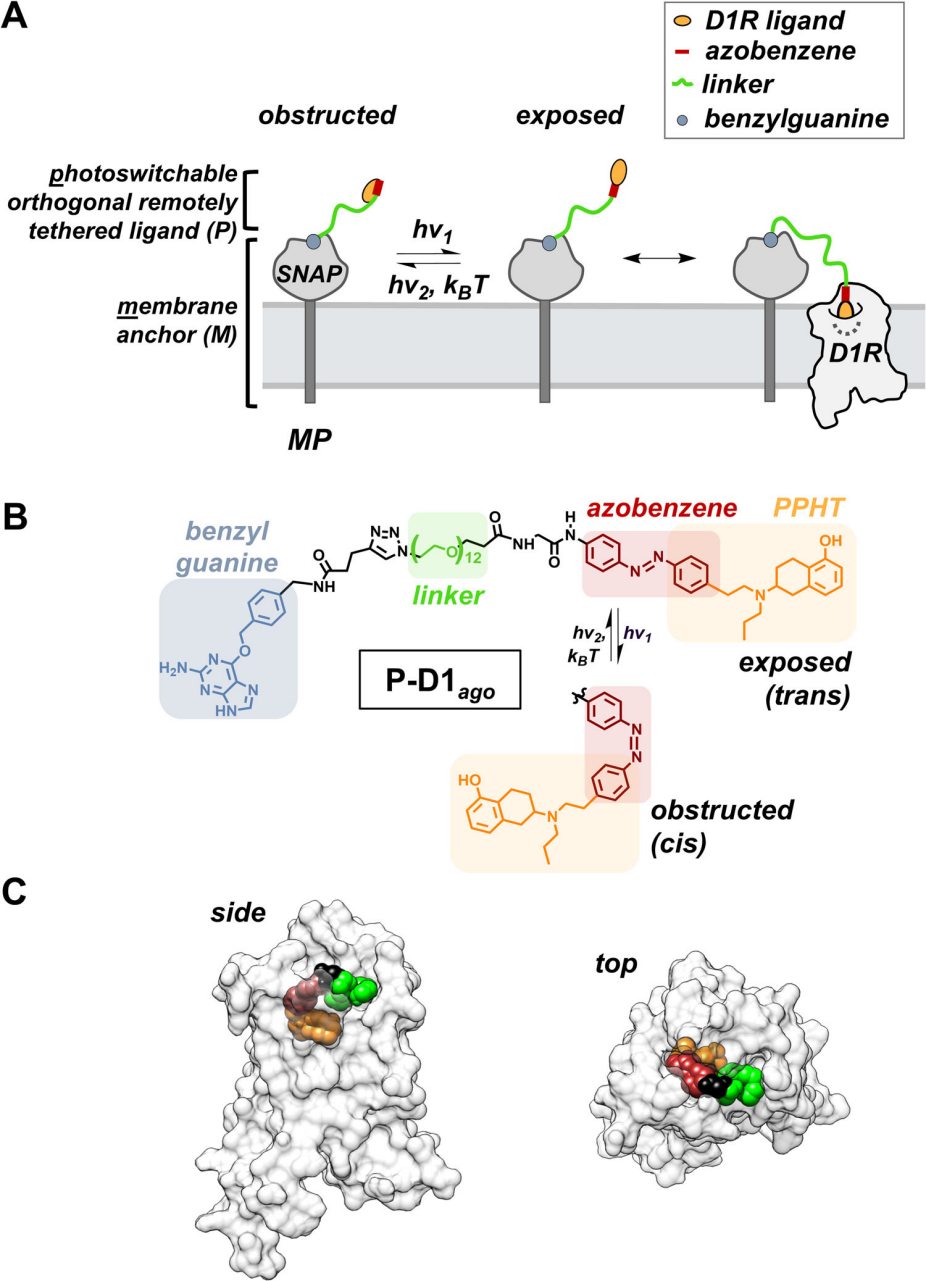
Design of a D1R MP. (**A**) Schematic representation of a D1R MP. The MP consists of D1R ligand that is tethered to the plasma membrane via membrane anchor (M) consisting of SNAP-tag and transmembrane segment. The MP rapidly switches between its *trans-* and *cis-* isomers in response to specific wavelengths of light (hν_1_ and hν_2_), or slowly from the *cis-* to *trans-*isomer in the dark via thermal relaxation (*k*_*b*_*T*). The MP is designed to bind D1R only in the photoisomeric state that the receptor ligand is exposed. (**B**) The chemical structure of the D1R photoswitch benzylguanine-azobenzene-PPHT, a photoswitchable D1R agonist (P-D1_ago_). Azobenzene (maroon) was integrated into the D1R agonist PPHT (orange) and is separated from benzylguanine (blue) via a 12-repeat polyethylene glycol (PEG) linker (green). (**C**) Model of a fragment of P-D1_ago_ (*trans-*PEG_4_-azobenzene-PPHT) docked in the dopamine (DA) binding site of D1R viewed from either the side (upper panel) or the top (bottom panel). Note that the linker (green) exits the pore that leads to the ligand binding site within the transmembrane domain of D1R.

MP is composed of two components, a photoswitchable orthogonal remotely tethered ligand (P) and a membrane anchor (M) that restricts the P to the cell surface (Fig. 1a). The P component contains a receptor ligand at one end, a protein-tag reactive moiety at the other end, an adjustable chemical linker in between, and an azobenzene that is flush to or merged with the receptor ligand. The azobenzene photoisomerizes between its *trans* and *cis* configurations in milliseconds^40^, rapidly switching the receptor ligand between two states: one that is exposed and can bind its receptor and one that is obstructed by the azobenzene and so cannot bind. The P is restricted via the reactive moiety to the membranes of cells that express the M, a genetically-encoded membrane-anchored protein-tag. The chemical linker between azobenzene and reactive moiety is long enough to allow the P to reach from its anchor point in the M to its binding site in the receptor.

To develop a P for D1R, we used a metabolically stable analog of DA, 2-(N-phenethyl-N-propyl)-amino-5-hydroxytetralin (PPHT; Supplementary Figure 4a), as a parent molecule. We showed previously that PPHT can tolerate the integration of azobenzene at its alkylbenzene^41^. When tethered directly to a cysteine introduced adjacent to the DA binding site of D1R via a maleimide group, azobenzene-PPHT binds the receptor in its *trans* state but not its *cis* state^41^. Analogously, we synthesized an azobenzene-PPHT analog containing the reactive moiety benzylguanine (photoswitchable D1 agonist or P-D1_ago_), which binds covalently and selectively to an M that is composed of SNAP-tag anchored to the plasma membrane via a single-pass transmembrane segment from low density lipoprotein receptor (Fig. 1b and Supplementary Fig. 4b). Molecular modeling was used to optimize the positioning (Supplementary Fig. 5a) and length (Supplementary Fig. 5b-e) of the polyethylene glycol (PEG) chemical linker between the azobenzene-PPHT and the benzylguanine attachment site.

### Optimization and characterization of the D1R MP

P-D1_ago_ switches from its *trans-*isomer to its *cis*-isomer under 370 nm (UV) light and back to *trans-*isomer under 460 nm (blue) light (Supplementary Fig. 6a, b). P-D1_*ago*_ binds efficiently to the M on the cell surface (Supplementary Fig. 6c–f) and is a low potency full agonist of D1R in its untethered form (Supplementary Fig. 7).

**Fig. 2:**
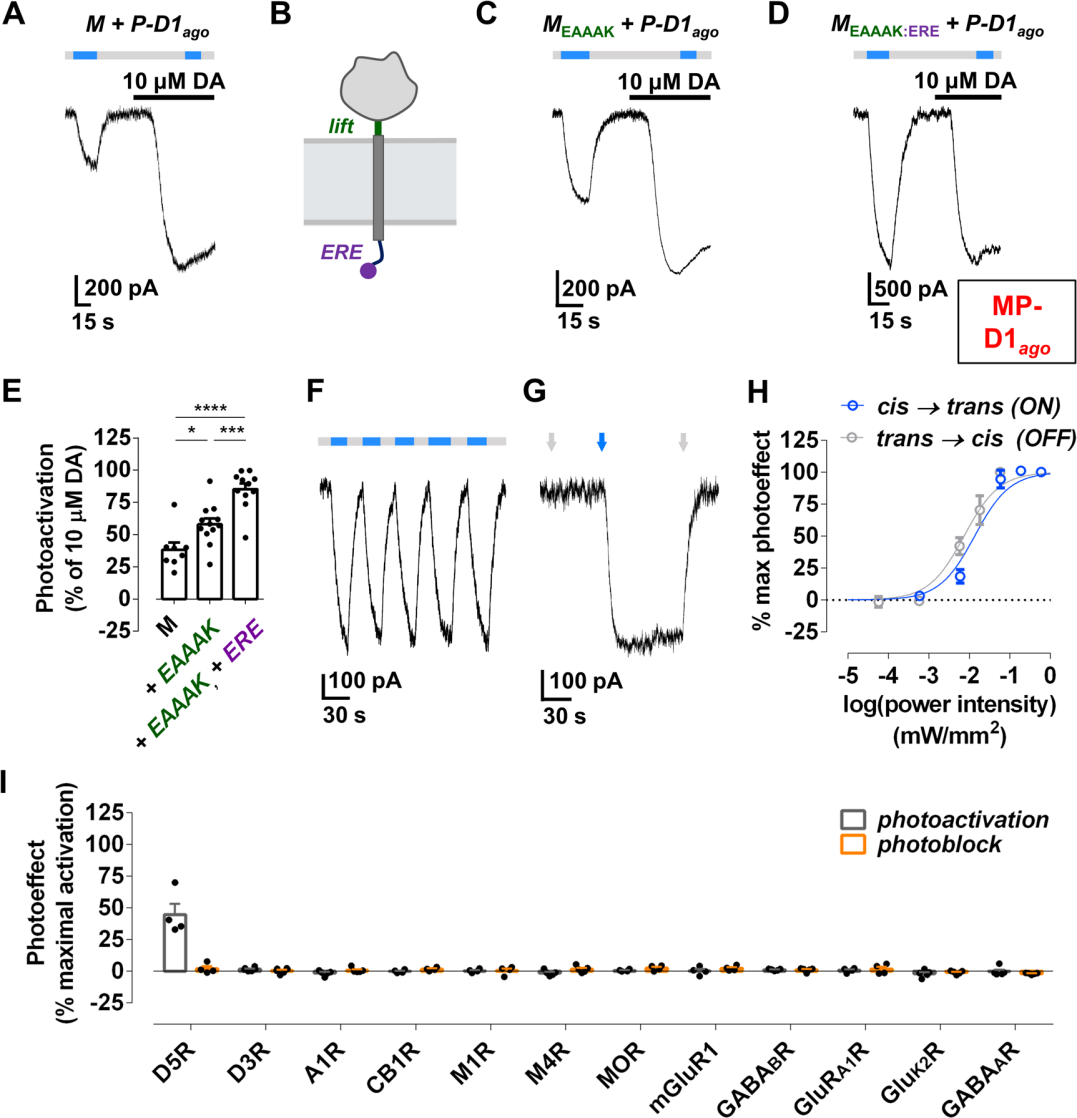
MP-D1_ago_ photoactivates D1R. (**A**) Photoactivation of D1R by P-D1_*ago*_ tethered to the M, according to a receptor mediated-GIRK activation assay in HEK293T cells. The receptor is partially photoactivated with blue light (460 nm) and is deactivated with UV light (370 nm) relative to a saturating concentration of DA. (**B**-**D**) The incorporation of the rigid lift peptide EAAAK (dark green) and an endoplasmic reticulum export motif (ERE; purple) in the M enhances photoactivation of D1R. P-D1_*ago*_ attached to M_EAAAK:ERE_ is MP-D1_ago_. (**E**) Summary of photoactivation of D1R with variants of the MP with different Ms. one-way ANOVA, F = 22.5, Tukey, *p < 0.05, ***p < 0.001, ****p < 0.0001. n = 8 cells for M, n = 12 cells for M plus lift, and n = 11 for M plus lift and ERE. (**F**) Photoactivation of D1R with MP-D1_ago_ is rapid, reversible, and repeatable. (**G**) Photoactivation of D1R with MP-D1_ago_ is bistable. Arrows indicate a one second flash of UV or blue light. (**H**) Photoactivation and deactivation of D1R with MP-D1_ago_ as a function of light intensity. n = 5 cells per condition. (**I**) Summary of photoactivation (black) or photoblock of agonist-activation (red) by MP-D1_ago_ of selected receptors coexpressed in HEK293T cells. MP-D1_ago_ photoactivation of D5R is significantly greater than that of all other receptors tested. one-way ANOVA, F = 27.5, Tukey, p < 0.0001. n = 4 cells for D5R, CB1R, M1R, MOR, and GluR_A1_R. n = 5 cells for A1R, M4R, GABA_B_R, Glu_K2_R, and GABA_A_R. n = 6 cells for D3R. See Supplementary Fig. 12 for experimental details. Error bars indicate S.E.M.

To test the ability of P-D1_ago_ to photo-activate D1R, we first attached it directly to a version of D1R that has SNAP fused to its extracellular N-terminus, which is adjacent to the ligand binding site of the receptor. SNAP-D1R was co-expressed in HEK293T cells with the G protein-coupled inwardly rectifying potassium (GIRK) channel as an effector. Following application and washout of unbound P-D1_ago_, blue light (*trans*) activated and UV light (*cis*) deactivated GIRK current (Supplementary Fig. 8), demonstrating P-D1_ago_ as an effective light-switched tethered ligand. With this in hand, we turned to attaching P-D1_ago_ to the M, which was co-expressed in HEK293T cells with the native D1R and the GIRK channel. In this inter-protein configuration, P-D1_ago_ once again photoactivated ion current by blue light and was deactivated by UV light (Fig. 2a, e), an effect that required expression of GIRK channels (Supplementary Fig. 9a) and D1R (Supplementary Fig. 9b, c), which indicated that inter-protein photo-activation of native D1R works. To optimize D1R photoactivation, we adjusted the geometric positioning of P-D1_*ago*_ using variants of M with “lift” peptides between the SNAP-tag and transmembrane segment. Photoactivation increased with an M variant containing a rigid, α-helical EAAAK peptide (M_EAAAK_ Fig. 2b, c, e) and decreased as the lift peptide was further extended (Supplementary Fig. 9d–f), presumably because P-D1_*ago*_ “rises” too far from the cell surface.

Pharmacological analyses suggested that when P-D1_ago_ is tethered to M_EAAAK_, it partially activates D1R because it is present at a submaximal concentration at the cell surface (Supplemental Fig. 10a–g). To increase surface P-D1_ago_, we added to the C-terminus of M_EAAAK_ an endoplasmic reticulum export ERE motif^36,42^ (M_EAAAK:ERE_), which approximately doubled surface SNAP-tag expression (Supplementary Fig. 10h). Remarkably, P-D1_ago_ tethered to M_EAAAK:ERE_ (MP-D1_ago_) that was stimulated by blue light activated D1R to near maximal possible levels (elicited by saturating, 10 μM, DA) (Fig. 2b, d, e) without altering baseline activation in the “off”-state, under UV light (Supplementary Fig. 10i–k). GIRK current elicited by MP-D1_ago_ photo-activation and photo-deactivation of D1R rose and fell in seconds (Fig. 2f), with no loss in efficacy over multiple cycles (Fig. 2f), and in a bistable manner, i.e., the “on”-state was stable in the dark following a short flash of blue light and the “off”-state was stable after a short flash of UV light (Fig. 2g). This contrasts with slow off-kinetics and rundown of opto-D1R (Supplementary Fig. 11a–e)^43^. MP-D1_ago_ was ~100-fold more sensitive to light than ChR2 (Fig. 2h and Supplementary Fig. 11f, g)^44^.

We assessed the specificity of MP-D1_ago_ by profiling its effects on a variety of neuronal receptors, each co-expressed in HEK293T cells with the M. We first tested D5R, the closest homolog of D1R. D5R was photoactivated by MP-D1_ago_ to about half the level of D1R (Fig. 2i; Supplementary Fig. 12a). We also evaluated other receptors that are co-expressed with D1R in the brain, including GPCRs and ionotropic receptors for DA, adenosine, cannabinoids, acetylcholine, opioids, glutamate, and GABA. MP-D1_ago_ had no effect on any of these receptors (Fig. 2i; Supplementary Fig. 12b–m), indicating that it is a D1R/D5R-selective photo-agonist.

### Activation of D1Rs in the dorsal striatum with MP-D1_ago_ promotes movement initiation

**Fig. 3:**
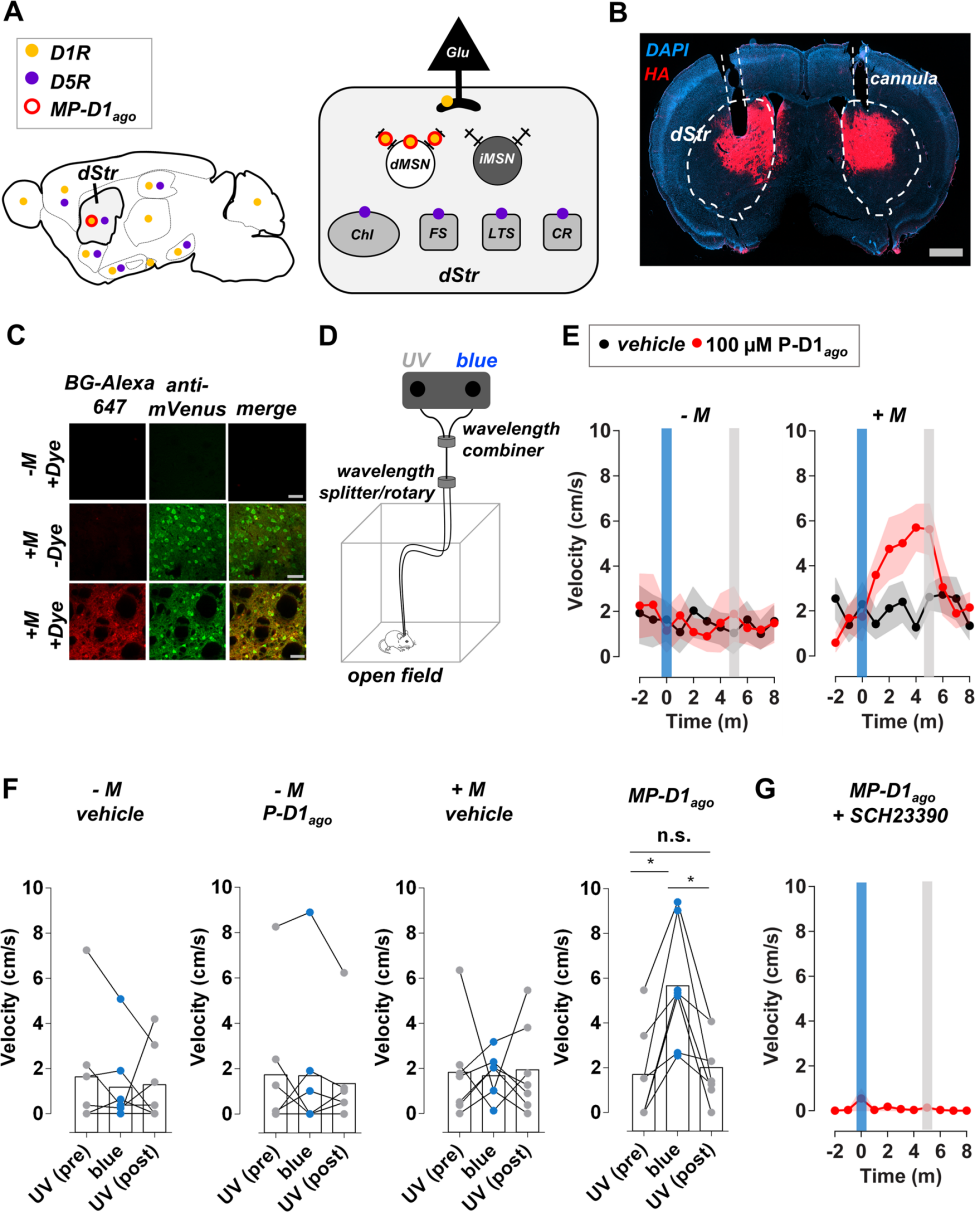
Activation of D1Rs in MSNs of the DMS with MP-D1_ago_ enhances movement. **A** D1R and its close homolog D5R are widely expressed in the brain (left panel) and are present in multiple cell types in the dorsal striatum (dStr; right panel). D1R is also expressed in the terminals of some glutamatergic afferents that innervate the striatum. DMS-dMSN D1Rs were selectively targeted with MP-D1_ago_ (red), which is composed of the membrane-anchor M_EAAAK:ERE_ and P-D1_*ago*_. **B** The M was virally delivered to DMS-dMSNs. Its expression (red = HA-tag staining) can be seen within the dStr. P-D1_ago_ and light were delivered the dStr with a reversible infusion/optical cannula. grey bar = 100 µm. blue = DAPI staining. Representative of brains from *n* = 7 mice. **C** The M is present at the surface of dStr-dMSNs according to its ability to bind the impermeant SNAP-tag binding dye SNAP-Surface Alexa Fluor 647. Labeling was not observed in the absence of the M or dye. mVenus (green) is an indicator of viral infection. grey bars = 50 µM. Representative of brains from *n* = 3 mice. **D** D1-Cre mice injected with an AAV encoding mVenus or the M and mVenus received bilateral dStr-infusions (1 µL) of either vehicle or the inactive form of P-D1_*ago*_ (cis; 100 µM) three hours prior to being placed in an open field. Locomotor activity of MP-D1_ago_ mice was measured in an open field. UV light and blue light were delivered to both sides of the brain. **E** The speed of mice with MP-D1_ago_ in dStr-dMSNs increased in response to a brief flash (450 nm, ~6 mW, 1 s) of blue light and returned to baseline after a brief flash (375 nm, ~9 mW, 1 s) of UV light. The behavioral response was not observed in mice lacking the M and/or P-D1_*ago*_ (400 nL infusion in each hemisphere). **F** The speed of each mouse was averaged over the following two-minute periods: (i) just before exposure to blue light (*UV pre*), (ii) three minutes after exposure to blue light (*blue*), (iii) and one minute after exposure to the second flash of UV light (*UV post*). See Supplementary Fig. 15a for more detail. Shown is a summary of the speed of -*M* or +*M* mice treated with vehicle or P-D1_*ago*_ mice during the *UV pre*, *blue*, and *UV post* periods. RM one-way ANOVA, F-values from left to right: 0.2, 0.4, 0.1, 15.4, Bonferroni, *p < 0.05. *n* = 7 mice for each condition. **G** The behavioral photoeffect was not observed in the presence of the D1R antagonist SCH23390 (0.5 mg/kg). Error bars indicate S.E.M.

The dStr expresses D1R and D5R (Fig. 3a), which are notoriously difficult to distinguish with conventional pharmacology^25^. However, dStr D1R and D5R are segregated into distinct classes of neurons: D1R is found in all dMSNs and the terminals of some glutamatergic afferents that innervate the dStr, whereas D5R is found in four types of interneurons (Fig. 3a)^1^. Thus, the targeted delivery of MP-D1_ago_ to dStr-dMSNs in combination with its rapid on and off kinetics would allow us to control of D1R in the dStr with specificity and determine its effect on movement initiation.

The gene encoding the M component of MP-D1_ago_, M_EAAAK:ERE_, was delivered to dStr-dMSNs of D1-Cre mice (Fig. 3a) using a Cre recombinase (Cre)-dependent adeno-associated virus (AAV) that was stereotaxically injected (Supplementary Fig. 13a, b). An AAV containing only mVenus was used as a control. The M expressed throughout the dStr (Fig. 3b and Supplementary Fig. 13c), on the cell surface of dStr-dMSNs (Fig. 3c), and alongside endogenous D1Rs in the soma and neurites (Supplementary Fig. 13d)—prerequisites for MP-D1_ago_ to interact with its target receptor. While we observed no obvious changes in morphology of striatal cells expressing the M, and our recent study of cultured cortical neurons expressing a similar M showed normal resting properties, action potential firing and neuromodulation^36^, it will be important to measure the properties of striatal neurons to assess whether M impacts their physiological parameters.

Mice expressing the M in dStr-dMSNs were infused with the UV-light induced inactive configuration of P-D1_*ago*_ (*cis*). P-D1_*ago*_ was infused into the dStr using conditions that were optimized for maximum conjugation to the M (Supplementary Fig. 14a, b). Locomotion was measured in an open field under conditions of optimized bilateral illumination (Fig. 3d and Supplementary Fig. 14c–h). Locomotion of mice with MP-D1_ago_ increased following a flash of blue light (1 s) and returned to baseline following a flash of UV light (1 s; Supplementary Fig. 15a), an effect that was robust (Fig. 3e and Supplementary Fig. 15b, c) and repeatable (Supplementary Fig. 15d). The behavioral photo-effect was not observed in the absence of either the M or P-D1_*ago*_ (Fig. 3e, f). We also tested MP-D1_ago_ in the presence of the D1R antagonist SCH23390, which suppresses locomotion on its own^45^. The blue-light induced promotion of locomotion by MP-D1_ago_ was blocked by SCH23390 (Fig. 3g and Supplementary Fig. 15e), consistent with a requirement for D1R to confer the MP-D1_ago_ effect, though complicated by the suppression of baseline locomotion by SCH23390.

**Fig. 4:**
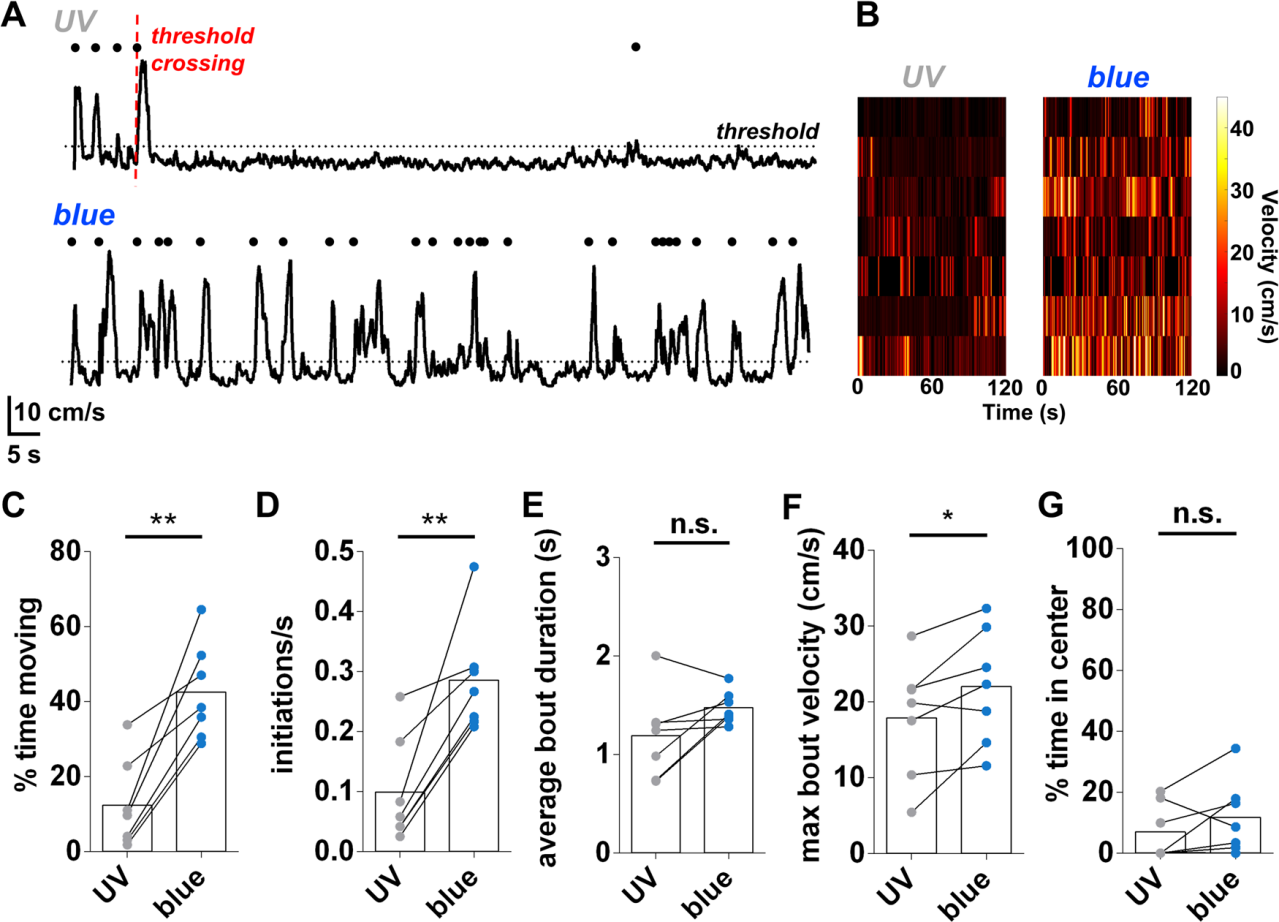
dStr-dMSN D1R activation with MP-D1_ago_ enhances movement initiation but has no effect on movement duration. **A** Example movement dynamics of a mouse with MP-D1_ago_ in the inactive state (UV light; *top panel*) and active state (blue light; *bottom panel*), which correspond with the two-minute *UV pre* and *blue* periods described in Fig. 3f and Supplementary Fig. 15a. The dotted lines represent the velocity threshold used to identify movement bouts. Movement initiations are indicated by black circles. **B** Summary of the movement dynamics of mice with MP-D1_ago_ under UV light and blue light. Shown for MP-D1_ago_ mice is the effect of UV light and blue light on the **C** average percent of the time in motion, **D** average initiations per second, **E** average movement bout duration, **F** the average maximum movement bout velocity, and **G** percentage of the time that the mice were in the center of the open field. paired two-tailed t-test, *p*-values from left to right: **0.001, **0.003, 0.080, *0.025, 0.214. *n* = 7 mice for each condition.

To better understand the basis of the locomotor response to photo-activation of D1R in the dStr, we measured the locomotion dynamics of mice as we photo-switched MP-D1_ago_ from the inactive state (UV illumination) to the active state (blue illumination; Fig. 3e). The mice locomoted for a greater percentage of the time under blue light (Fig. 4a–c). The increase in locomotion was due to an increase in movement initiation (Fig. 4d) without a change in movement bout duration (Fig. 4e). There was also a modest increase in the maximum speed of motion in response to blue light (Fig. 4f). None of these differences were observed in mice lacking MP-D1_ago_ (Supplementary Fig. 16), and there was no difference in the time spent in the center of the open field under UV light and blue light (Fig. 4g). Therefore, activation of D1R in in the dStr increases locomotion by increasing the probability of movement initiation.

We also evaluated the longer-term effect of MP-D1_ago_ on D1Rs in the dStr by infusing the active configuration of P-D1_ago_ (*trans*), which is stable in the dark (Supplementary Fig 17a). The motor activity of mice expressing the M increased within 30 min of the start of infusion of *trans-*P-D1_ago_, remained high for many hours, and returned to baseline over a two-day period (Supplementary Fig. 17b). We conjecture that the delayed activation reflects the time it takes for P-D1_ago_ to conjugate to the M. The slow decay of the *trans*-MP-D1_ago_ effect matches that observed with a membrane-anchored AMPA receptor DART antagonist in dStr-dMSNs^35^, likely reflecting the turnover of the membrane anchor in these neurons. However, chronic D1R activation could also result in plasticity changes of dStr-dMSNs or network effects that return locomotion to baseline. Nevertheless, consistent with the acute effect of MP-D1_ago_, chronic dStr-dMSN D1R activation resulted in a selective enhancement in movement initiation (Supplementary Figs. 17c–f). Thus, MP-D1_ago_ can be used to photo-activate D1Rs in the dMSNs of the dStr either acutely or persistently.

### Comparison of MP-D1_ago_ with optogenetic activation of a dopaminergic projection to the dorsal striatum

**Fig. 5:**
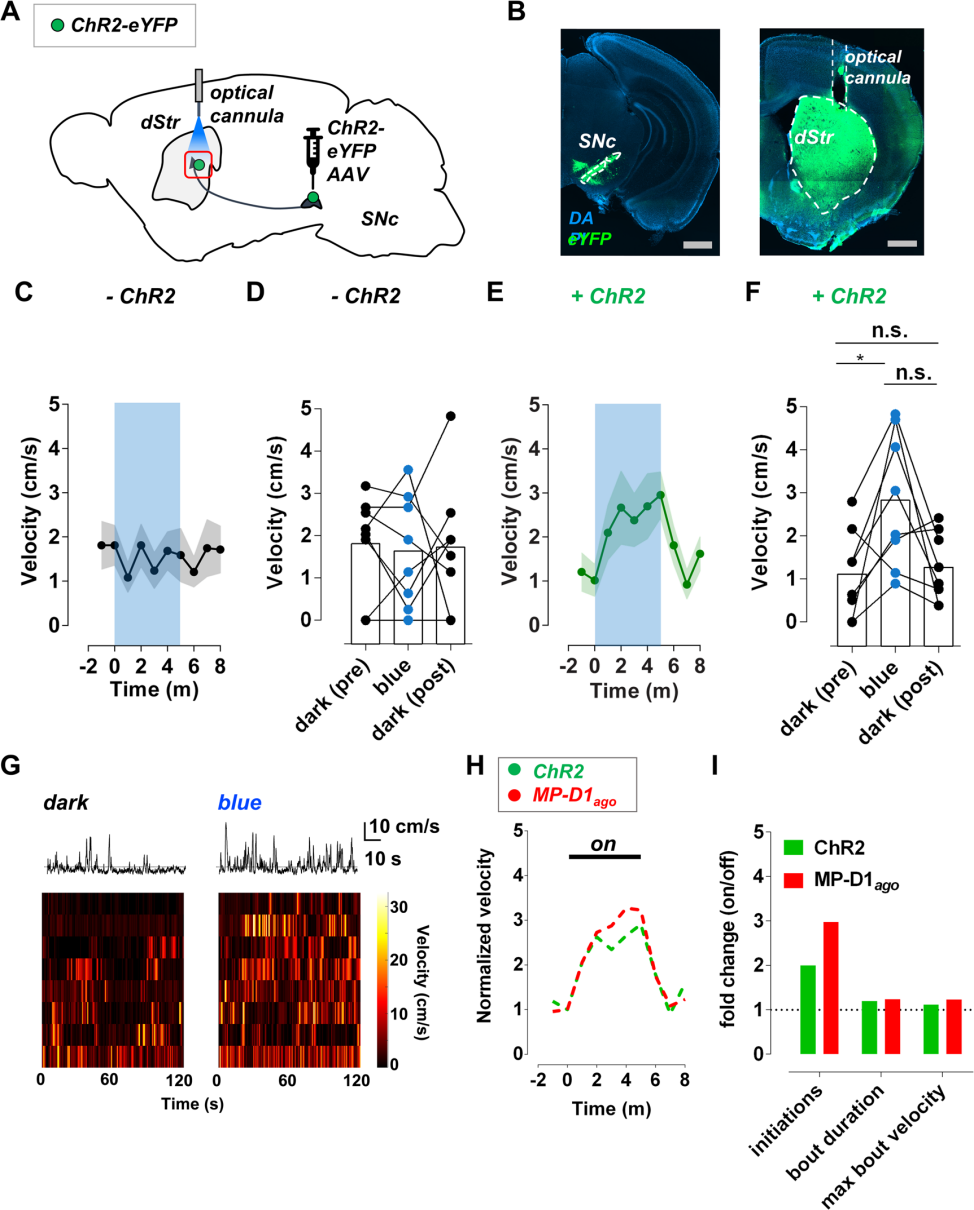
Comparison of the motor effect of optogenetic activation of SNc terminals in the dStr with ChR2 to dStr-dMSN D1R activation with MP-D1_ago_. **A** A Cre-dependent AAV encoding channelrhodopsin-2 (ChR2), AAV-EF1a-DIO-hChR2(H134R)-eYFP, was bilaterally injected into the substantia nigra compacta (SNc) of DAT-Cre mice, resulting in ChR2-eYFP expression in SNc DA neurons. To enhance neurotransmitter release from SNc DA neuron terminals onto the dorsal striatum (dStr), a bilateral optical cannula was implanted in the dStr. **B** Expression of ChR2-eYFP in DA neurons in SNc (*left panel*) and their terminals in the dStr (*right* panel). grey bars = 1 mm. blue = DAPI staining, green = eYFP (ChR2) staining. **C** The speed of mice lacking ChR2 was unaffected by blue light (450 nm, ~7 mW, 3 ms pulses at 20 Hz). **D** The speed of each mouse was averaged over the following two-minute periods: (i) just before exposure to blue light (*dark pre*), (ii) three minutes after exposure to blue light (*blue*), (iii) and one minute after exposure to blue light (*dark post*). See Supplementary Fig. 18b for more detail. Shown is the summary of the speed of mice lacking ChR2 during the *dark pre*, *blue*, and *dark post* periods. RM one-way ANOVA, F = 0.1, Bonferroni. *n* = 8 mice. **E** The speed of mice with ChR2 in SNc DA neuron terminals increased in response blue light and returned to baseline in the dark. **F** Summary of the speed of ChR2 mice during the *dark pre*, *blue*, and *dark post* periods. RM one-way ANOVA, F = 6.3, Bonferroni, *p < 0.05. *n* = 8 mice. **G** Example movement dynamics of a mouse with ChR2 in the inactive state (dark) and active state (blue light), which correspond with the *dark pre* and *blue* periods described above (*top panels*). The dotted lines represent the velocity threshold used to identify movement bouts. Summary of the movement dynamics of mice with ChR2 in the dark and in response to blue light (*bottom panels*). **H** The behavioral response to ChR2 activation in SNc DA neuron terminals in the dStr versus dStr-dMSN D1R activation with MP-D1_ago_. ChR2 data from Fig. 5e and MP-D1_ago_ data from Fig. 3e were normalized to baseline movement and replotted. There was no significant difference between groups. RM two-way ANOVA. *p* = 0.99. *n* = 8 for ChR2 and *n* = 7 for MP-D1_ago_. **I** Summary of the relative difference in movement initiation, bout duration, and maximum bout velocity for mice with ChR2 or MP-D1_ago_ in their active and inactive states. ChR2 data from Supplementary Fig. 18d–f and MP-D1_ago_ data from Fig. 4d–f were normalized to baseline and replotted. Error bars indicate S.E.M.

We wondered how the behavioral effect of photo-activation of D1Rs in the dMSNs of the dStr with MP-D1_ago_ compares with that elicited by activity in dopaminergic inputs to the dStr, which is expected to activate not only dMSN D1Rs but also DARs in each cell type of the dStr, as well as GABA receptors (Supplementary Fig. 1e). To test this, a Cre-dependent AAV encoding ChR2-eYFP was injected into the SNc of DAT-Cre mice. Robust eYFP expression was observed in SNc DA neurons, the major DA input to the dStr^46^, and in the dStr, where SNc DA neurons make their axon terminal beds (Fig. 5b and Supplementary Fig. 18a). As described earlier^6^, activating ChR2 in SNc DA axon terminals in the dStr enhanced locomotion in the open field (Fig. 5c–f and Supplementary Fig. 18b). The enhanced locomotion was due to an increase in movement initiation, with no change in the duration of movement bouts (Fig. 5g and Supplementary Fig. 18c–f). The timing and magnitude of these effects closely resembled those elicited by photo-activation of D1Rs in the dMSNs of the dStr with MP-D1_ago_ (Fig. 5h, i). This suggests that activation of D1Rs in the dMSNs of the dStr accounts for a significant portion of the ability of SNc DA neurons to enhance movement.

### Comparison of MP-D1_ago_ with a conventional D1R agonist

We compared the selective activation of D1R in dStr-dMSNs with MP-D1_ago_ to infusion into the dStr of the conventional full D1R agonist SKF82958, which is expected to also activate D5R in multiple cell types. Like MP-D1_ago_, SKF82958 enhanced locomotion (Supplementary Fig. 19b–c). However, locomotion increased ~12-times faster with MP-D1_ago_ than with SKF82958 (Supplementary Fig. 19d), reflecting the temporal difference between light propagation (fast) and pharmacologic diffusion (slow) in brain tissue. Moreover, although both increased the percentage of the time that the mice were in motion (Supplementary Fig. 19e, f), MP-D1_ago_ selectively increased the probability of movement initiation (Fig. 4), whereas SKF82958 increased both movement initiation and the duration of the movement bouts (Supplementary Fig. 19g, h). This difference indicates that locally infused DAR ligands may not faithfully mimic the actions of dopaminergic input, either because of differences in the activation of different DAR subtypes and cell types or because they diffuse to other brain areas (Supplementary Fig. 19a).

### D1Rs in the nucleus accumbens have no effect on movement

Whereas the dStr is specialized for movement control, the nucleus accumbens (NAc) is associated with reward^6^ and motivation^47^. However, studies suggest that dopaminergic input from the ventral tegmental area (VTA) to the NAc can also promote movement, possibly by increasing motivation and decreasing work-related response costs^47,48^ or promoting motor learning^49^. D2R in indirect pathway-MSNs of the NAc (NAc-iMSNs) promotes locomotion in an open field^50^. By contrast, the effect of D1R in the NAc on locomotion is less clear because existing methods have produced contradictory results^43,51–53^. To explore this, we probed the effect of MP-D1_ago_ photo-activation in dMSNs of the NAc (NAc-dMSNs). We observed no effect on locomotion of switching fiber illumination from UV to blue light (Supplementary Fig. 20), suggesting that motor activation in the NAc is not driven by dMSN D1R.

## Discussion

**Fig. 6:**
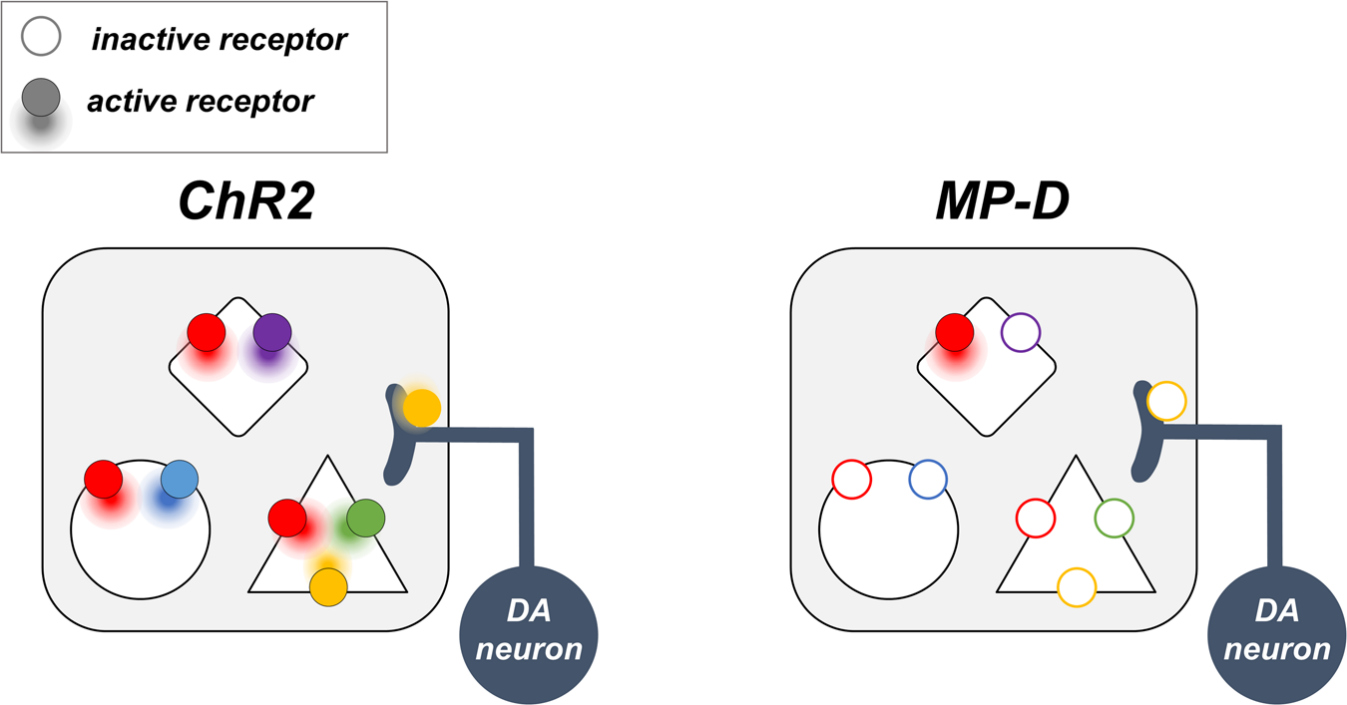
Comparison of the effect of an excitatory opsin and MP on dopamine circuits. The excitation of dopaminergic neurons with ChR2 enhances neurotransmitter release onto the target brain area, resulting in the activation of multiple receptors in various combinations in multiple cell types (left panel). In contrast, an MP can be used to activate just one of the targets of those neurons (right panel). Each color represents a particular receptor subtype.

DA plays central roles in behavior. To understand DA neuromodulation, we need to be able to control the activation of specific DARs in specific cell types and brain regions with temporal precision. Optogenetic tools like ChR2 tell us how behavior changes in response to the activation of specific DA neurons, providing a cellular view of DA action in the brain. MPs offer a complementary perspective: they tell us how behavior changes in response to the activation of just one of the many targets of those DA neurons (Fig. 6). MPs are uniquely suited to this task because they control endogenous DARs with receptor subtype, cell type, and spatiotemporal specificity. We present here an initial demonstration that the MP approach can modulate neuromodulatory receptors affecting behavior. An important caveat of this work is that the neural effects in vivo have not been investigated.

The activity of SNc neuron axons that release DA onto to the dStr promotes movement initiation^11^^,^^54^. SNc DA neurons co-release glutamate and GABA^55,56^, which may contribute to the effect. Moreover, multiple DAR subtypes in various combinations in each of the cell types of the dStr may be activated by DA released from SNc axon terminals (Supplementary Fig. 1e).

We used MP-D1_ago_ to bypass the SNc DA neurons and directly activate a DAR on one SNc target: the D1R of dStr-dMSNs. To do this, we delivered the gene encoding M using AAV under Cre control into the dStr of D1-Cre mice to yield selective expression in dStr-dMSNs. Following expression, we infused P-D1_ago_ into the same site. Illumination to photo-activate D1_*ago*_ was found to promote locomotion as effectively, with the same selective boost of movement initiation, and with the same kinetics as optogenetic stimulation of SNc DA axon terminals. This supports the notion that dMSN D1R is the key target of DA in the dStr that contributes to movement initiation. This could not be ascertained with the conventional D1R agonist SKF82958, which does not match the actions of dopaminergic input from the SNc to the dStr, likely due to a lack of molecular, cell type, and/or spatiotemporal precision.

D1R activation in response to endogenous DA or synthetic D1R agonists increases dMSN excitability^18–22^, which is associated with enhanced movement^12–14^. Electrophysiological analysis of the effect of MP-D1_ago_ on dMSN physiology will be required to demonstrate that this photo-activation of D1R enhances movement through the same mechanism and to determine whether M expression affects the physiological properties of dMSNs. Relevant to this, a similar M was not found to perturb cultured cortical neurons^36^. Nevertheless, MP-D1_ago_ could provide insight into the effect of D1R activation on dMSN excitability and ion channels such as NMDA receptors with greater selectivity and temporal precision than that used in previous studies^22,55^.

We found that both the optogenetic stimulation of SNc terminals and the direct photo-activation of dMSN D1Rs with MP-D1_ago_ increase movement gradually over minutes. This contrasts with the rapid second-timescale kinetics of DA release in the dStr, as detected by voltammetry^22^ and optical reporters^54,56,57^, and of D1R effector activation and deactivation by MP-D1_ago_ in cultured cells that we report here. The mismatch is consistent with dStr-dMSN D1R signaling increasing the response to locomotor drive from other inputs, such as projections from motor cortex. Although we have not deployed it here, the ability to rapidly turn MPs on and off provides the opportunity to establish the dependence of behavioral output on the temporal dynamics of activating specific DARs in specific striatal cells. In principle, this could apply to movement, Pavlovian conditioning, or reward prediction error, wherein extracellular DA rises transiently for less than a second to tens of seconds^54^.

Recent work indicates that adjacent subregions of the dStr have distinct functional properties^58,59^. Whereas the dorsomedial striatum (DMS) controls the initial stages of motor learning, the dorsolateral striatum (DLS) facilitates the acquisition of habitual and automatic behavior^60,61^. Unlike diffusible conventional agonists or antagonists, our approach could be used to selectively target specific DAR subtypes in specific subregions of the dStr by limiting the expression of the M component of the MP.

A major hurdle in the study of DA has been our inability to selectively target specific DARs. For example, there are currently no conventional agonists and antagonists that can differentiate between these D1R and D5R^17^ due to the high similarity between these receptors^21^. In recent years considerable effort has gone towards developing allosteric modulators of D1R, which bind non-conserved regions and thus are more likely to be receptor selective. However, existing allosteric modulators are not sufficiently D1R-selective^62^ or have very low affinity for rodent D1R^63,64^ so are not compatible with rodent studies. In contrast, MPs can bestow receptor subtype selectivity onto otherwise non-selective ligands by taking advantage of the inherent cellular segregation of closely related DARs in DA neurons. MP-D1_ago_, which contains a non-selective D1R/D5R agonist, was restricted to striatal dMSNs that express D1R but not D5R. A similar strategy could be used to target D2R, for which there are no highly selective ligands due to its high homology with D3R^65^. For example, a MP that contains an inherently non-selective D2R/D3R ligand could be used to selectively control D2R in striatal iMSNs because these neurons lack D3R (Supplemental Fig. 1e). Although the MP approach can take advantage of the cellular segregation of closely related receptors, the development of MP analogs that contain DAR subtype selective ligands will be need for cases when closely related receptors are expressed in the same cells.

Optogenetics tools like ChR2 require one component (Supplementary Fig. 2), a genetically encoded protein, and light. MPs require an additional custom chemical component. Considering that DARs have similar architecture, we expect that modular changes to the agonist moiety of P-D1_ago_ will make it possible to create a repertoire of ligands, each with narrow action, but which, as a group, cover a wide range of functionalities. Like optogenetic stimulation, DAR activation with MP agonists, like MP-D1_ago_, is an unnatural manipulation that occurs separately from the actions of DA. Substitution of the PPHT agonist moiety of MP-D1_ago_ with an MP antagonist or negative allosteric modulator could provide the advantage of rapid and reversible block of receptor activation in response to natural DA signals. Furthermore, just as DAR subtypes are targeted to various subcellular compartments, MPs could be modified through the addition of genetically-encoded targeting motifs to the M to enable additional experimental specificity. For all of these applications, it will be important in future work to characterize how MP-Ds photo-modulate the biochemical and physiological signaling of cells in both brain slice and in vivo.

With the current DAR MP and the recent MP for mGluRs^36^, we have shown the approach to work for both Family A and Family C GPCRs, suggesting that the approach will be generalizable to diverse neuronal receptors and channels. Given that the problem of receptor complexity is not unique to DA circuits, MPs have the potential to be used as a general approach to crack neural circuits and their relationship to behavior.

## Methods

### Chemistry

All reactions were carried out with magnetic stirring, and, if moisture- or air- sensitive, under nitrogen or argon atmosphere using standard Schlenk techniques in oven-dried glassware (140 °C oven temperature). External bath temperatures were used to record all reaction temperatures. Low temperature reactions were carried out in a Dewar vessel filled with distilled water/ice (0 °C). High temperature reactions were conducted using a heated silicon oil bath in reaction vessels equipped with a reflux condenser or in a sealed flask. Tetrahydrofuran (THF) was distilled over sodium and benzophenone prior to use. Dichloromethane (CH_2_Cl_2_), triethylamine (Et_3_N) and diisopropylethylamine (DIPEA) were distilled over calcium hydride under a nitrogen atmosphere. All other solvents were purchased from Acros Organics as ‘extra dry’ reagents. All other reagents with a purity >95% were obtained from commercial sources (Sigma Aldrich, Acros, Alfa Aesar, Base Click, and others) and used without further purification.

Flash column chromatography was carried out with Merck silica gel 60 (0.040–0.063 mm). Analytical thin layer chromatography (TLC) was carried out using Merck silica gel 60 F254 glass-backed plates and visualized under UV light at 254 nm. Staining was performed with ceric ammonium molybdate (CAM) or by oxidative staining with an aqueous basic potassium permanganate (KMnO_4_) solution and subsequent heating. HPLC was performed with HPLC grade solvents and deionized H_2_O that was purified on a TKA MicroPure H2O purification system. All solvents were degassed with helium gas prior to use. Unless noticed otherwise, all experiments were carried out at room temperature.

NMR spectra (^1^H NMR and ^13^C NMR) were recorded in deuterated chloroform (CDCl_3_) or deuterated methanol (*d*_*4*_*-*MeOH) on a Bruker Avance III HD 400 MHz spectrometer equipped with a CryoProbe™, a Varian VXR400 S spectrometer, a Bruker AMX600 spectrometer or a Bruker Avance III HD 800 MHz spectrometer equipped with a CryoProbe™ and are reported as follows: chemical shift *δ* in ppm (multiplicity, coupling constant *J* in Hz, number of protons) for ^1^H NMR spectra and chemical shift *δ* in ppm for ^13^C NMR spectra. Multiplicities are abbreviated as follows: s = singlet, d = doublet, t = triplet, q = quartet, quint = quintet, br = broad, m = multiplet, or combinations thereof. Residual solvent peaks of CDCl_3_ (*δ*H = 7.26 ppm, *δ*C = 77.16 ppm) and *d*_*4*_*-*MeOH (*δ*H = 3.31 ppm, *δ*C = 49.00 ppm) were used as an internal reference. NMR spectra were assigned using information ascertained from COSY, HMBC, HSQC, and NOESY experiments.

High resolution mass spectra (HRMS) were recorded on a Varian MAT CH7A or a Varian MAT 711 MS instrument by electron impact (EI) or electrospray ionization (ESI) techniques at the Department of Chemistry, Ludwig-Maximilians-University Munich. Infrared spectra (IR) were recorded from 4000 cm^−1^ to 600 cm^−1^ on a PERKIN ELMER Spectrum BX II, FT-IR instrument. For detection, a SMITHS DETECTION DuraSampl*IR* II Diamond ATR sensor was used. Samples were prepared as a neat film or a thin powder layer. IR data in frequency of absorption (cm^−1^) is reported as follows: w = weak, m = medium, s = strong, br = broad, or combinations thereof. P-D1_*ago*_ is available from the authors upon reasonable request.

### Structural modeling

A homology model of D1R was generated using Bioluminate (Schrodinger, Inc)^66^. The model was based on β2-adrenergic receptor (β2AR) because of the high relative degree of homology between D1R and β2AR. The amino acid sequence of D1R was aligned to β2AR in BLAST^67^ and the TM segments were then structurally aligned with the crystal structure of β2AR bound to the inverse agonist carazolol (pdb: 2RH1). The three extracellular loops of D1R were refined using extended sampling. For docking DA into D1R, DA was prepared using LigPrep (Schrodinger, Inc) and docked with extra precision (XP) using Glide^68^. The hydroxyl groups of the three TM5 serines (Ser194^5.42^, Ser195^5.43^ and Ser199^5.46^) in D1R that contribute to the OBS were allowed to rotate during the docking procedure. As expected, the protonated amine and hydroxyls of DA were oriented towards Asp100^3.32^ in TM3 and the TM5 serines, respectively.

To dock the P-D1_ago_ analog PEG_4_-azobenzene-PPHT in the D1R model, the protonated ligand was first prepared in ChemDraw (PerkinElmer) and Chimera (developed by the Resource for Biocomputing, Visualization, and Informatics at the University of California, San Francisco and supported by NIGMS P41-GM103311). It was then docked in the D1R model using Autodock Vina^69^. The following orthosteric binding site residues in D1R^70^ were allowed to rotate during the docking procedure: Asp100^3.32^, Ile101^3.33^, Ser104^3.36^, Ser194^5.42^, Ser195^5.43^, Ser199^5.46^, Trp282^6.48^, Phe285^6.51^, Phe286^6.52^, Asn289^6.55^, Val314^7.39^, and Trp318^7.43^.

To measure the distance from the benzylguanine binding site in the membrane-anchor (M) to the ligand binding site in D1R or mGluR5, a model of the M was made by placing the C-terminus of SNAP-tag (pdb: 3L00) flush with the N-terminus of a single-pass transmembrane segment (pdb: 2K1A). The M was then positioned adjacent to mGluR5 or D1R. The benzylguanine binding site in the M was oriented facing towards the receptor ligand binding site. To measure the lengths of P-D1_ago_ or BGAG, their extended states were first prepared in ChemDraw and end-to-end distances for each chemical component were measured in Chimera.

All molecular representations were prepared using Chimera.

### Molecular biology and heterologous expression

All constructs are in mammalian expression vectors. For the receptor mediated-GIRK activation assay, HEK293T cells were seeded onto 18 mm coverslips and transiently transfected overnight with Lipofectamine 2000 and the following constructs: a receptor, a variant of the membrane-anchor (M; 0.7 µg), GIRK1(F137S) (0.7 µg), and tdTomato (0.2 µg). The following receptors were tested: D1R (0.525 µg), opto-D1R (0.7 µg), D3R (0.2 µg), D5R (0.35 µg), A1R (0.7 µg), CB1R (0.7 µg), M1R (0.7 µg), M4R (0.7 µg), MOR (0.7 µg), mGluR1 (0.7 µg), and GABA_B_R (0.7 µg of each subunit, B1 and B2). Some of these receptors do not couple to GIRK channels in HEK293T cells unless coexpressed with certain Gα subunits. D1R, D5R, or opto-D1R were cotransfected with Gα_is13_ (0.35 µg), D3R with Gα_oA_ (0.7 µg), and M1R or mGluR1 with Gα_iq5_. Transfected cells were used for electrophysiology, imaging, or flow cytometry experiments.

For ligand gated-ion channel (LGIC) electrophysiology, HEK293T cells were seeded onto 18 mm coverslips and transiently transfected for 48 h with Lipofectamine 2000 and the following constructs: MA_EAAAK:ERE_ (0.7 µg), tdTomato (0.2 µg), and one of the following: a non-desensitizing mutant of AMPAR GluR_A1_ (L497Y; 1.4 µg), the kainite receptor Glu_K2_ (0.7 µg), or GABA_A_R_α1β2γ2_ (0.2 µg α1: 0.2 µg β2: 0.7 µg γ2).

### Confocal imaging and flow cytometric analysis of cultured cells

Untransfected or membrane-anchor (M) expressing HEK293T cells were labeled with 1.5 µM BG-TMR or BG-Alexa647 (NEB) for 20–30 min in the dark at 37 °C and 5% CO_2_ in a standard extracellular solution. Cells were then (i) mounted on an upright, scanning confocal microscope (Zeiss LSM 780) and imaged with a 20× objective or (ii) washed in DPBS and measured by flow cytometry (BD LSR II) using FACSDiva 6.0 software (BD Biosciences).

### Electrophysiology

HEK293T cells were sparsely seeded and maintained in DMEM (Invitrogen) with 10% fetal bovine serum on poly-L-lysine-coated coverslips at 37 °C and 5% CO_2_. HEK293T cells were voltage clamped in whole-cell configuration 16–48 h after transfection. For GIRK experiments, the extracellular solution contained 120 mM KCl, 25 mM NaCl, 10 mM HEPES, 2 mM CaCl_2_, and 1 mM MgCl_2_, pH 7.4. opto-D1R cells were preincubated for 45 min with and patched in the presence of 1 µM 11-cis-retinal. For LGIC experiments, the extracellular solution contained 135 mM NaCl, 5.4 mM KCl, 10 mM HEPES, 2 mM CaCl_2_, and 1 mM MgCl_2_, pH 7.4. Glu_K2_ electrophysiology was performed in the presence of 0.3 mg/mL concanavalin A to prevent desensitization. Glass pipettes with a resistance of 3-7 MΩ were filled with intracellular solution containing 120 mM Gluconic acid δ-lactone, 15 mM CsCl, 10 mM BAPTA, 10 mM HEPES, 1 mM CaCl_2_, 3 MgCl_2_, 3 mM MgATP, pH 7.2. Cells were voltage clamped to −60 or −80 mV using an Axopatch 200 A (Molecular Devices) amplifier.

To conjugate P-D1_ago_ to variants of the membrane-anchor (M), cells were incubated with varying concentrations of P-D1_ago_ for up to 60 min in the dark at 37 °C in standard extracellular buffers. For all experiments, compounds were applied using a gravity-driven perfusion system and illumination was applied to the entire field of view using a Polychrome V monochromator (TILL Photonics) through a 20× objective (maximum 0.05 mW/mm^2^ at 370 nm and 0.5 mW/mm^2^ at 460 nm or 473 nm) or a DG4 (Sutter) through a 20× objective (maximum 0.06 mW/mm^2^ at 370 nm and 0.6 mW/mm^2^ at 445 nm). pClamp software (Clampex 10; Axon Instruments) was used for both data acquisition and control of illumination.

The selection criteria for electrophysiological experiments are that a cell (i) expresses the fluorescent protein transfection marker, and (ii) responds to agonist, indicating the presence of either receptor and GIRK or an LGIC. We did not exclude cells unless the recording was of poor quality (e.g., unstable baseline).

### Animal model

The following mouse lines were used for the experiments: D1-Cre (GENSAT, stock number: 017264-UCD, strain code: Tg(Drd1-cre)EY262Gsat/Mmucd) and DAT::IRES-Cre (Jackson Laboratory, stock number: 006660, strain code: B6.SJL-Slc6a3tm1.1(cre)Bkmn/J). The mice were 20–30 grams and 8–20 weeks old. Males and females were counterbalanced across conditions with no effects of sex observed. Mice were maintained on a 12:12 light cycle (lights on at 07:00) at ambient temperature (20–25 °C) and humidity (30–70%). All procedures complied with the animal care standards set forth by the National Institutes of Health and were approved by University of California Berkeley’s Administrative Panel on Laboratory Animal Care.

### Light intensity calibration in brain

To calibrate the intensity of light applied to the brain for in vivo behavioral experiments with MP-D1_ago_, we followed a protocol similar that described for measuring blue light transmission through rodent cortex^71^. Because light transmission depends on wavelength^72^ and brain area^73^, we adjusted the protocol to measure the transmission of UV and blue light through striatal tissue. Mice were deeply anaesthetized with pentobarbital (200 mg/kg *i.p*.; Vortech) and then decapitated. Intracardial perfusion was not performed to avoid removing blood from the brain. Striatal tissue was excised and immediately placed in ice-cold artificial cerebrospinal fluid (ACSF) containing 50 mM sucrose, 125 mM NaCl, 25 mM NaHCO_3_, 2.5 mM KCl, 1.25 mM NaH_2_PO_4_, 0.1 mM CaCl_2_, 4.9 mM MgCl_2_, and 2.5 mM glucose (oxygenated with 95% O_2_:5% CO_2_). The tissue was then placed in an ACSF-filled petri dish directly above a power meter (Thorlabs) with a ~200 µM pinhole. Using a micromanipulator (Sutter), the tip of a fiber (200 µM, 0.22 NA) emitting 375 nm or 450 nm light was placed directly above the power meter pinhole and then progressively lowered in 200 µM steps through air or striatal tissue. Power intensity values of each wavelength at each depth were used to calculate % transmission, % initial power density, and light intensity at each depth.

### AAVs

The adeno-associated virus (AAV) encoding M_EAAAK:ERE_ and mVenus or mVenus alone were made in the Isacoff lab. These cDNAs were inserted into an established Cre-dependent double-floxed inverted orientation (DIO) viral cassette under control of the CAG promoter and then packaged in the AAV5 capsid. The purified vectors contained 10 viral genomes/ml. The AAV encoding ChR2, AAV-EF1a-DIO-hChR2(H134R)-eYFP, was obtained from the UNC vector core.

### Stereotaxic surgeries

All stereotaxic injections were performed under general ketamine–dexmedetomidine anesthesia using a stereotaxic instrument (Kopf Instruments, Model 1900). For AAV injections, 400 nL of concentrated viral solution was injected into either the dStr (bregma: 0.5 mm, lateral: ±1.9 mm, ventral: −3.0 mm), the NAc (bregma: 1.4 mm, lateral: ±1.0 mm, ventral: −4.1 mm), or the SNc (bregma: 3.3 mm, lateral: ±1.3 mm, ventral: −4.1 mm) using a syringe pump (Harvard Apparatus) at 150 nL/min. The injection needle was withdrawn 10 min after the end of each infusion. In some cases, immediately after viral injection mice were implanted with a bilateral guide cannula 0.5 mm dorsal to the injection site in the dStr or NAc. After injecting the ChR2 AAV into the SNc, a guide cannula was implanted in the dStr (bregma: 0.5 mm, lateral: ±1.9 mm, ventral: −2.5 mm). One layer of adhesive cement (C&B Metabond; Parkell) followed by cranioplastic cement (Dental cement) was used to secure the cannula to the skull. The incision was closed with tissue adhesive (Vetbond; 3 M). The animals were kept on a heating pad until they recovered from anesthesia. Experiments were performed 4–12 weeks after stereotactic injection. Injection sites and cannula placements were confirmed in all animals by preparing coronal or sagittal sections (50 μm) of the brain.

### Open field test

The open field test was conducted to measure locomotor ability. Mice were placed in a custom-made white polycarbonate open field chamber (20 × 20 × 20 inches). Their movement was recorded with a video camera (Logitech) at ~20–30 Hz and analyzed using the video-tracking software Viewer3 (Biobserve). Mice were subjected to one or more of the following prior to or during the behavioral test: (i) intraperitoneal injection, (ii) striatal infusion using a combination of infusion cannulas and a syringe pump (200 nL/min; Harvard Apparatus), and (iii) light stimulation using optical cannulas (200 µM, 0.22 NA). See figure legends for specifics on the dosing used for each experiment. For all behavioral experiments wherein MP-D1_ago_ was used, 1 nL of *P*-D1_ago_ (100 µM) was infused into both hemispheres of the brain. In all cases, mice were habituated to infusion, optical fibers, and the open field chamber (15 min sessions) for two days prior to the test day.

### Movement analysis

The sub-second velocity traces recorded using Viewer3 were smoothed in MATLAB using a first order Butterworth filter (normalized cutoff frequency ω_n_ = 0.02). Using original code written in MATLAB, a velocity threshold was manually set for each mouse to distinguish movements from tracking artifacts, as verified by visual inspection of each movie. The movement thresholds were similar across mice, e.g., the average threshold was 5.0 ± 0.7 cm/s for MP-D1_ago_ mice and 4.9 ± 0.3 cm/s for ChR2 mice. Movement bouts were counted when the velocity surpassed this threshold and lasted for at least 0.3 s. Bouts were considered finished after the velocity remained below the manually defined threshold for 0.1 s. The percent time moving was calculated as the time that velocity was above the movement threshold divided by the time of the entire measurement. Movement initiation frequency was calculated as the number of times velocity surpassed the movement threshold per second. Movement bout duration was calculated by averaging the duration of all movement bouts that occurred during the entire measurement. Max velocity was calculated by averaging the maximum velocity of all movement bouts that occurred during the entire measurement.

### Histology and confocal microscopy

After intracardial perfusion with 4% paraformaldehyde in PBS, pH 7.4, the brains were fixed overnight, placed in PBS with 30% sucrose for at least 24 h, and then sliced into 50 μm coronal or sagittal sections with a vibratome (Leica). The following primary antibodies were used: rabbit anti-HA tag (1:500; Cell Signaling), rat anti-D1R (1:500; Sigma-Aldrich), chicken anti-GFP/mVenus (1:1000; Abcam). The following secondary antibodies were used: goat anti-rabbit IgG-Alexa Flour 647 (1:750; Thermo Fisher Scientific), goat anti-rat IgG-Alexa 546 (1:750; Thermo Fisher Scientific), goat anti-chicken IgG-Alexa Flour 488 (1:1000; Thermo Fisher Scientific). Image acquisition was performed with a Zeiss LSM780 laser scanning confocal microscope using a 20× objective and on a Zeiss AxioImager M1 upright widefield fluorescence/differential interference contrast microscope with charge-coupled device camera using 5× objectives. Confocal images were collected using Zen Black 3.0 (Zeiss) and analyzed using Zen Blue 3.0 (Zeiss) and ImageJ. Sections were identified using landmarks and neuroanatomical nomenclature^74^. In some cases, AAV-injected mice received a dStr-infusion of 100 µM SNAP-Surface Alexa Fluor 647 (400 nL per hemisphere; NEB) followed by intracardial perfusion 24–48 h later.

### Statistics and data analysis

Data were analyzed using GraphPad Prism (GraphPad), Clampfit (Axon instruments), Origin 8E (OriginLab), MATLAB R2020a, or ImageJ 1.52b software. For dose-response curves, data were normalized to vehicle (0%) and DA (100%) and nonlinear regression analysis was performed using the sigmoidal dose-response function in GraphPad Prism. Statistical analyses were performed using GraphPad Prism. All values reported are mean ± S.E.M.

## Supplementary information

Supplementary Information

nr-reporting-summary

## Data Availability

Source data are provided with this paper. Additional data have been deposited in the Figshare repository under the filename MP-D_data.xlsx. MP-D1_*ago*_ and D1-Cre mice are available upon request from the authors. Source data are provided with this paper.

## Source data

Source data

## Code availability

The custom code written for this study are available in the GitHub repository under the filename BehavioralAnalaysis_Donthamsetti_2021.m.
