# Supplementary material for: Cell specific photoswitchable agonist for reversible control of endogenous dopamine receptors
Source: Nat Commun. 2021 Aug 6;12:4775. doi: 10.1038/s41467-021-25003-w (PMC8346604; doi:10.1038/s41467-021-25003-w)
Supplement: Supplementary file 1 — Supplementary Information [file 41467_2021_25003_MOESM1_ESM.pdf]

Supplementary Information

Cell specific photoswitchable agonist for reversible control of endogenous dopamine receptors

Prashant Donthamsetti[1], Nils Winter[2], Adam Hoagland[1], Cherise Stanley[1], Meike Visel[1], Stephan Lammel[1], Dirk Trauner[3], Ehud Isacoff[1,4,5]

[1]Molecular and Cell Biology, University of California, Berkeley, Berkeley, California, USA

[2]Department of Chemistry, Ludwig-Maximilians University, München, Germany

[3]Department of Chemistry, New York University, New York City, New York, USA

[4]Helen Wills Neuroscience Institute, University of California, Berkeley, California, USA

[5]Molecular Biophysics & Integrated Bioimaging Division, Lawrence Berkeley National Laboratory, Berkeley, California, USA

*e-mail: ehud@berkeley.edu

Supplementary Methods

All yields are isolated unless otherwise specified.

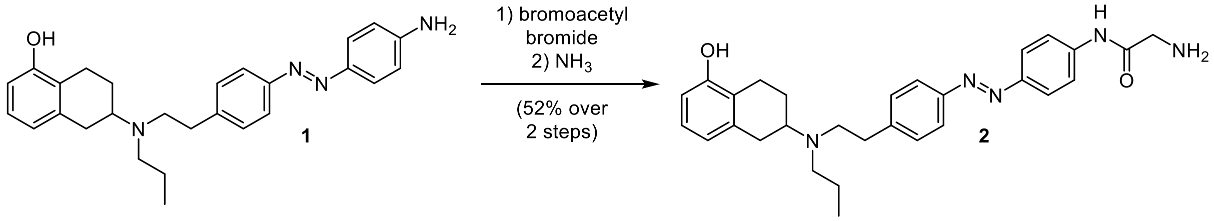

Amine 2: Bromoacetylbromide (0.06 mL, 0.699 mmol) was added dropwise to an ice cooled solution of phenol **1**[13] (214 mg, 0.499 mmol) and DIPEA (0.12 mL, 0.699 mmol) in THF (8 mL) and the mixture was stirred for 10 min at that temperature and for 8 h at room temperature. The reaction was diluted with EtOAc and washed with saturated aqueous $NaHCO_3$ and brine. The organic phase was dried over $MgSO_4$ and concentrated under reduced pressure. The resulting residue was redissolved in THF (11 mL) and cooled to 0 °C. Ammonia (7 M in MeOH, 4.00 mL, 28.0 mmol) was added and the mixture was stirred at room temperature overnight. The reaction was diluted with H_2O and extracted with EtOAc. The combined organic phases were washed with brine, dried over $MgSO_4$ and concentrated under reduced pressure. Purification of the resulting residue by flash column chromatography (MeOH:CH_2Cl_2:NH_3 (aq.) 4:96:1-7:93:1, R_f (MeOH:CH_2Cl_2:NH_3 (aq.) 8:92:1) = 0.6) gave amine **2** (125 mg, 0.257 mmol, 52% over 2 steps) as an orange solid).

Data for 2: ^{1}H NMR (600 MHz, *d₄*-MeOH): δ (ppm) = 7.87 (d, *J* = 8.6 Hz, 2H), 7.78 (dd, *J* = 10.5, 8.4 Hz, 4H), 7.35 (d, *J* = 8.1 Hz, 2H), 6.88 (t, *J* = 7.8 Hz, 1H), 6.55 (dd, *J* = 7.8, 3.5 Hz, 2H), 3.44 (s, 2H), 2.95 (dtd, *J* = 13.7, 7.3, 6.2, 3.5 Hz, 2H), 2.82 (s, 5H), 2.69 (dd, *J* = 15.7, 11.4 Hz, 1H), 2.63 – 2.55 (m, 2H), 2.49 (ddd, *J* = 17.6, 12.7, 6.2 Hz, 1H), 2.14 – 2.02 (m, 1H), 1.57 – 1.44 (m, 3H), 0.91 (t, *J* = 7.3 Hz, 3H). **^{13}C NMR (100 MHz, d_4-MeOH):** δ (ppm) = 173.7, 155.9, 152.4, 150.1, 145.5, 142.4, 138.6, 130.7, 127.2, 124.7, 124.3, 123.8, 121.6, 120.9, 112.6, 58.24, 53.7, 53.6, 45.7, 35.9, 33.3, 26.9, 24.8, 22.7, 12.3. **IR (ATR):** ν_{max} (cm^{-1}) = 3224 (w), 2918 (w), 1666 (s), 1584 (s), 1524 (vs), 1463 (s). 1404 (m), 1301 (m), 1278 (s), 1153 (m), 1083 (m), 1018 (w), 846 (s), 818 (m), 769 (s). **HRMS (ESI):** calc. for $C_{29}H_{36}N_5O_2^+$ $[M+H]^+$: 486.2864, found: 486.2861.

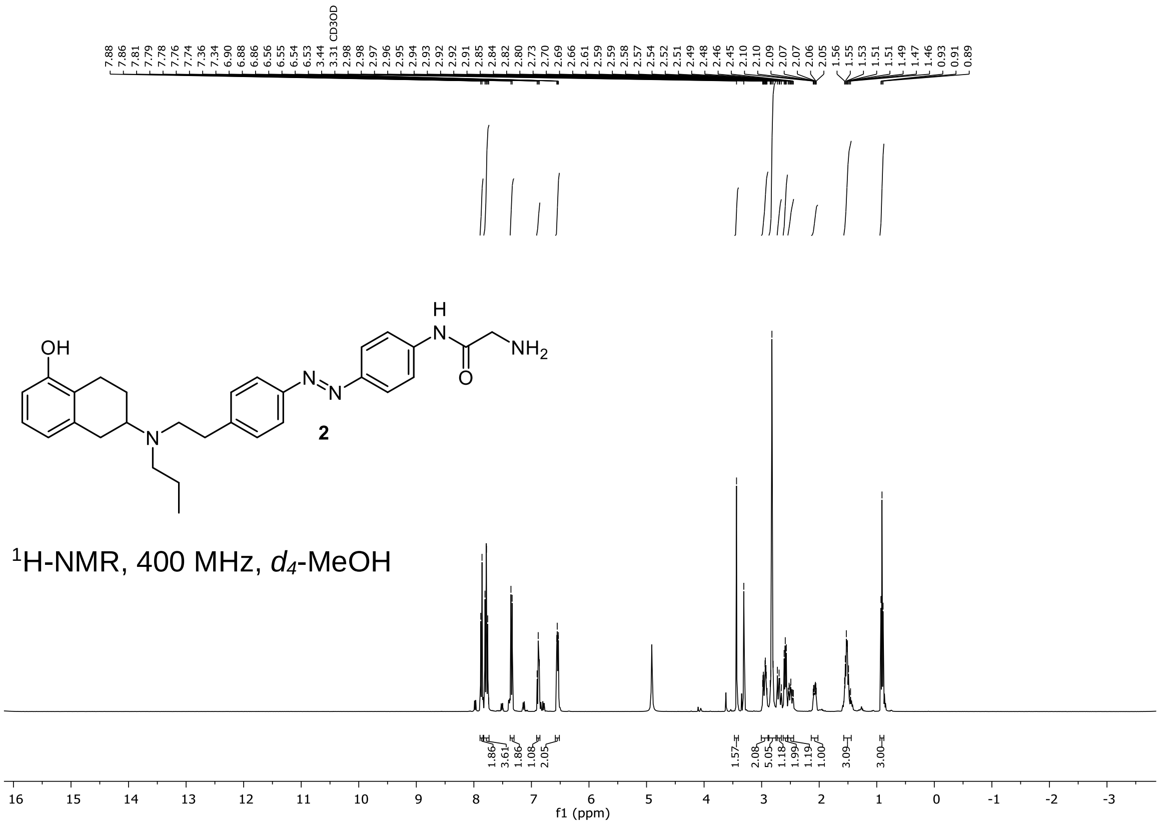
7.88 7.86 7.81 7.79 7.78 7.76 7.74 7.36 7.34 6.90 6.88 6.86 6.56 6.55 6.54 6.53 3.44 3.31 CD3OD 2.98 2.98 2.97 2.96 2.95 2.94 2.93 2.93 2.92 2.92 2.91 2.85 2.84 2.82 2.80 2.73 2.70 2.69 2.66 2.61 2.59 2.59 2.58 2.57 2.54 2.52 2.51 2.49 2.48 2.46 2.45 2.10 2.10 2.09 2.07 2.07 2.06 2.05 1.56 1.55 1.53 1.51 1.51 1.49 1.47 1.46 0.93 0.91 0.89
OH
H
N
NH2
N
N
O
N
2
1H-NMR, 400 MHz, d4-MeOH
1.86 3.61 1.86 1.08 2.05 1.57 2.08 5.05 1.18 1.99 1.19 1.00 3.09 3.00
16 15 14 13 12 11 10 9 8 7 6 5 4 3 2 1 0 -1 -2 -3
f1 (ppm)

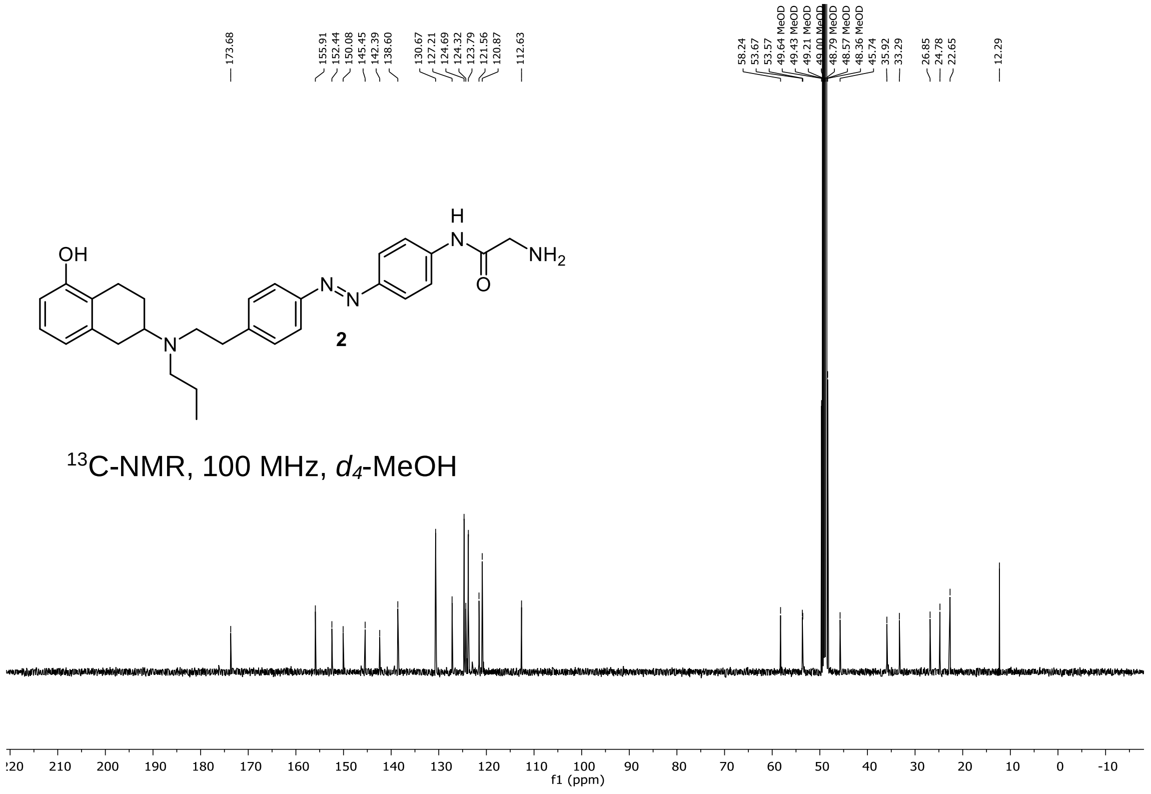
173.68 155.91 152.44 150.08 145.45 142.39 138.60 130.67 127.21 124.69 124.32 123.79 121.56 120.87 112.63 58.24 53.67 53.57 49.64 MeOD 49.43 MeOD 49.21 MeOD 49.00 MeOD 48.79 MeOD 48.57 MeOD 48.36 MeOD 45.74 35.92 33.29 26.85 24.78 22.65 12.29
OH
H
N
NH2
N
N
O
N
2
13C-NMR, 100 MHz, d4-MeOH
220 210 200 190 180 170 160 150 140 130 120 110 100 90 80 70 60 50 40 30 20 10 0 -10
f1 (ppm)

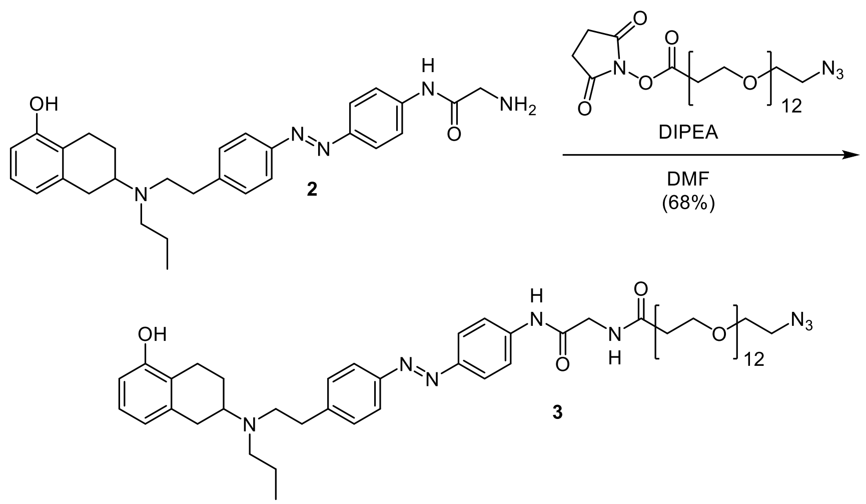

Azide 3: DIPEA (0.03 mL, 0.16 mmol) was added to a stirred solution of amine **2** (39.0 mg, 0.08 mmol) and Azido-$(PEG)_{12}$-NHS-ester (60.0 mg, 0.08 mmol) in DMF (0.5 mL) and the resulting solution was stirred at room temperature overnight. The reaction was diluted with EtOAc and washed with brine. The organic phase was dried over $MgSO_4$ and concentrated under reduced pressure. Purification of the resulting residue by reverse phase column chromatography (MeCN:HCl (aq. 0.1%) (0:100-40:60) gave azide **3** (60 mg, 0.054 mmol, 68%) as an orange solid.

Data for 3: **^{1}H NMR (600 MHz, *d_4*-MeOH):** δ (ppm) = 7.90 (td, *J* = 5.7, 3.0 Hz, 4H), 7.84 – 7.74 (m, 2H), 7.54 (d, *J* = 7.9 Hz, 2H), 6.98 (t, *J* = 7.8 Hz, 1H), 6.67 (dd, *J* = 8.0, 2.1 Hz, 1H), 6.62 (d, *J* = 7.9 Hz, 1H), 4.09 (s, 2H), 3.81 (t, *J* = 5.8 Hz, 3H), 3.76 – 3.49 (m, 48H), 3.48 – 3.32 (m, 4H), 3.31 – 3.05 (m, 8H), 2.75 – 2.51 (m, 3H), 2.40 (d, *J* = 11.8 Hz, 1H), 2.02 – 1.80 (m, 3H), 1.09 (t, *J* = 7.1 Hz, 3H). **^{13}C NMR (100 MHz, *d_4*-MeOH):** δ (ppm) = 174.81, 169.83, 156.08, 153.09, 149.95, 142.64, 140.87, 134.77, 131.03, 127.99, 124.83, 124.24, 123.01, 121.28, 121.09, 113.40, 71.53, 71.48, 71.45, 71.43, 71.38, 71.31, 71.26, 71.10, 68.22, 68.05, 67.60, 61.98, 61.93, 54.10, 53.86, 53.31, 53.02, 51.76, 44.33, 41.89, 37.25, 35.67, 32.20, 32.05, 30.88, 30.76, 25.00, 24.84, 23.76, 20.05, 19.87, 11.53. **IR (ATR):** ν_{max} (cm^{-1}) = 3186 (w), 2972 (w), 2944 (w), 2878 (m), 2819 (m), 2533 (w), 2101 (m), 1946 (w), 1732 (s), 1662 (m), 1593 (m), 1541 (m), 1502 (m), 1464 (m), 1407 (w), 1347 (m), 1301 (m), 1284 (m), 1264 (w), 1247 (m), 1217 (m), 1192 (m), 1086 (s), 1030 (m), 991 8w), 946 (m), 849 (m), 810 (w), 774 (m), 734 (w). **HRMS (ESI):** calc. for $C_{56}H_{87}N_8O_{15}^+$ $[M+H]^+$: 1111.6285, found: 1111.6261.

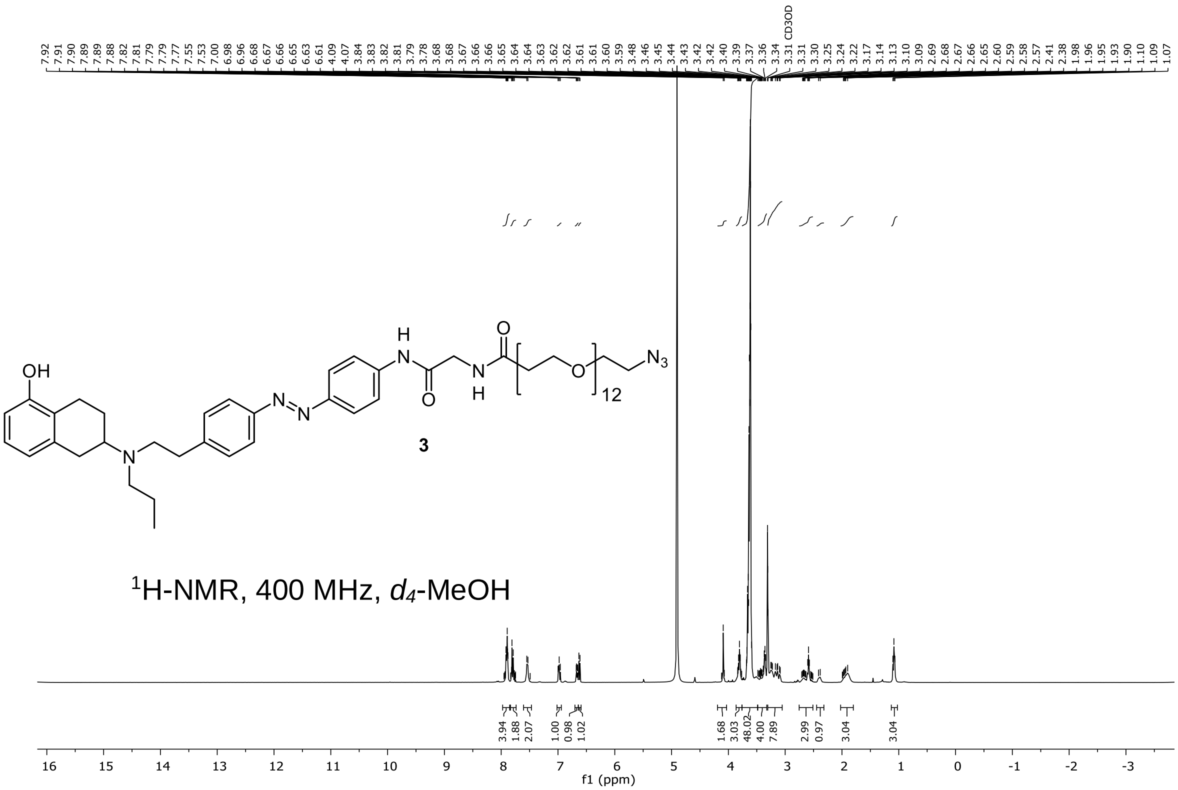

3
^{1}H-NMR, 400 MHz, d_4-MeOH
f1 (ppm)

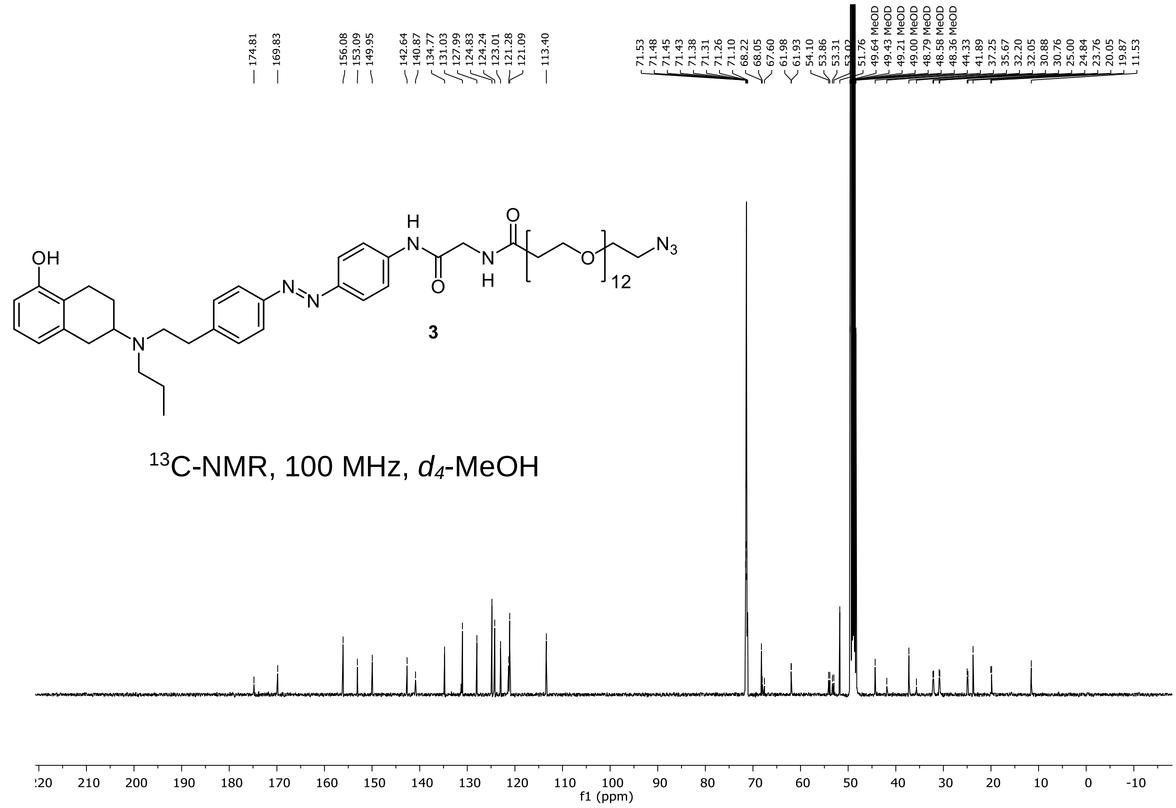

3
^{13}C-NMR, 100 MHz, d_4-MeOH
f1 (ppm)

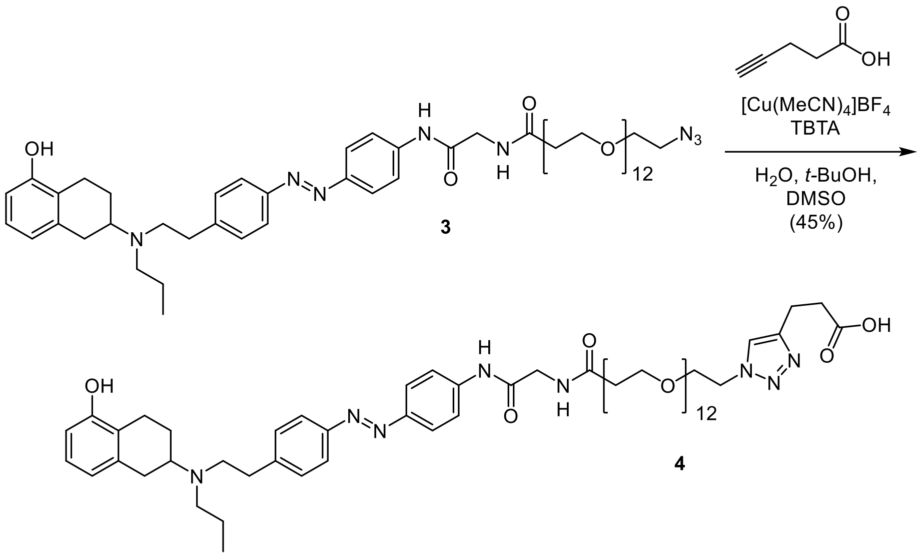

Acid 4: A solution of $[Cu(MeCN)_4]PF_6$ (12.4 mg, 0.033 mmol) in degassed *t*-BuOH/H_2O (2:1 3 mL) was added slowly to a solution of azide **3** (35 mg, 0.031 mmol), 4-pentynoic acid (6.2 mg, 0.063 mmol) and TBTA (17.7 mg, 0.033 mmol) in degassed *t*-BuOH/H_2O (1:1, 3 mL) and degassed DMSO (1 mL) at 0 °C and the mixture was stirred at room temperature for 24 h. The reaction was diluted with EtOAc and washed with brine. The organic phase was dried over $MgSO_4$ and concentrated under reduced pressure. Purification of the resulting residue by reverse phase column chromatography (MeCN:HCl (aq. 0.1%), 0:100-40:60) gave acid **4** (17 mg, 0.014 mmol, 45%) as an orange solid.

Data for 4: ^{1}H NMR (600 MHz, *d$_4$*-MeOH): δ (ppm) = 8.50 (s, 1H), 7.91 (td, *J* = 6.7, 3.5 Hz, 4H), 7.84 – 7.76 (m, 2H), 7.59 – 7.54 (m, 2H), 7.00 (t, *J* = 7.8 Hz, 1H), 6.68 (dt, *J* = 7.6, 1.7 Hz, 1H), 6.64 (d, *J* = 7.9 Hz, 1H), 4.83 – 4.73 (m, 2H), 4.11 (s, 2H), 3.98 (dd, *J* = 5.5, 4.2 Hz, 2H), 3.82 (t, *J* = 5.9 Hz, 3H), 3.78 – 3.34 (m, 46H), 3.27 (dt, *J* = 12.5, 4.5 Hz, 3H), 3.23 – 3.06 (m, 5H), 2.82 (t, *J* = 6.9 Hz, 2H), 2.76 – 2.64 (m, 1H), 2.61 (t, *J* = 5.9 Hz, 2H), 2.42 (t, *J* = 8.0 Hz, 1H), 2.01 – 1.87 (m, 3H), 1.13 – 1.07 (m, 3H). **^{13}C NMR (100 MHz, *d$_4$*-MeOH):** δ (ppm) = 174.8, 173.8, 169.9, 156.2, 153.2, 150.0, 142.7, 140.9, 140.9, 134.8, 130.9, 128.4, 128.0, 124.8, 124.2, 123.0, 121.3, 121.1, 113.4, 71.5, 71.5, 71.4, 71.4, 71.3, 69.4, 68.2, 62.0, 62.0, 54.1, 54.0, 53.7, 53.2, 52.9, 44.3, 37.3, 32.9, 32.1, 32.0, 30.8, 30.7, 25.0, 24.8, 23.7, 20.2, 20.0, 19.8, 11.4, 11.4. **IR (ATR):** ν_{max} (cm^{-1}) = 3230 (br), 2878 (m), 1733 (m), 1669 (m), 1599 (m), 1543 (m), 1503 (m), 1465 (m), 1349 8m), 1300 (m), 1281 (m), 1251 (m), 1090 (vs), 950 (w), 852 (m), 780 (w). **HRMS (ESI):** calc. for $C_{61}H_{94}N_8O_{17}^+$ $[M+H]^{2+}$: 605.3363, found: 605.3363.

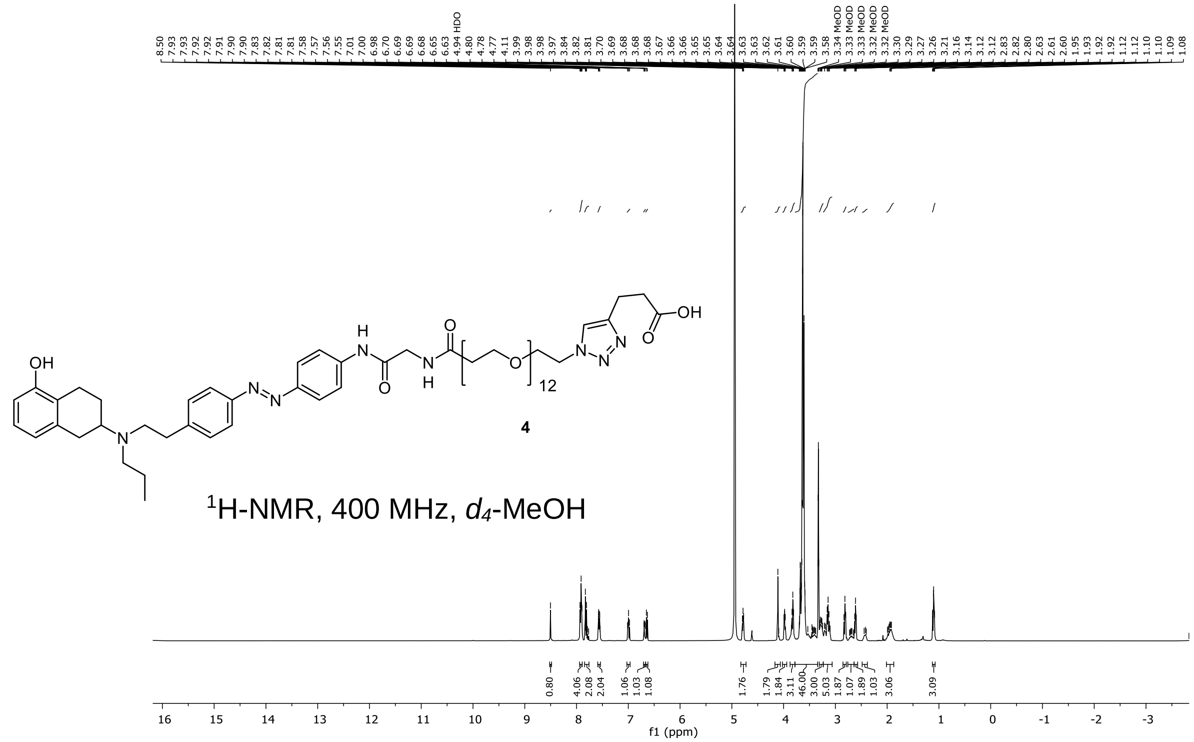

OH
N
N
N
H
N
O
N
H
O
O
12
N
N
N
OH
O
4
1H-NMR, 400 MHz, d4-MeOH
f1 (ppm)

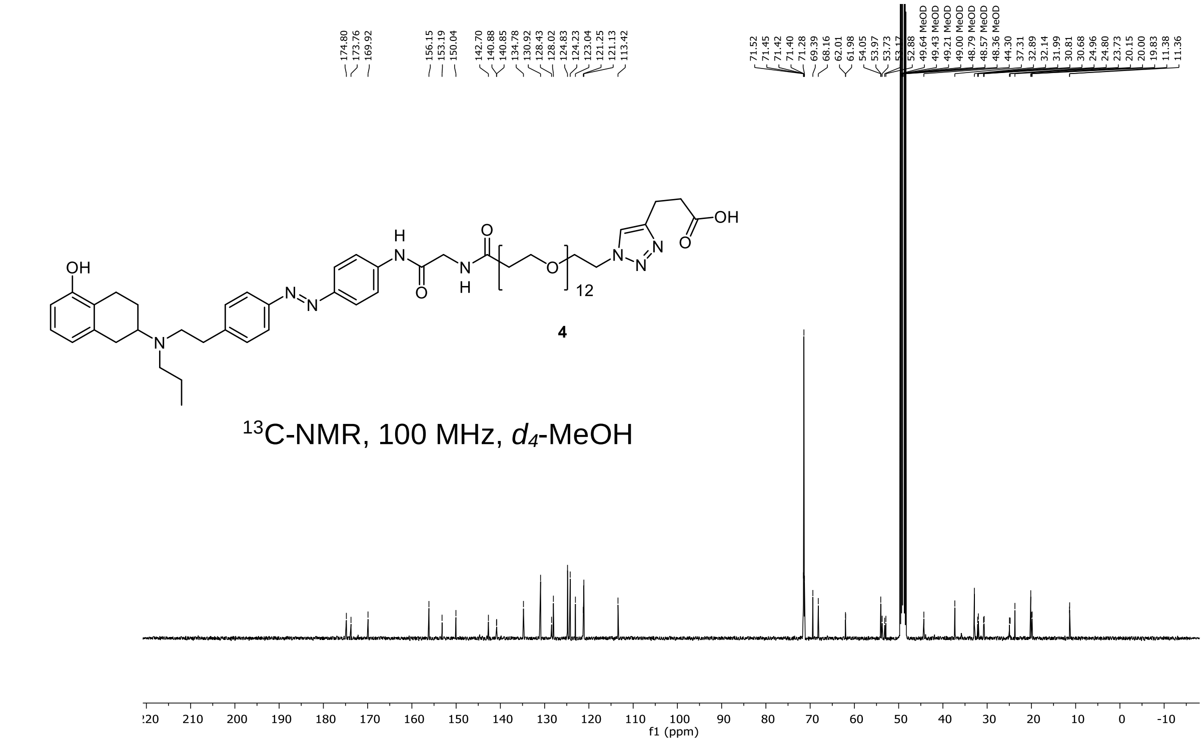

OH
N
N
N
H
N
O
N
H
O
O
12
N
N
N
OH
O
4
13C-NMR, 100 MHz, d4-MeOH
f1 (ppm)

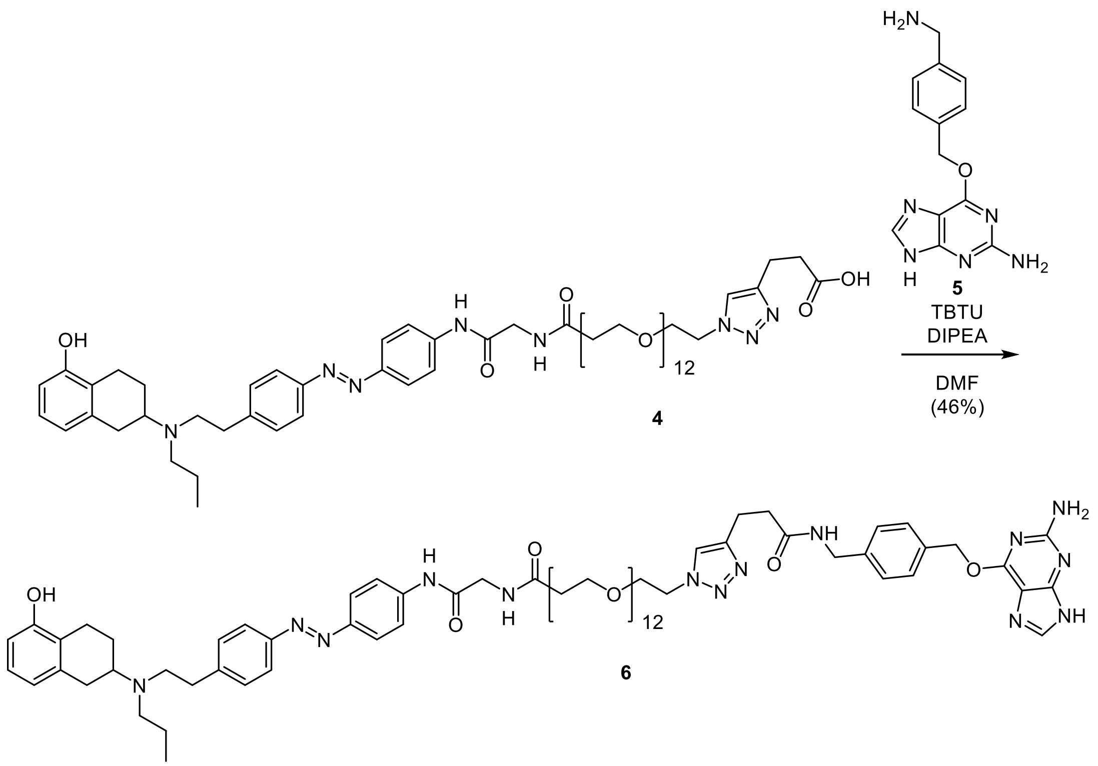

P-D1*ago* (6): DIPEA (0.02 mL, 115 µmol) was added to a solution of acid **4** (9.0 mg, 7.40 µmol), amine **5**[1] (4.0 mg, 14.9 µmol) and TBTU (5.2 mg, 16.0 µmol) in DMF (0.7 mL) and the mixture was stirred at room temperature overnight. Purification by reverse phase HPLC (MeCN:H_2O:HCOOH 10:90:0.1-100:0:0.1) gave **P-D1*ago*** (**6**) (5.0 mg, 3.40 µmol, 46%) as an orange solid.

Data for 6: ^{1}H NMR (600 MHz, *d$_4$*-MeOH): δ (ppm) = 8.50 (s, 2H), 7.88 (ddt, *J* = 9.1, 6.8, 2.5 Hz, 5H), 7.83 – 7.75 (m, 2H), 7.69 (s, 1H), 7.52 – 7.44 (m, 4H), 7.23 – 7.16 (m, 2H), 6.96 (t, *J* = 7.8 Hz, 1H), 6.66 – 6.59 (m, 2H), 5.53 (s, 2H), 4.44 (dd, *J* = 5.5, 4.5 Hz, 2H), 4.33 (s, 2H), 4.08 (s, 2H), 3.81 – 3.74 (m, 4H), 3.67 – 3.50 (m, 48H), 3.43 (dd, *J* = 10.3, 6.5 Hz, 2H), 3.13 (dddd, *J* = 24.0, 16.8, 9.8, 4.5 Hz, 6H), 3.00 (t, *J* = 7.3 Hz, 2H), 2.59 (td, *J* = 6.6, 5.9, 4.2 Hz, 4H), 2.38 – 2.28 (m, 1H), 1.86 – 1.79 (m, 2H), 1.05 (t, *J* = 7.3 Hz, 3H). **^{13}C NMR (100 MHz, *d$_4$*-MeOH):** δ (ppm) = 174.8, 174.3, 170.1, 170.0, 169.9, 161.7, 161.4, 156.1, 153.1, 150.1, 147.4, 142.7, 141.6, 140.1, 137.0, 135.4, 130.8, 129.7, 128.7, 127.9, 124.8, 124.4, 124.2, 123.3, 121.3, 121.2, 113.3, 71.4, 71.4, 71.3, 70.3, 68.5, 68.2, 61.3, 53.8, 53.1, 51.3, 44.3, 43.8, 37.4, 36.4, 32.8, 31.2, 25.2, 23.9, 22.6, 20.4, 11.5. **IR (ATR):** ν_{max} (cm^{-1}) = 2870 (s), 2361 (m), 1586 (vs), 1506 (w), 1465 (s), 1350 (m), 1280 (w), 1106 (s), 949 (w), 851 (w), 668 (w). **HRMS (ESI):** calc. for $C_{74}H_{106}N_{14}O_{17}^+$ $[M+2H]^{2+}$: 731.3916, found: 731.3925.

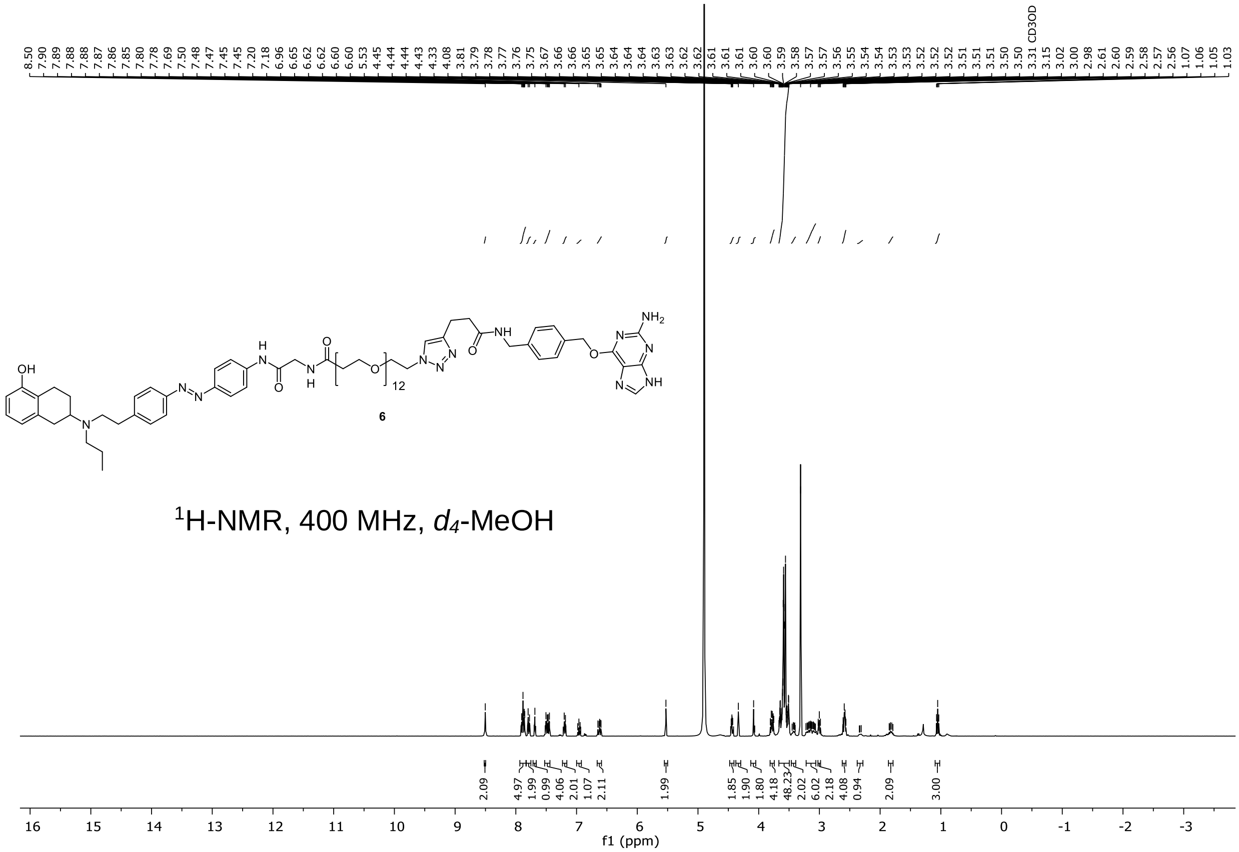

^{1}H-NMR, 400 MHz, d_4-MeOH

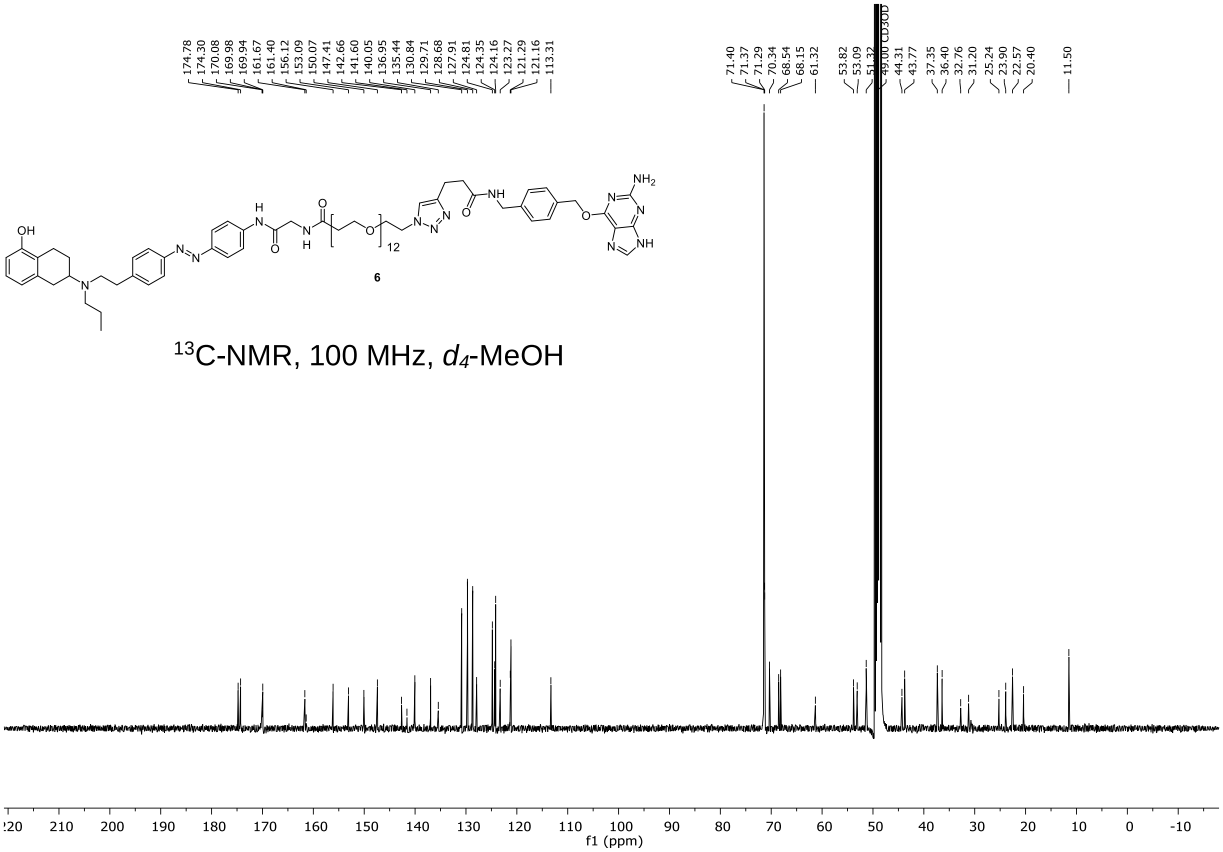

^{13}C-NMR, 100 MHz, d_4-MeOH

Supplementary Figures

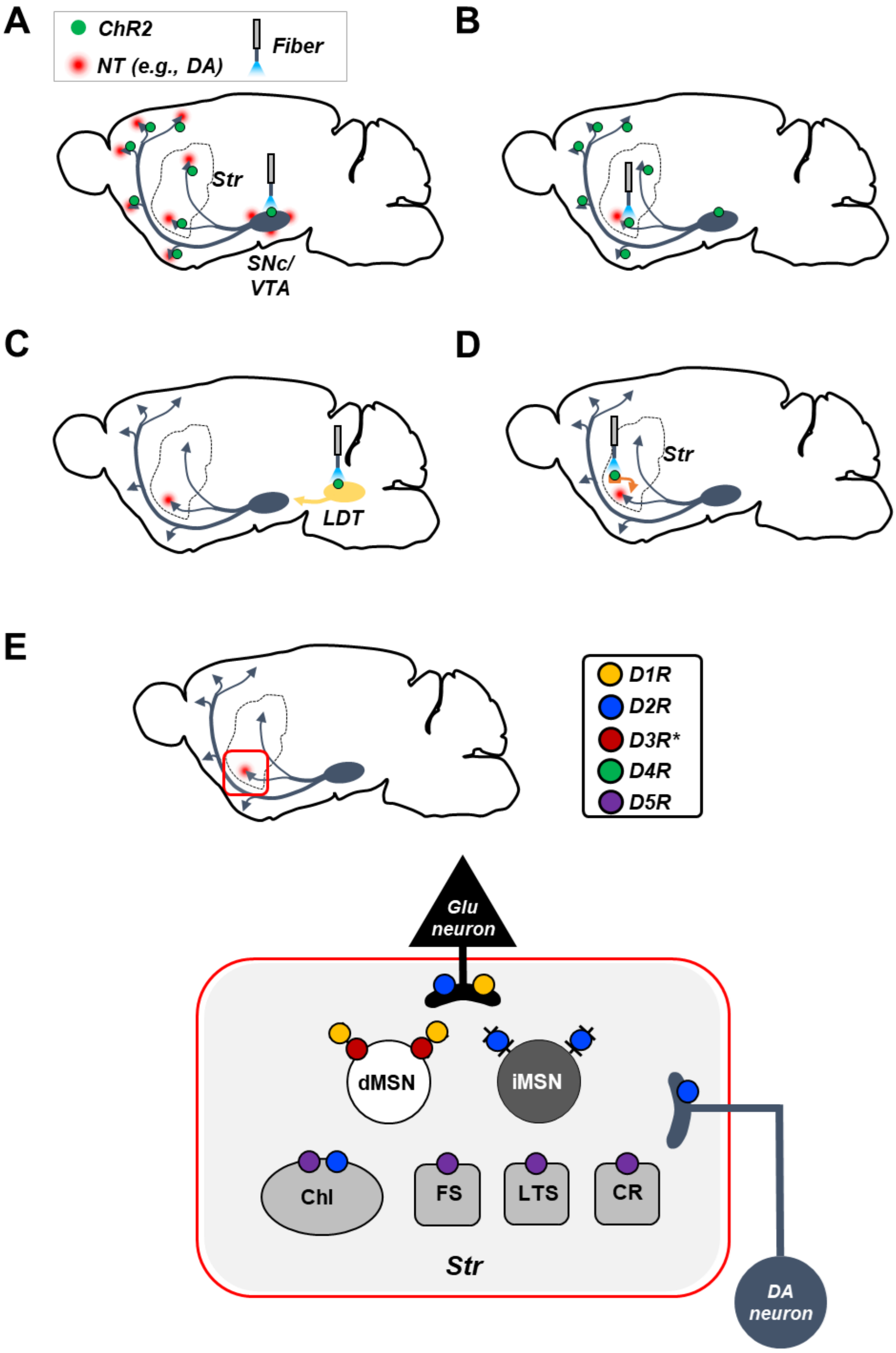

Supplementary Figure 1. Excitatory opsins have multiple, diverse, and in some cases opposing effects on neural circuits. Channelrhodopsin-2 (ChR2), a light-gated excitatory opsin, is widely used to map neural circuits[2]. **(A)** ChR2 is typically activated in the somata of genetically-defined neurons, resulting in enhanced firing and neurotransmitter (NT) release. This can increase synaptic transmission in multiple, functionally distinct projection areas. For example, ChR2 has been used to control DA neurons in the SNc[3] and VTA[4], each of which innervates diverse brain areas[5, 6]. Several approaches have been implemented to increase target specificity (B-D): **(B)** ChR2 can be activated in presynaptic axon terminals in a selected brain area, as has been done in specific subregions of the striatum[7]*.* **(C)** ChR2 can be activated in upstream neurons that innervate specific DA neurons, as has been done with laterodorsal tegmentum (LDT) neurons (yellow), which innervate a subpopulation of VTA neurons that project to the ventral striatum[8]. **(D)** ChR2 can be activated in neurons that form axo-axonal synapses onto the terminals of other neurons in a brain area, as do (i) striatal cholinergic interneurons (ChIs; orange), which release acetylcholine and activate nicotinic acetylcholine receptors on DA terminals to enhance DA release[9] or (ii) cortical neurons, which release glutamate and activate Group I mGluRs on DA axons to suppress DA release[10]. However, ChIs and cortical neurons also connect to other striatal neurons and excitation of axons could lead to antidromic action potentials that evoke release in axon collaterals that project elsewhere. **(E)** When ChR2 activation evokes DA release, multiple DA receptor subtypes can be activated simultaneously, via direct synapses[11] and volume transmission[12], including sometimes a $G_{s/olf}$-coupled D1-like receptor (D1, D5) and an opposing $G_{i/o/z}$-coupled D2-like receptor (D2, D3, D4) in the same cell. DA activates receptors in each of the cell types of the striatum, in presynaptic D2 autoreceptors that inhibit release from DA neuron terminals[13], and D1 and D2 heteroreceptors in the terminals of glutamatergic inputs to the striatum[14, 15]. DA neurons co-release glutamate (Glu)[7] and/or GABA[16], which activate postsynaptic ligand-gated ion channels (LGICs) and G protein-coupled receptors (GPCRs) (*not shown*). *D3R is expressed in the ventral striatum but not the dorsal striatum[17]. dMSN = direct-pathway medium spiny neurons, iMSN = indirect-pathway medium spiny neurons, ChI = cholinergic interneurons, FS = fast-spiking interneurons, LTS = low-threshold spiking interneurons, CR = calretinin interneurons.

			Functional effect			Spatial precision			Temporal precision	
			Targets intact endogenous receptors	*Gain of function*	*Loss of function*	*Receptor-specific*	*Region-specific*	*Cell type-specific*	*Fast (<min)*	*Slow (>min)*
**Pharmacology** (*agonists, antagonists, allosteric modulators)*	**Systemic pharmacology**	**injection**	✓	✓	✓	✓*				✓
	**Region-specific pharmacology**	**infusion**	✓	✓	✓	✓*	✓			✓
	**Photopharmacology (*caged and photochromic ligands*)**	**Infusion + light**	✓	✓	✓	✓*	✓		✓	✓
**Genetics**	**Global knockout/ knockdown**	**embryonic modification**			✓	✓				✓
	**Conditional knockout/ knockdown**	**embryonic modification**			✓	✓	✓	✓		✓
	**Overexpression**	**embryonic modification/ virus**		✓		✓	✓	✓		✓
**Engineered receptors**	**Chemogenetics (*PSAMs, DREADDs/RASSLs*)**	**virus + injection**		✓			✓	✓		✓
	**Optogenetics (*opto-XRs*)**	**virus + light**		✓		✓#	✓	✓	✓	
	**Tethered photopharmacology (*PTLs, PORTLs*)**	**virus + infusion + light**		✓	✓	✓#	✓	✓	✓	✓
**Membrane-anchored ligands**	**t-toxins**	**virus**	✓	✓	✓	✓*	✓	✓		✓
	**DARTs**	**virus + infusion**	✓	✓	✓	✓*	✓	✓		✓
	**LumiToxins**	**virus + light**	✓	✓	✓	✓*	✓	✓		✓

* Receptor specificity is determined by the inherent selectivity of the pharmacophore.

# opto-XRs and PTL- or PORTL-gated receptors are variants of wildtype endogenous GPCRs. However, they are not completely identical and thus may not accurately reflect the activity of endogenous receptors.

Supplementary Figure 2. Comparison of existing methods for targeting neuronal receptors *in vivo.* Ligand-gated ion channels (LGICs) and G protein-coupled receptors (GPCRs) can be controlled through a variety of means. Conventional pharmacology (agonists, antagonists, and allosteric modulators) is used to increase or decrease endogenous receptor activity. However, these ligands are freely diffusible and thus not cell type selective and difficult to constrain to the site of infusion. Furthermore, infusion kinetics *in vivo* are slow (minutes to longer) relative to the millisecond to seconds timescale of physiological receptor activation[18-20]. Faster kinetics can be achieved with photopharmacology, where free caged or photoswitchable ligands can be converted to the active state in milliseconds[21]. However, free photopharmacologic ligands cannot target specific cell types. Genetic modifications (knockout, knockdown, overexpression) can target a specific brain area and cell type, but the effect is chronic and could result in compensation. Chemogenetic LGICs (PSAMs)[22] and GPCRs (DREADDs/RASSLs)[23] combine genetic targeting with timed activation by orthogonal synthetic ligands. They are not available for most neuronal receptors and are used to override native signaling. They activate and deactivate with the slow kinetics of conventional pharmacology. Light-sensitive GPCRs made by fusion of a transmitter-gated GPCR with rhodopsin (opto-XRs) are rapidly activated, but deactivate slowly, recover incompletely[24], and do not recapitulate all of the functions of the native receptor.

Two methods of photo-pharmacology control native receptors in a way that combines genetic targeting and tight spatio-temporal control of native signaling proteins: a) Photoswitchable Tethered Ligands (PTLs) that attach covalently to a receptor via an engineered cysteine and b) Photoswitchable Orthogonal Remotely Tethered Ligands (PORTLs) that attach covalently to an orthogonal anchoring domain (e.g., SNAP-tag) that is fused to the full-length wildtype receptor[25-27]. These engineered receptors must be overexpressed or knocked in. Membrane-anchored ligands selectively bind a target receptor as a result of a random encounter at the cell surface in combination with their inherent binding affinity[28]. They can be incorporated with peptidic ligands (t-toxins) [28] or chemical ligands (DARTs)[29]. However, once applied, they cannot be turned off until removed by the cell, a process that can at best take days[29]. The light-sensitive domain LOV was recently incorporated into t-toxins, making them acutely activatable (LumiToxins) but takes minutes to turn off and has poor efficacy[30]. Lumitoxins cannot be incorporated with chemical ligands like those that bind many physiologically and clinically relevant neuronal receptors.

The membrane-anchored PORTL (MP) approach used in this study combines the advantages of DARTs with the spatio-temporal precision of photo-pharmacology to control unmodified native receptors.

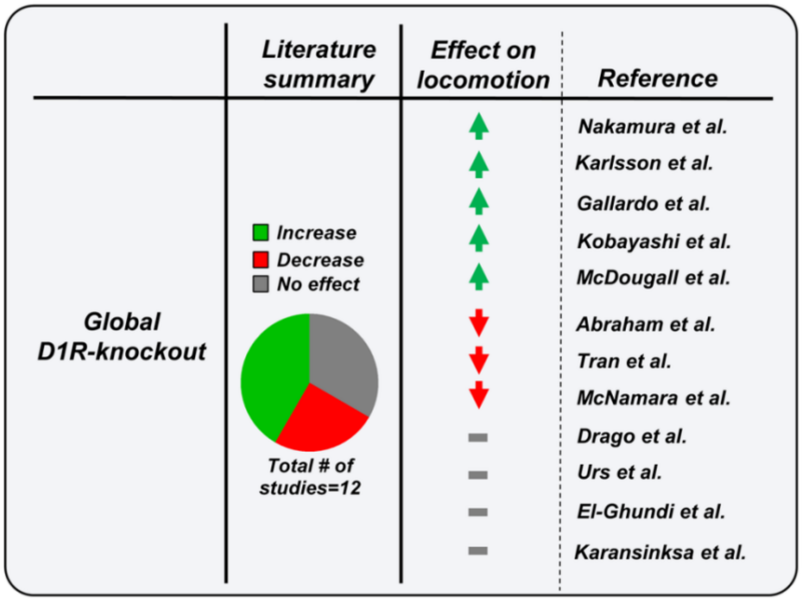

Supplementary Figure 3. Knockout studies provide an inconsistent view of the role of D1R in the movement. Global D1R knockout has conflicting effects on locomotion with locomotion increase (upward green arrow)[31-35], decrease (downward red arrow)[36-38], or no effect (grey bar)[39-42] observed.

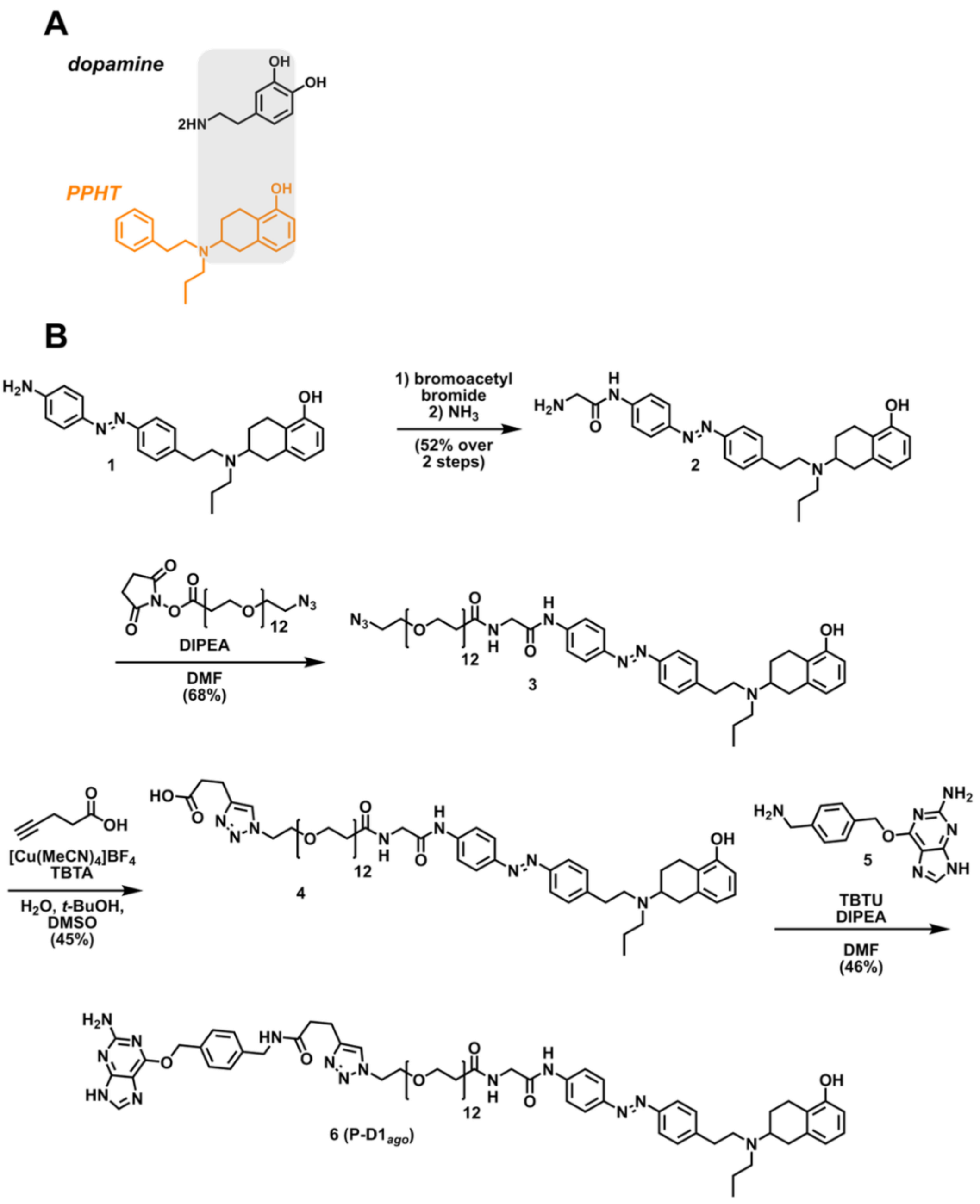

Supplementary Figure 4. Chemical synthesis of P-D1$_{ago}$. (A) Alignment of dopamine (DA) with the synthetic DAR agonist PPHT, a rigidified aminotetralin analogue of DA that contains only one hydroxyl as well as N-propyl and N-phenylethyl groups that enhance metabolic stability *in vivo* and affinity toward DARs[43-48]. **(B)** Synthesis scheme for P-D1$_{ago}$.

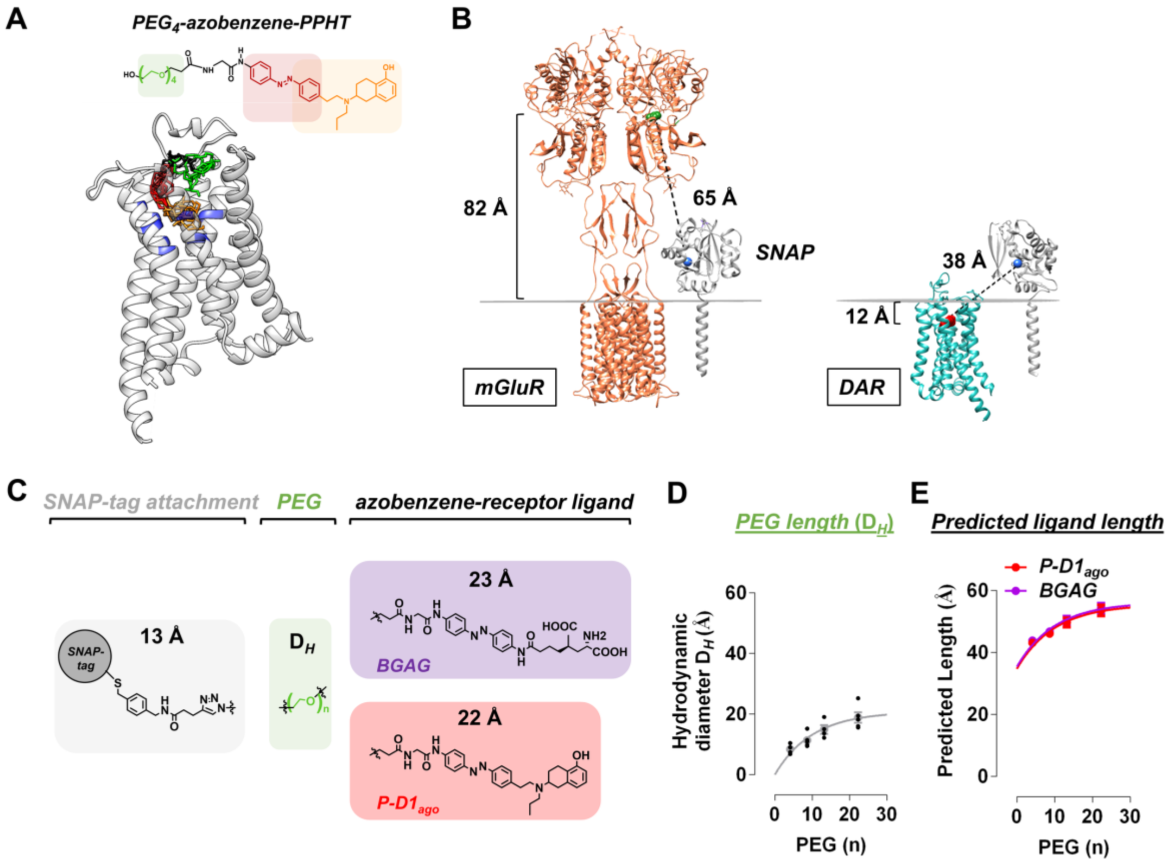

Supplementary Figure 5. Design of P-D1$_{ago}$. (A) P-D1$_{ago}$ consists of azobenzene-PPHT conjugated to benzylguanine via a polyethylene glycol (PEG) chemical linker. Docking studies were used to gain insight into where the PEG linker should be attached to azobenzene. Multiple poses of a fragment of *trans*-P-D1$_{ago}$ (PEG$_4$-azobenzene-PPHT) are shown docked in the dopamine (DA) binding site of a D1R homology model based on a crystal structure of β2AR (pdb: 2RH1). The PPHT moiety (orange) binds D1R within the DA binding site (backbone residue in blue). Azobenzene (maroon) and the PEG linker (green) reside within the pore that leads to the DA binding site, and the PEG linker extends out of the receptor. **(B)** To optimize the length of the PEG linker in P-D1$_{ago}$, we compared it to an MP that we developed previously for a metabotropic glutamate receptor (mGluR), benzylguanine-azobenzene-glutamate (BGAG). BGAG requires a long PEG linker (28 repeats works, but zero and 12 repeats do not) to reach from the SNAP-tag adjacent to the cell surface (the membrane anchor M) to the clamshell ligand binding site situated well above the plasma membrane, as typical in Family C GPCRs[49]. Longer linkers would mean the ligand explores a wider three-dimensional space and have a lower effective concentration,

slower on-rate, and lower occupancy in the permissive state. Therefore, MPs have an optimal length that is just long enough to reach. Unlike mGluRs, Family A GPCRs like DARs bind ligand in the upper third of their transmembrane bundle. To provide structural insight into the length dependence of photoswitching, we estimated the minimal distance between the benzylguanine binding site in the M and the ligand binding site in either mGluR5 (left; pdb: 6n51)[50] or the D1R homology model (right). A model of the M was made by placing the C-terminus of SNAP-tag (pdb: 3L00) flush with the N-terminus of a single-pass transmembrane segment (pdb: 2K1A)[51]. The M was then positioned adjacent to mGluR5 or D1R. The benzylguanine binding site in the M (blue circle) was oriented facing towards the receptor ligand binding site. L-quisqualate (green) is shown bound to the ligand binding site of mGluR5. DA (red) is docked in the D1R homology model. **(C)** We next estimated the lengths of the BGAG and P-$D1_{ago}$. Their chemical structures are shown bound to SNAP-tag. Each photoswitch is composed of (i) benzylguanine bound covalently to the reactive Cys145 of SNAP-tag, (ii) a PEG linker with a hydrodynamic diameter (D_H) that depends on the number of ethylene glycol repeats (n), and (iii) azobenzene conjugated to a receptor ligand (glutamate in BGAG and PPHT in P-$D1_{ago}$). **(D)** The hydrodynamic diameter (D_H) of PEG is shown as a function of increasing ethylene glycol repeats. D_H values were taken from multiple studies[52-56] and averaged. In each study, D_H was measured at room temperature in water. Because D_H is not sensitive to temperature[57], these values should be valid for experiments performed at room temperature or *in vivo*. n = 4 data points for all PEG_4 and PEG_{13}, and n = 5 data points for PEG_8 and PEG_{22}. Error bars indicate S.E.M. **(E)** The estimated length of BGAG and P-$D1_{ago}$ are shown as a function of increasing ethylene glycol repeats using published PEG D_H values (D). These results indicate that P-$D1_{ago}$ with 12-PEG repeats (~50 Å) is sufficiently long enough to span from the SNAP-tag in the M to the DA binding site in D1R (38 Å) (B). A BGAG with a long PEG linker (28 repeats; ~55 Å) would not effectively reach from the M to the mGluR ligand binding site (65 Å) (B), consistent with our previous results[49] that BGAG with 28-PEG requires a “lift” peptide to raise the SNAP above the plasma membrane. n = 4 data points for all PEG_4 and PEG_{13}, and n = 5 data points for PEG_8 and PEG_{22}. Error bars indicate S.E.M.

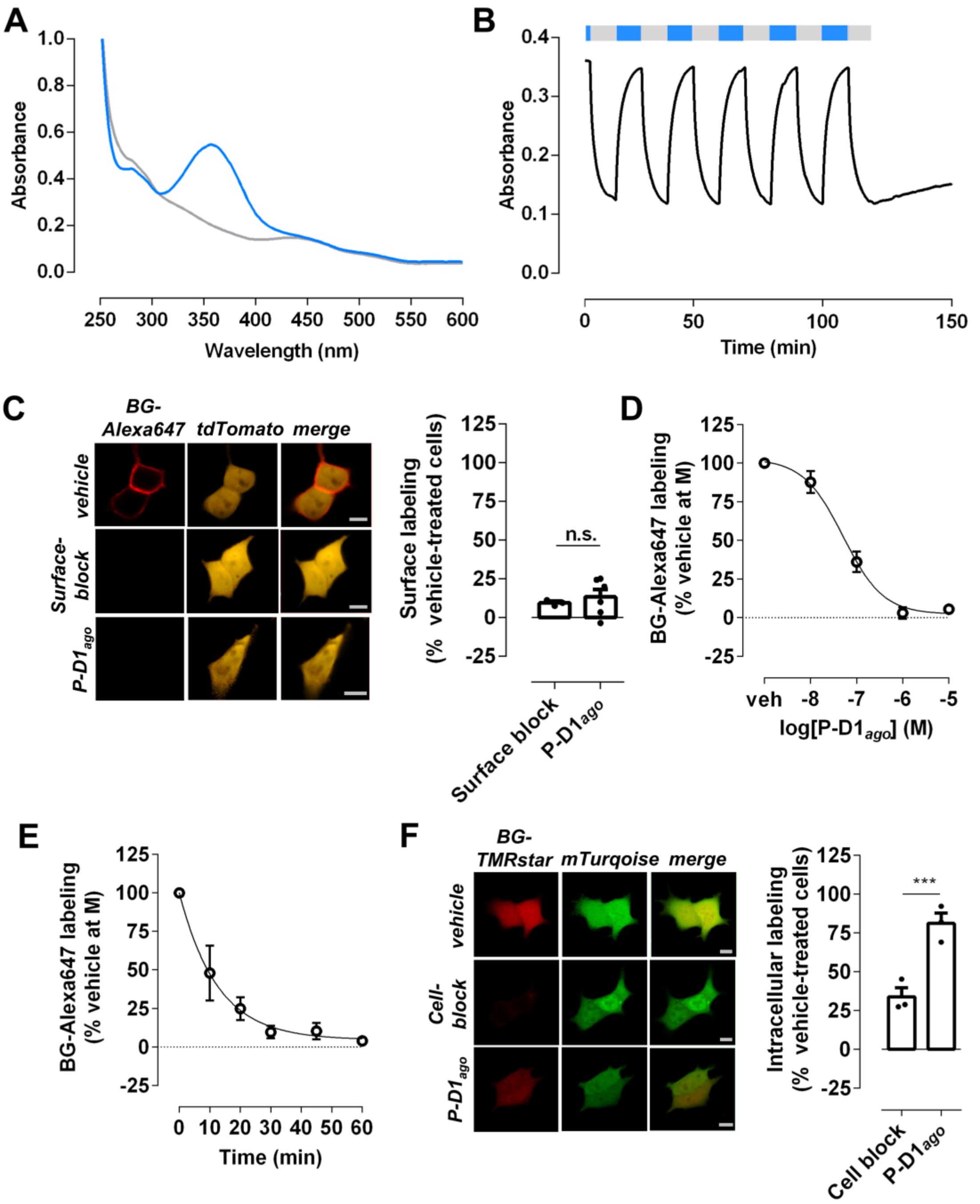

Supplementary Figure 6. Photophysical properties of P-D1$_{ago}$. (A) Absorbance spectra of P-D1$_{ago}$ under blue light (460 nm; blue) and UV light (370 nm; grey). P-D1$_{ago}$ (30 µM) in 10% DMSO. **(B)** P-D1$_{ago}$ repeatedly switches from *trans* to *cis* with UV light and back to *trans* with blue light. P-D1$_{ago}$ slowly reverts from *cis* to *trans* state in the dark following the last UV light pulse (τ = 311 ± 32 m, n = 2). **(C)** P-D1$_{ago}$ conjugation is efficient, as seen by block of conjugation by the membrane impermeant dye SNAP-Surface Alexa Fluor 647 (BG-Alexa647) to the membrane-

anchor (M). The efficiency is similar to that of the impermeant benzylguanine analog, surface block. Cells incubated for 1 hour with vehicle (left upper panels), 1 µM surface block (left middle panels), or 1 µM P-D1$_{ago}$ (left lower panels), and then labeled for 30 minutes with 1.5 µM BG-Alexa647. Cytosolic tdTomato was used as a transfection marker. grey bar = 10 µM. A summary of labeling measured by FACS. Unpaired two-sided t-test, $p = 0.60$. n = 3 for surface block and 6 for P-D1$_{ago}$. **(D)** M labeling as a function of the concentration of P-D1$_{ago}$. The M was incubated for 1 hour with increasing concentrations of P-D1$_{ago}$, and then labeled for 30 minutes with 1.5 µM BG-Alexa647. Labeling was measured by flow cytometry. n = 4 replicates per condition except 1 µM P-D1$_{ago}$, where n = 3. **(E)** Time course of M labeling with P-D1$_{ago}$. The M was incubated for different times with 1 µM P-D1$_{ago}$, and then labeled for 30 minutes with 1.5 µM BG-Alexa647. Labeling was measured by flow cytometry. n = 3 replicates per condition except 60 minutes, where n = 2. **(F)** P-D1$_{ago}$ does not effectively prevent the binding of the membrane permeant dye SNAP-Cell TMR-Star (BG-TMRstar) to intracellular SNAP-tag that is expressed in the cytosol. This contrasts with the commercially available permeant benzylguanine analog, cell block, indicating that P-D1$_{ago}$ is not cell permeable. Cells were incubated for 1 hour with vehicle (left upper panels), 1 µM cell block (left middle panels), or 1 µM P-D1$_{ago}$ (left lower panels) and then labeled for 20 minutes with 1.5 µM BG-TMRstar. Cytosolic mTurquoise was used as a transfection marker. Grey bar = 10 µM. A summary of labeling measured by FACS is shown on the right. Unpaired two-sided t-test, ***$p = 0.006$. n = 3 replicates per condition. Error bars indicate S.E.M.

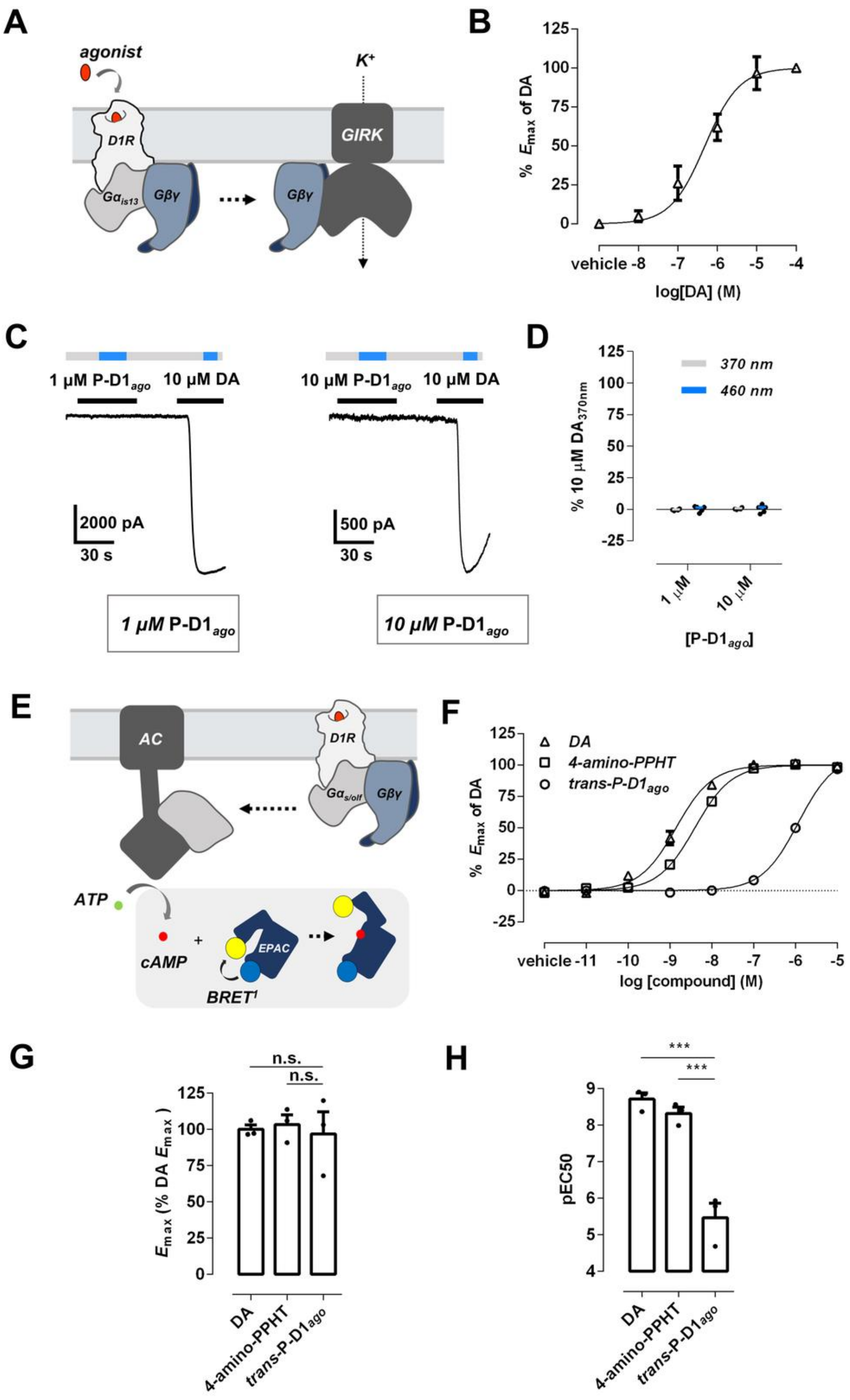
A
agonist
K+
D1R
GIRK
Gαis13
Gβγ
Gβγ
B
125
100
75
50
25
0
% Emax of DA
vehicle -8 -7 -6 -5 -4
log[DA] (M)
C
1 μM P-D1ago
10 μM DA
2000 pA
30 s
10 μM P-D1ago
10 μM DA
500 pA
30 s
1 μM P-D1ago
10 μM P-D1ago
D
% 10 μM DA370nm
370 nm
460 nm
125
100
75
50
25
0
-25
1 μM
10 μM
[P-D1ago]
E
AC
D1R
Gαs/olf
Gβγ
ATP
cAMP
EPAC
BRET1
F
DA
4-amino-PPHT
trans-P-D1ago
125
100
75
50
25
0
-25
% Emax of DA
vehicle -11 -10 -9 -8 -7 -6 -5
log [compound] (M)
G
n.s.
n.s.
Emax (% DA Emax)
125
100
75
50
25
0
DA
4-amino-PPHT
trans-P-D1ago
H
***
***
pEC50
9
8
7
6
5
4
DA
4-amino-PPHT
trans-P-D1ago

Supplementary Figure 7. Functional properties of untethered P-D1$_{ago}$. (A) Schematic representation of a D1R-mediated G protein-coupled inwardly rectifying potassium (GIRK) channel assay in HEK293T cells. Agonist-induced receptor activation results in the recruitment of a heterotrimeric G protein containing the chimeric Gα subunit (Gα$_{is}$) followed by the release of Gβγ, which activates GIRK channels and enhances inward-current. **(B)** Dopamine (DA) dose-dependently activates D1R in the GIRK assay. The potency of DA (pEC50 = 6.3 ± 0.1) is comparable to that observed for endogenous DARs in intact tissue[58]. pEC50 is defined as the negative logarithm of the EC50 in molar units. n = 6 for 10 µM, n = 8 for 10 nM and 100 nM, and n = 9 for 1 µM. **(C)** Representative traces of the effect of untethered P-D1$_{ago}$ on D1R in the absence of the membrane-anchor (M) according to the GIRK assay. Neither 1 µM or 10 µM P-D1$_{ago}$, concentrations that fully label the M (Supplementary Fig. 6d), have any effect on the receptor in either the *trans* (blue light; blue bars) or *cis* (UV light; grey bars) isomeric state. **(D)** Summary of the functional effect of untethered P-D1$_{ago}$ on D1R in the GIRK assay. n = 4 for 370 nm and n = 5 for 460 nm. **(E)** Schematic representation of a highly sensitive D1R-mediated cAMP accumulation assay in HEK293T cells. Agonist-induced D1R activation results in G$_{s/olf}$ recruitment followed by the binding of its Gα subunit to adenylate cyclase (AC), which converts ATP to cAMP. cAMP binds the bioluminescence resonance energy transfer 1 (BRET^1)-based cAMP sensor CAMYEL[59], resulting in a conformational change in its Epac domain that increases distance and decreases BRET^1 between the donor *Renilla* luciferase (Rluc) and the acceptor YFP. **(F)** Representative dose-response curves of DA, 4-amino-PPHT (a parent molecule of P-D1$_{ago}$), or untethered *trans*-P-D1$_{ago}$ induced activation of D1R according to the cAMP accumulation assay. n = 3 replicates per concentration. **(G,H)** Summary of the E_{max} (G) and pEC50 (H) of DA, 4-amino-PPHT, or untethered *trans*-P-D1$_{ago}$ in the cAMP accumulation assay. One-way ANOVA: F = 0.1, Tukey (E_{max}), F = 43.9, Tukey, ***$p < 0.001$ (pEC50) n = 3 experiments, each performed in triplicate per concentration. Error bars indicate S.E.M.

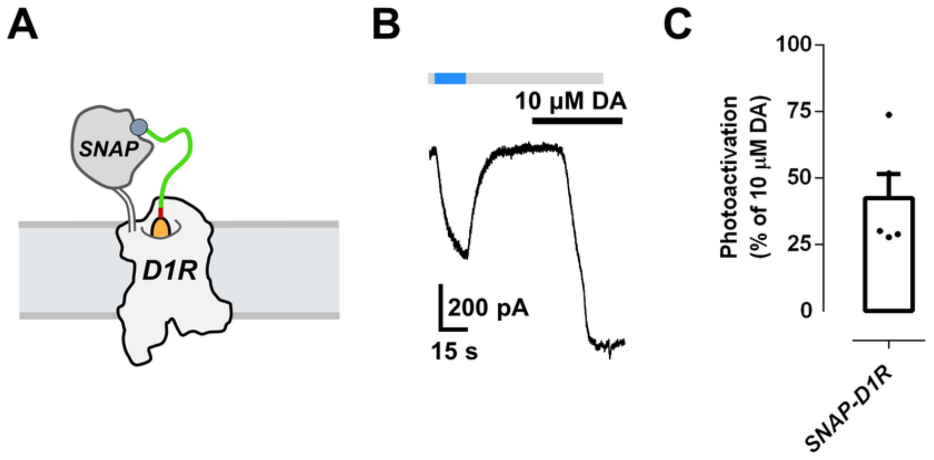

Supplementary Figure 8. Functional characterization of the effect of P-D1$_{ago}$ tethered to SNAP-D1R. (A) Schematic representation of P-D1$_{ago}$ tethered to a SNAP-tag fused directly to the extracellular N-terminus of D1R. **(B)** Representative trace of SNAP-D1R photoactivation by P-D1$_{ago}$ according to the GIRK assay. P-D1$_{ago}$ photoactivates D1R in response to blue light and is deactivated with UV light. **(C)** Summary of SNAP-D1R photoactivation by P-D1$_{ago}$ relative to a saturating concentration of DA (10 µM). n = 5 cells. Error bars indicate S.E.M.

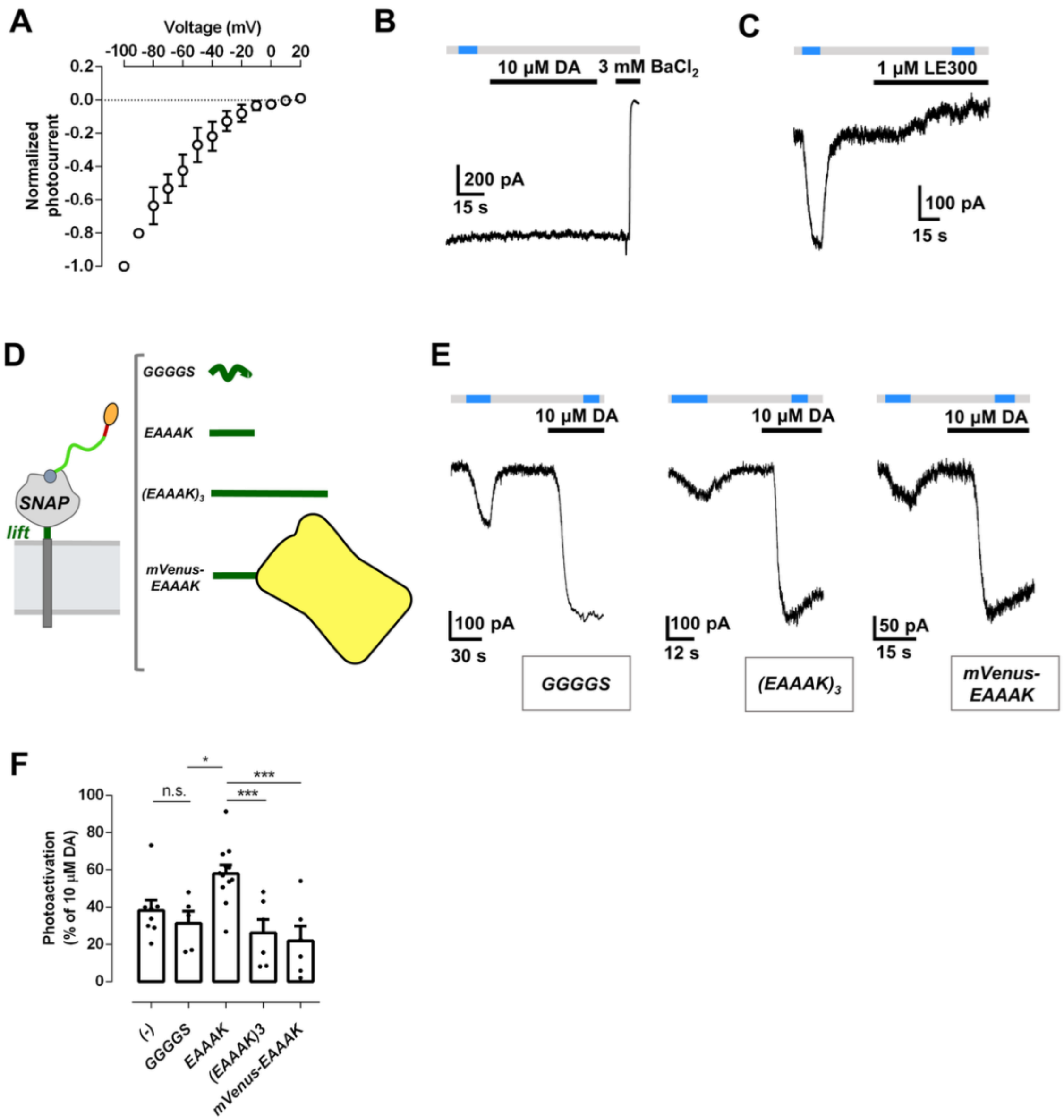

Supplementary Figure 9. Functional characterization of the effect of P-D1$_{ago}$ tethered to M or its variants on D1R. (A) Inward-current in response to D1R photoactivation with P-D1$_{ago}$ tethered to the SNAP-tag containing membrane anchor (M) as a function of voltage (photo-IV curve) in HEK293T cells. Switching from UV light to blue light has a negligible effect on inward-current at positive voltages but decreases exponentially as voltage becomes more negative, consistent with the current-voltage relationship associated with activation and opening of GIRK channels[60]. n = 3 cells per voltage. **(B)** Switching from UV light to blue light has no effect on the basal current of 1 µM P-D1$_{ago}$ labeled HEK293T cells expressing GIRK channels but not D1R (1.3 ± 1.0% of 3 mM $BaCl_2$, a GIRK channel blocker. Paired two-sided t-test, p = 0.002. n = 5 cells).

(C) Photoactivation of D1R by P-$D1_{ago}$ tethered to the M is abolished in the presence of the D1R antagonist LE300 (3.3 ± 3.3% of photocurrent in the absence of LE300. n = 5 cells per condition). The decrease in inward-current in response to LE300 results from its actions as an inverse agonist that reduces constitutive receptor activity[61]. **(D)** Schematic representation of M analogs with lift peptides of different lengths and physical characteristics. **(E)** Representative traces of D1R photoactivation with P-$D1_{ago}$ tethered to M variants with different lift peptides. **(F)** Summary of photoactivation of D1R by P-$D1_{ago}$ with M variants with different lift peptides. One-way ANOVA, F = 17.1, Tukey, *$p < 0.05$, ***$p < 0.001$. n = 5 cells for GGGGS, n = 6 cells for $(EAAAK)_3$ and mVenus-EAAAK, n = 8 cells for M with no linker, and n = 12 cells for EAAAK. Error bars indicate S.E.M.

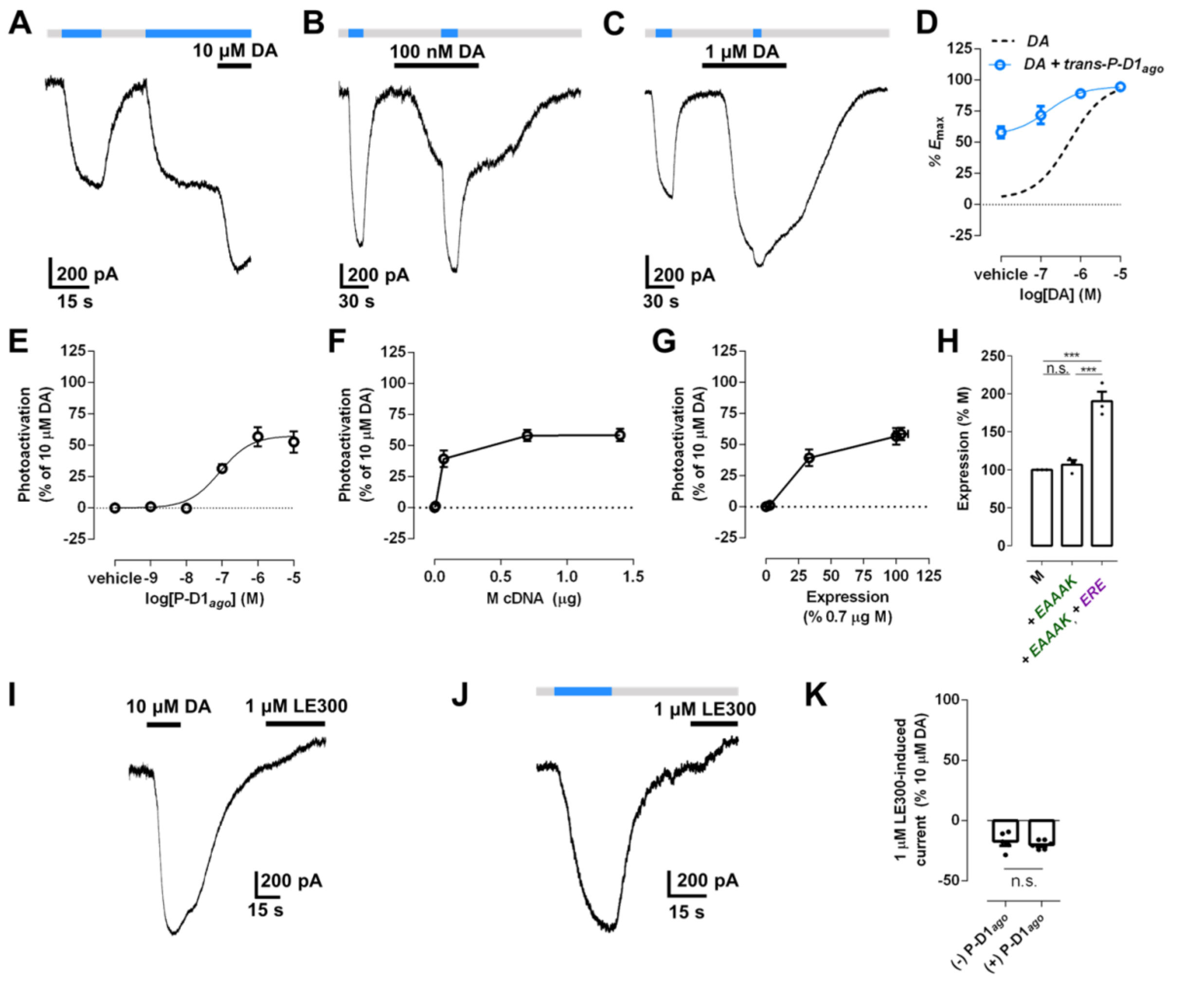

Supplementary Figure 10. Increasing the surface level of membrane-anchored P-D1$_{ago}$ enhances D1R photoactivation without increasing basal receptor activation. (A) P-D1$_{ago}$ tethered to M$_{EAAAK}$ only partially activates D1R under blue light (Fig. 2a,e), which could be because it is either a partial agonist or a full agonist that partially occupies the receptor. To test this, we evaluated photoactivation in the presence of DA. D1R can be further activated by a saturating concentration of DA (10 µM) when preactivated by P-D1$_{ago}$ tethered to M$_{EAAAK}$ according to the GIRK assay. Thus, unlike a partial agonist, P-D1$_{ago}$ tethered to M$_{EAAAK}$ did not diminish the ability of DA to fully activate D1R. **(B,C)** Consistent with the actions of a full agonist, P-D1$_{ago}$ tethered to M$_{EAAAK}$ further activated D1R in the presence of submaximal (100 nM, B) and near maximal (1 µM, C). **(D)** Summary of D1R photoactivation with P-D1$_{ago}$ tethered to M$_{EAAAK}$ in the presence of sub-saturating concentrations of DA. The dotted line represents the dose-response curve of activation of D1R by DA in the absence of P-D1$_{ago}$ tethered to M$_{EAAAK}$. n = 3 cells for 100 nM DA, n = 5 cells for 1 µM and 10 µM DA, and n = 10 cells for 10 nM DA. **(E)** The level of P-D1$_{ago}$ tethered

to M_{EAAAK} is not limited by submaximal SNAP-tag labeling with P-$D1_{ago}$. D1R photoactivation by P-$D1_{ago}$ tethered to M_{EAAAK} is shown as a function of the labeling concentration of P-$D1_{ago}$. M_{EAAAK} was labeled for 1 hr at 37 ºC with increasing concentrations of P-$D1_{ago}$. n = 3 cells for 1 nM, n = 4 cells for 10 nM and 100 nM, n = 5 cells for 10 µM, and n = 7 cells for 1 µM P-$D1_{ago}$. **(F)** The level of P-$D1_{ago}$ tethered to M_{EAAAK} is not limited by insufficient M_{EAAAK} cDNA. D1R photoactivation by P-$D1_{ago}$ tethered to M_{EAAAK} as a function of the amount of M_{EAAAK} DNA used to transfect HEK293T cells. M_{EAAAK} was labeled for 1 hr at 37 ºC with 1 µM P-$D1_{ago}$. n = 3-10 for each amount of DNA. For photoactivation, n = 3 cells for 0.07 µg, n = 4 cells for 0.007 µg and 1.4 µg, and n = 12 cells for 0.7 µg. **(G)** D1R photoactivation by P-$D1_{ago}$ tethered to M_{EAAAK} as a function of the surface levels M_{EAAAK}. Surface levels of M_{EAAAK} were quantified by labeling with BG-Alexa647 and measuring fluorescence using flow cytometry. n = 3 for 33% of 0.7 µg M expression, n = 4 cells for 3% and 104% of 0.7 µg M expression, and n = 8 for 100% of 0.7 µg M expression. **(H)** Surface levels of variants of M containing the lift peptide EAAAK and the endoplasmic reticulum export tag ERE. Surface levels were quantified by labeling M expressing cells with BG-Alexa647 and measuring fluorescence using flow cytometry. One-way ANOVA, F = 40.2, Tukey, p = 0.001. n = 3. **(I)** Representative trace of the effect of the D1R inverse agonist LE300 on basal inward-current of D1R-expressing HEK293T cells in the GIRK assay. **(J)** Representative trace of the effect of the D1R inverse agonist LE300 on basal inward-current of D1R and $M_{EAAAK:ERE}$ expressing HEK293T cells labeled with P-$D1_{ago}$ in the GIRK assay. **(K)** Summary of the effect of LE300 on basal inward current of D1R and $M_{EAAAK:ERE}$ expressing cells labeled or not labeled with P-$D1_{ago}$. Unpaired two-sided t-test, p = 0.46. n = 5 cells without P-$D1_{ago}$ and n = 6 cells with P-$D1_{ago}$. Error bars indicate S.E.M.

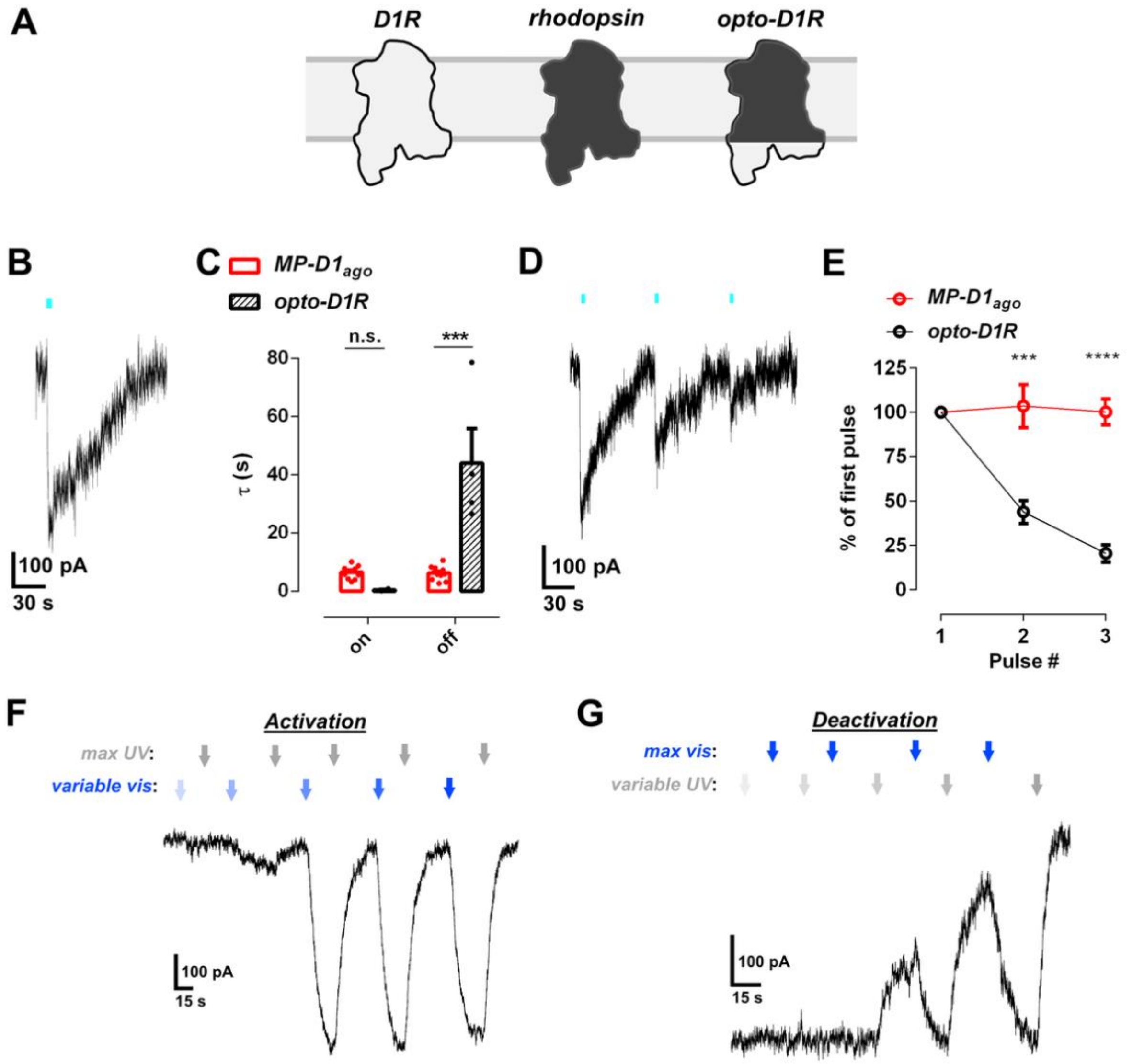

Supplementary Figure 11. Functional properties of MP-D1$_{ago}$ compared to opto-D1R. (A) Schematic representation of opto-D1R[62], a chimeric protein consisting of the transmembrane domain of rhodopsin in combination with the intracellular signaling components of D1R. When ectopically expressed in the target cell type, this engineered protein can be activated with light and engage D1R-mediated signaling with cell type and spatiotemporal specificity. **(B)** Representative trace of the activation of opto-D1R with 473 nm light in the GIRK assay. **(C)** D1R deactivates within seconds with MP-D1$_{ago}$, but opto-D1R takes greater than one minute to turn off completely. One-way ANOVA, $F = 25.4$, Tukey, ***$p < 0.001$. n = 11 cells for MP-D1$_{ago}$ and n = 4 for opto-D1R. **(D)** Representative trace of repeated cycles of activation of opto-D1R with 473 nm

light. **(E)** D1R photoactivation with MP-D1$_{ago}$ is repeatable with no loss in efficacy after multiple cycles. However, peak activation of opto-D1R drops off dramatically (~5-fold reduction after two cycles). Moreover, whereas MP-D1$_{ago}$ can sustain D1R in an active state following a brief exposure of light, opto-D1R requires continuous light exposure to remain active. One-way ANOVA, $F = 28.0$, Tukey, ***$p < 0.001$, ****$p < 0.0001$. $n = 3$ cells for MP-D1$_{ago}$ and $n = 8$ cells for opto-D1R. Representative traces of MP-D1$_{ago}$ photoactivation **(F)** and deactivation **(G)** with increasing light intensities. See Fig. 2h for summary. Arrows indicate a one second flash of light. Error bars indicate S.E.M.

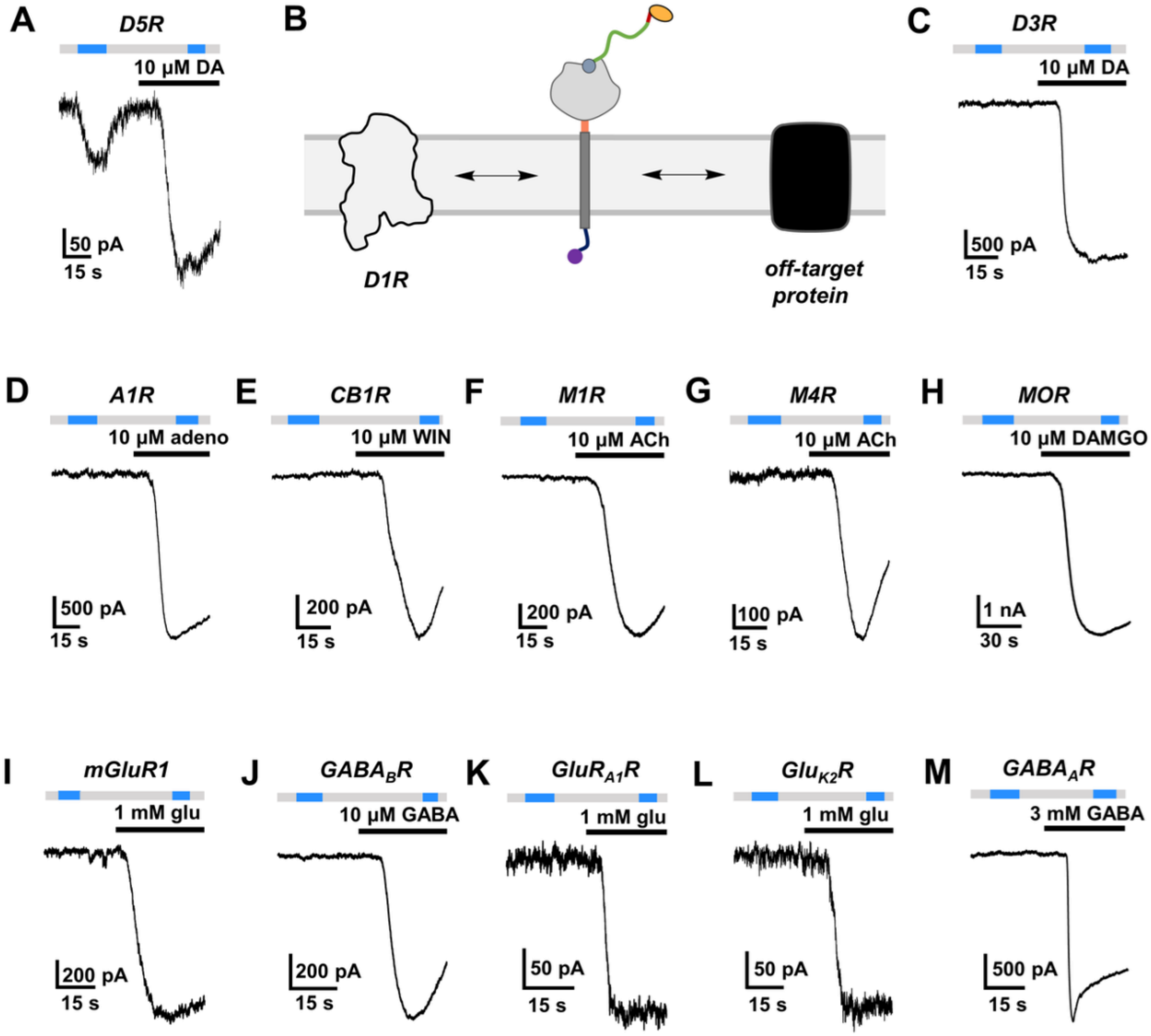

Supplementary Figure 12. MP-D1$_{ago}$ is a D1R/D5R selective-photoagonist. (A) MP-D1$_{ago}$ partially photoactivates D5R, the most closely related receptor to D1R (~80% homology within the transmembrane domain[63]). D5R was coexpressed with the chimeric G protein Gα_{is13}, allowing the receptor to couple to GIRK channels. **(B)** MPs like MP-D1$_{ago}$ are restricted to the surface of selected neurons, preventing them from binding the receptor of interest and off-target proteins in other neurons. However, MPs laterally diffuse in the plasma membrane and so can in principle interact with off-target proteins that are co-expressed in the same cell as the receptor of interest. Thus, an important part of MP selectivity depends on the inherent specificity of the photoswichable ligand. MP-D1$_{ago}$ has no effect on D3R **(C)**, a DAR that is coexpressed with D1R *in vivo*[64-66] but has low homology in the transmembrane domain (~39%[67]). MP-D1$_{ago}$ also has no effect on other

GPCRs that are expressed in D1R-expressing neurons *in vivo*, including adenosine receptor A1R[68] **(D)**, cannabinoid receptor CB1R[69] **(E)**, acetylcholine muscarinic receptors M1R[70] **(F)** and M4R[70] **(G)**, opioid receptor MOR[71] **(H)**, metabotropic glutamate receptor mGluR1[72] **(I)**, and GABA receptor $GABA_BR$[73] **(J)**. In all cases, GPCR-mediated GIRK channel activation was measured. D3R was coexpressed with $G\alpha_{oA}$, and M1R and mGluR1 were coexpressed with the chimeric G protein $G\alpha_{iq5}$, allowing the receptors to couple to GIRK channels in HEK293T cells. MP-$D1_{ago}$ also has no effect on ionotropic receptors that are widely expressed in the brain: the AMPA receptor $GluR_{A1}R$ **(K)**, the kainite receptor $Glu_{K2}R$ **(L)**, and the GABA receptor $GABA_AR_{\alpha1\beta2\gamma2}$ **(M)**. To prevent the desensitization, a non-desensitizing mutant of $GluR_{A1}R$ (L497Y) was used and $Glu_{K2}R$ was measured in the presence of 0.3 mg/mL concanavalin A. Abbreviations: dopamine = DA, adenosine = adeno, WIN = WIN55,212-2, acetylcholine = ACh, glu = glutamate.

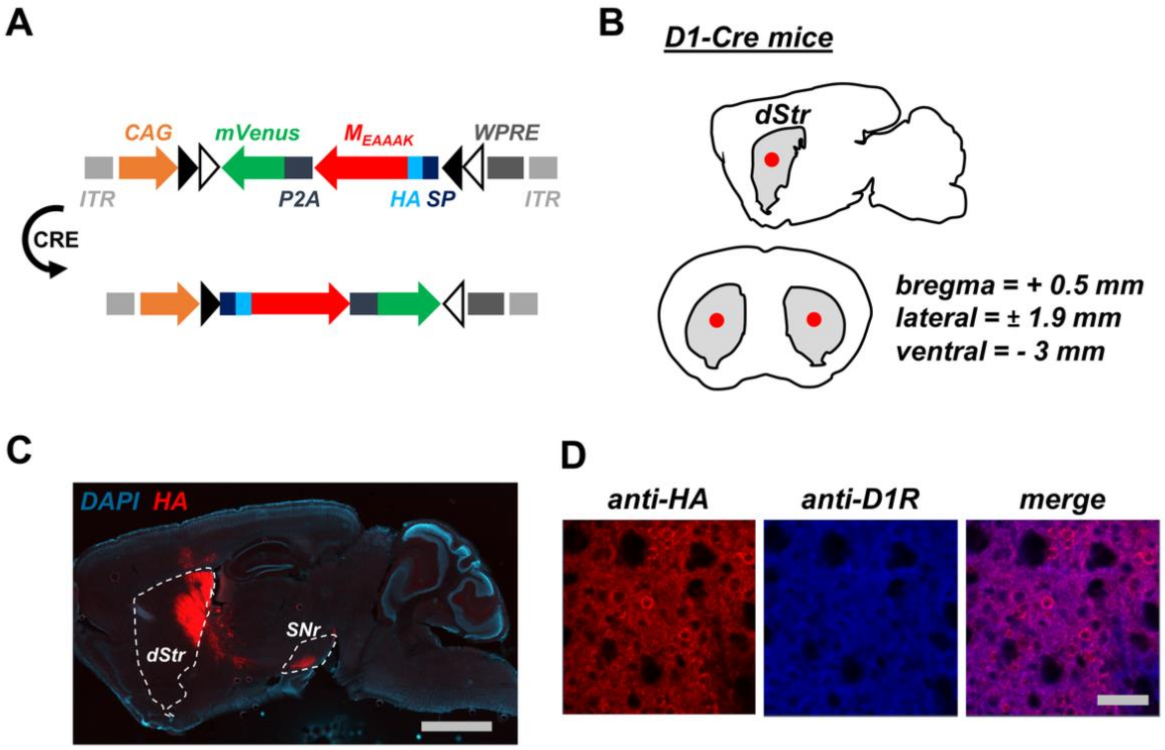

Supplementary Figure 13. Expression of the membrane anchor component of MP-D1$_{ago}$, M$_{EAAAK:ERE}$. **(A)** Schematic representation of AAV5-CAG-DIO-SP-HA-M$_{EAAAK:ERE}$-P2A-mVenus. The expression cassette is oriented in the forward direction in the presence of Cre-recombinase, which recognizes flox sites (black and white arrows). Abbreviations: ITR = inverted terminal repeat, HA = hemagglutinin tag, SP = hemagglutinin signal peptide, WPRE = woodchuck hepatitis virus posttranscriptional regulatory element. **(B)** The AAV was injected into the dorsal striatum (dStr) of D1-Cre mice (sagittal on top and coronal on bottom). **(C)** Sagittal slices were stained with an anti-HA antibody. M$_{EAAAK:ERE}$ expressed in direct pathway medium spiny neurons (dMSNs) of the dStr, which project to the substantia nigra reticulata (SNr). Grey bar = 2 mm. Blue = DAPI staining, red = HA-tag (M$_{EAAAK:ERE}$) staining. Representative of brains from n = 3 mice. **(D)** Coronal slices were stained with anti-D1R and anti-HA antibodies, which indicated that M$_{EAAAK:ERE}$ colocalizes with D1R in dMSNs. Grey bar = 100 µm. Red = HA-tag (M$_{EAAAK:ERE}$) staining, blue = D1R staining. Representative of brains from n = 3 mice.

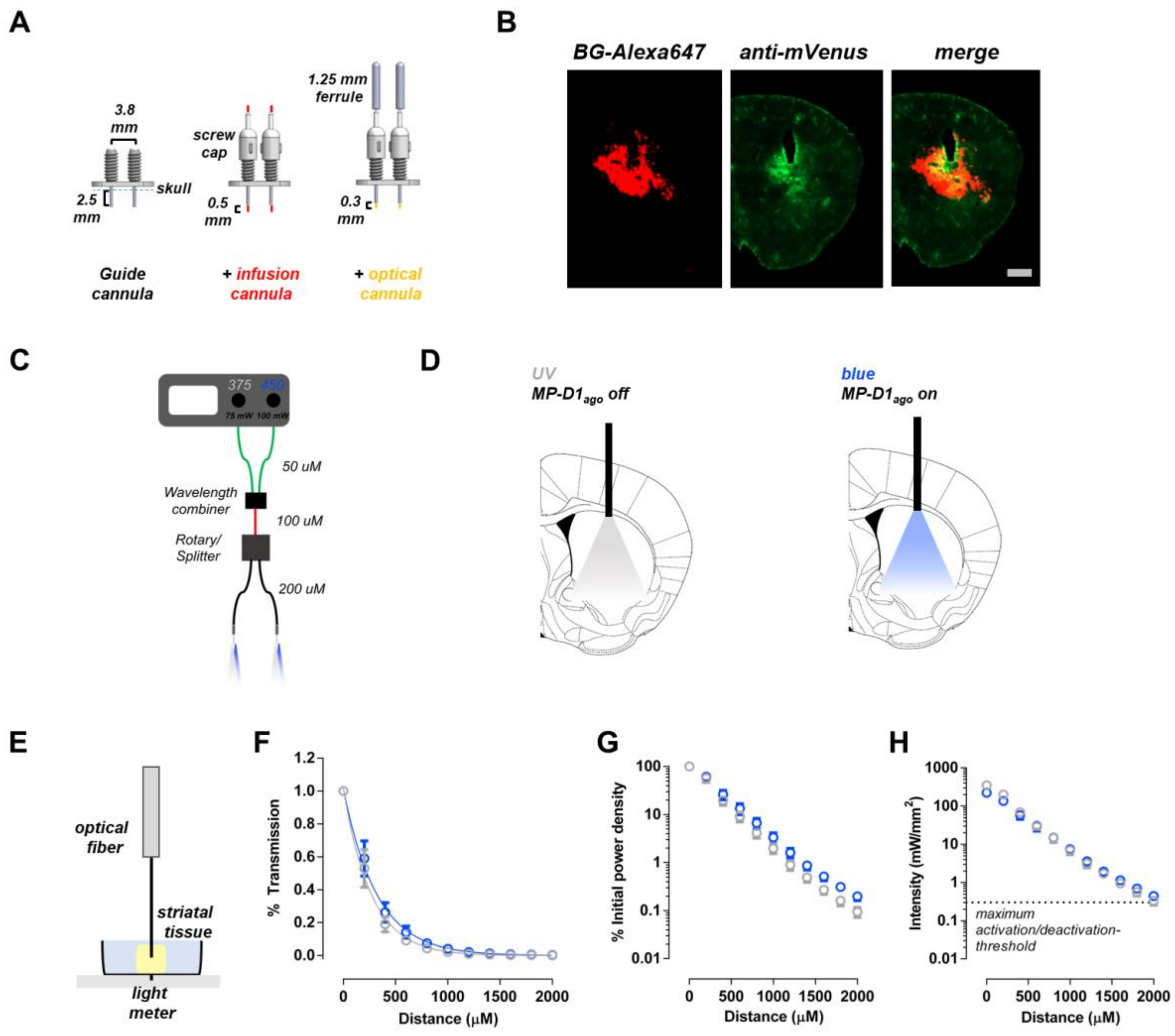

Supplementary Figure 14. Design and optimization of a bilateral infusion and dual color light delivery system for mouse brain. (A) A custom bilateral head implant that targets the dorsal striatum with interchangeable infusion and optical cannulas. The infusion cannula reaches the same depth as the AAV injection site (3 mm below the skull) and the optical cannula is placed above it (2.8 mm below the skull) to maximize the illumination of the AAV expression area (400 nL of AAV per hemisphere). **(B)** The fluorescent dye BG-Alexa647 (1 µL at 100 µM) was infused into the dStr of mice expressing the M and mVenus in dMSNs. Sections were taken three hours after infusion to allow for dye conjugation to the SNAP-tag in the M. There was robust overlap between the dye and mVenus (71 ± 5%; n = 3 hemispheres). Staining was not observed in the absence of the M (*data not shown*). Grey bar = 1 mm. **(C)** Schematic of a laser system (Doric) that delivers two wavelengths of light from two fibers. The light from a UV laser (375 nm) and a blue laser (450 nm) are combined with a wavelength combiner, and then divided by a wavelength

splitter into two fibers, one for each side of the brain. The splitter rotates to prevent twining of the fibers, which are attached to the head of a mouse via the custom head implant described above. **(D)** MP-D1$_{ago}$ requires two wavelengths of light: UV light to turn it off (left) and blue light to turn it on (right). If these wavelengths are not calibrated, D1R will be heterogeneously activated and deactivated with MP-D1$_{ago}$ across the brain. The efficiency of light propagation through brain tissue depends on wavelength[74] and brain area[75]. **(E)** To measure the propagation of UV light and blue light through the striatum, an optical fiber was placed above and progressively lowered downwards through freshly excised striatal tissue. A light meter with a pinhole the size of the end of the optical fiber (200 µM) was placed below the tissue to measure the efficiency of transmission **(F)** and the percent of the initial power density at the tip of the fiber **(G)**, similar to previous work[76]. As expected, the longer blue wavelength of light propagated through brain tissue more efficiently than the shorter UV wavelength[74]. n = 6 hemispheres per condition. Error bars indicate S.E.M. **(H)** The light output of each wavelength at the fiber tip was adjusted (375 nm laser = 9.3 mW; 450 nm laser = 6 mW) to achieve similar intensities across striatal tissue. This analysis indicates that D1R can be maximally activated or deactivated with MP-D1$_{ago}$ across ~2 mm of striatal tissue. Because only a brief flash of light is required to persistently turn on or off the receptor, heating effects associated with typical opsins are less likely[77]. n = 6 hemispheres per condition. Error bars indicate S.E.M.

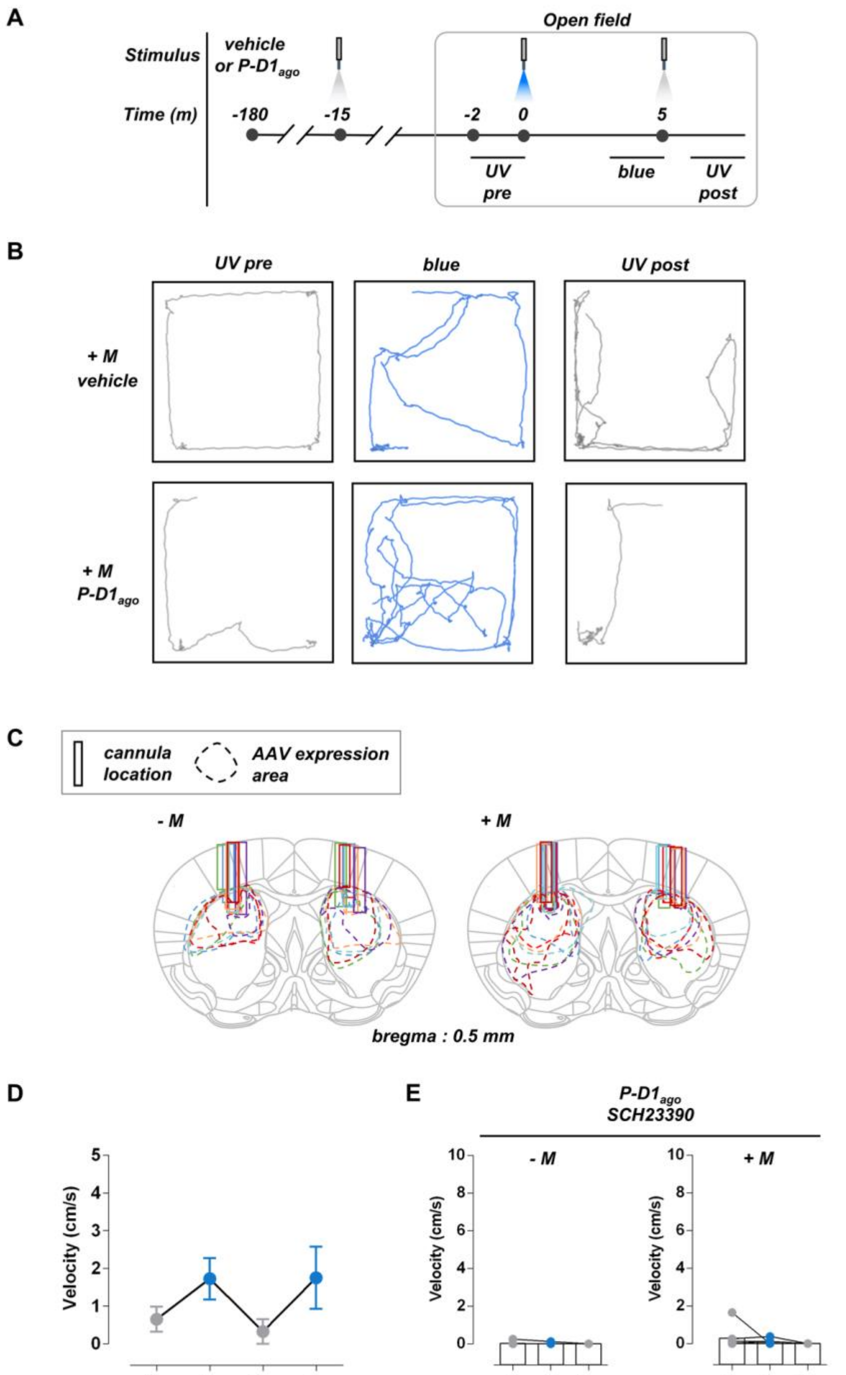
A
Open field
Stimulus
vehicle or P-D1ago
Time (m)
-180
-15
-2
0
5
UV pre
blue
UV post
B
UV pre
blue
UV post
+ M vehicle
+ M P-D1ago
C
cannula location
AAV expression area
- M
+ M
bregma : 0.5 mm
D
Velocity (cm/s)
UV
blue
UV
blue
E
P-D1ago SCH23390
- M
+ M
Velocity (cm/s)
UV (pre)
blue
UV (post)

Supplementary Figure 15. Extended data for the motor effect of MP-D1$_{ago}$-induced activation of dStr-dMSN D1Rs. (A) D1-Cre mice expressing mVenus (-*M*) or M$_{EAAAK:ERE}$ and mVenus (+*M*) in dStr-dMSNs received bilateral dorsal striatum (dStr)-infusions (1 µL) of either vehicle (1% DMSO) or the inactive *cis* configuration of P-D1$_{ago}$ in 1% DMSO (*cis;* 100 µM) three hours prior to being placed in an open field. Because P-D1$_{ago}$ slowly relaxes from the inactive *cis* to the active *trans* configuration over time, mice were exposed to UV light (5 s) approximately 15 minutes before the open field test to ensure that the P-D1$_{ago}$ was in the off-state. MP-D1$_{ago}$ was switched to the on-state with blue light (1 s) at zero minutes and switched back to the off-state with UV light (1 s) at five minutes. For the statistical analysis, the locomotion speed of each mouse was averaged over the following two-minute periods: (i) just before exposure to blue light (*UV pre*), (ii) three minutes after exposure to blue light (*blue*), (iii) and one minute after exposure to the second flash of UV light (*UV post*). **(B)** Representative locomotor tracks of +*M* mice treated with vehicle or P-D1$_{ago}$ during the *UV pre*, *blue*, and *UV post* periods. Vehicle and P-D1$_{ago}$ tracks are shown for a single mouse. **(C)** Summary of the AAV expression profile and cannula locations in -*M* or +*M* mice. Each color is associated with a single mouse within a group. **(D)** The motor effect of MP-D1$_{ago}$ in dStr-dMSNs is repeatable. n = 4 mice. **(E)** Summary of the average speed of MP-D1$_{ago}$ mice treated with SCH23390 (0.5 mg/kg) during the *UV pre*, *blue*, and *UV post* periods. SCH23390 was administered 30 minutes before MP-D1$_{ago}$ was switched to the on-state with blue light. RM one-way ANOVA, F-values from left to right: 1.0, 1.2, Bonferroni. There was no significant difference between any of the conditions. n = 7 mice for each condition. Error bars indicate S.E.M.

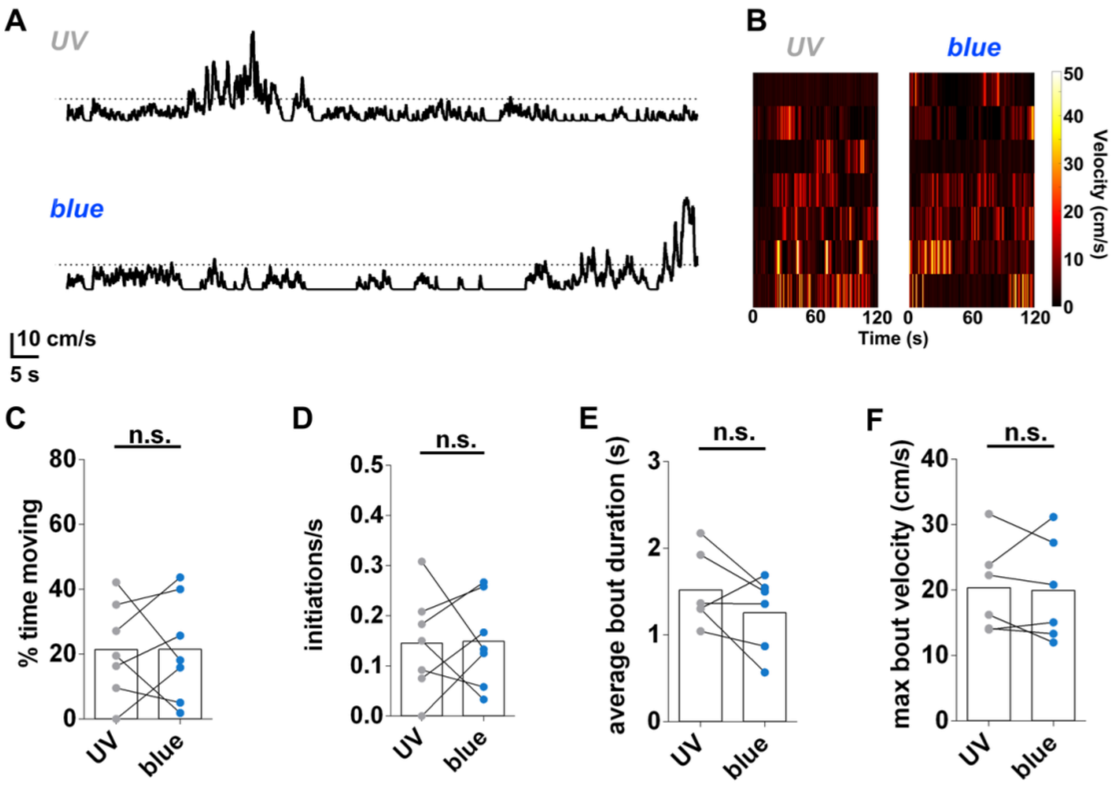

Supplementary Figure 16. M expression in dStr-dMSNs has no effect on movement in the absence of P-D1$_{ago}$. (A) Example movement dynamics of 1% DMSO (no P-D1$_{ago}$) treated M-expressing mouse under illumination with UV (*top panel*) and blue light (*bottom panel*), as in the two-minute *UV pre* and *blue* periods shown for P-D1$_{ago}$ in Supplementary Fig. 15a. The dotted lines represent the velocity threshold used to identify movement bouts. **(B)** Movement dynamics of vehicle alone (no P-D1$_{ago}$) M-expressing mice under UV light and blue light, in absence of P-D1$_{ago}$ (n = 7). **(C-F)** Average percent of time in motion (C)**,** average initiations per second (D), average movement bout duration (E)**,** and average maximum movement bout velocity (F). Paired two-tailed t-test, p-values from left to right: 0.99, 0.94, 0.18, 0.55. n from left to right: 7, 7, 6, 6 mice. In (E) and (F) mice were excluded if there were no movement bouts under UV light.

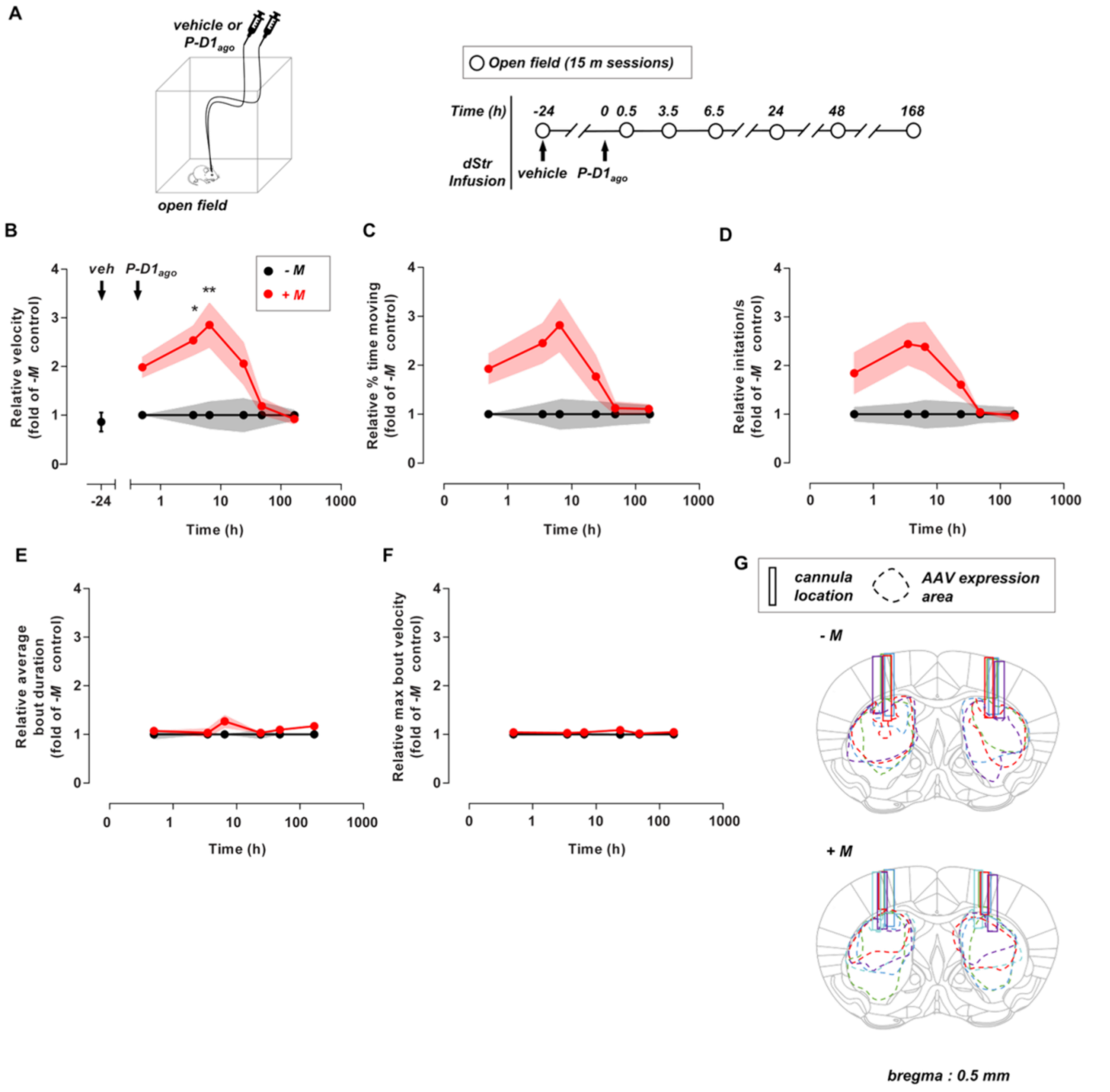

Supplementary Figure 17. MP-D1$_{ago}$ can be used to chronically activate dStr-dMSN D1Rs. (A) D1-Cre mice expressing mVenus (-*M*) or M$_{EAAAK:ERE}$ and mVenus (+*M*) in dStr-dMSNs received bilateral dStr-infusions of vehicle (1% DMSO; 1 µL) and were placed in an open field for 15 minutes. The following day, they received bilateral dStr-infusions of the active form of P-D1$_{ago}$ (*trans;* 100 µM; 1 µL) and were placed in the open field 0.5, 3.5, 6.5, 24, 48, and 168 hours later for 15 minutes each time. Shown is the time course in response to vehicle or P-D1$_{ago}$ of **(B)** locomotion **(C)** average percent of the time in motion**, (D)** average initiations per second, **(E)** average movement bout duration, and the **(F)** the average maximum movement bout velocity. Datasets were normalized to the mean of each parameter for the -*M* group at each time point. For panel B, One-way ANOVA, F = 6.3, Bonferroni, *p < 0.05, **p < 0.01. n = 4 mice in the -*M* group

and n = 5 mice in the +*M* group for all time points except 6.5 hours, where n = 4. Error bands indicate S.E.M. **(G)** Summary of the AAV expression profile and cannula locations in -*M* or +*M* mice. Each color is associated with a single mouse within a group.

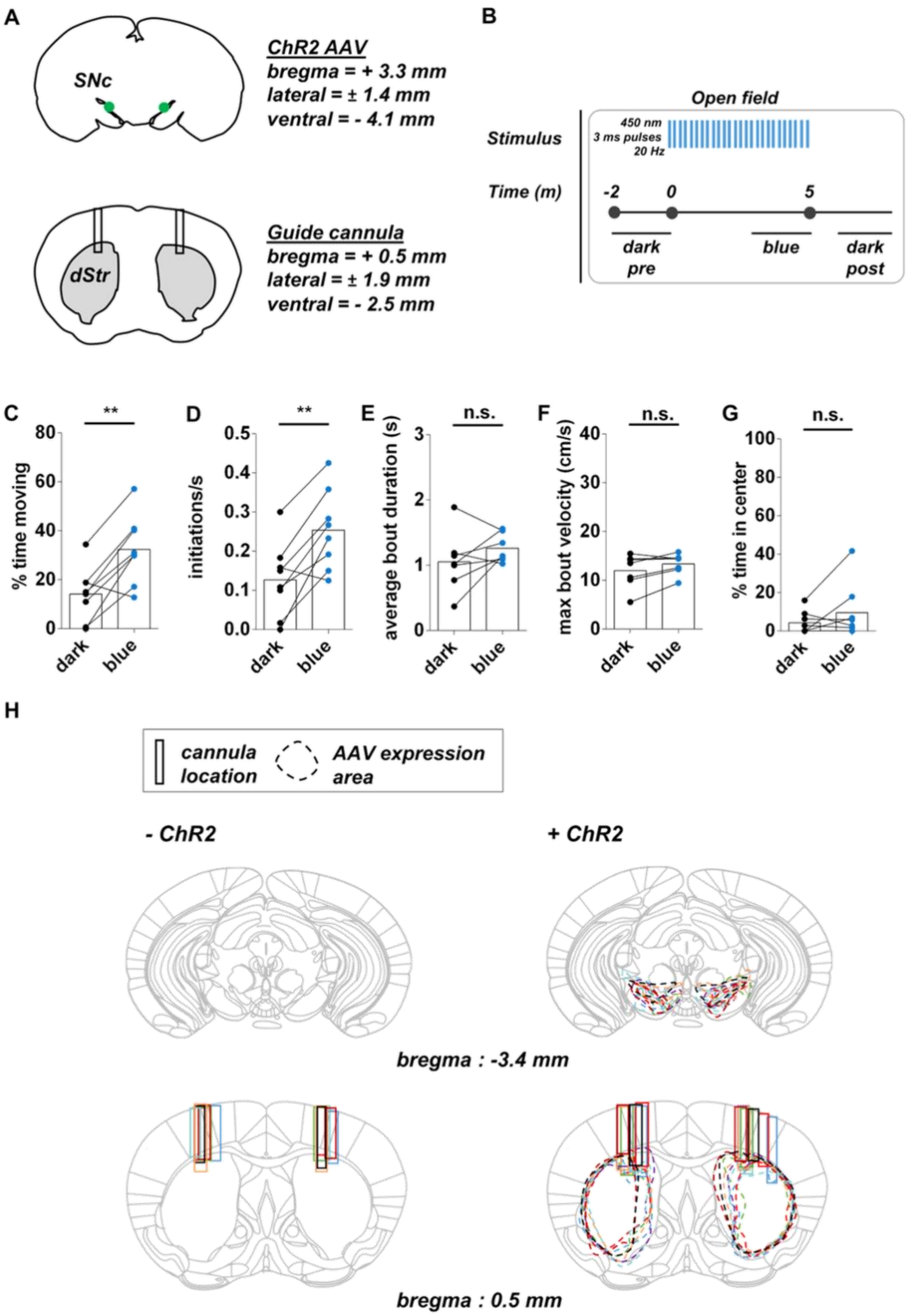
A
SNc
ChR2 AAV
bregma = + 3.3 mm
lateral = ± 1.4 mm
ventral = - 4.1 mm
dStr
Guide cannula
bregma = + 0.5 mm
lateral = ± 1.9 mm
ventral = - 2.5 mm
B
Open field
Stimulus
450 nm
3 ms pulses
20 Hz
Time (m)
-2
0
5
dark pre
blue
dark post
C
% time moving
**
dark
blue
D
initiations/s
**
E
average bout duration (s)
n.s.
F
max bout velocity (cm/s)
n.s.
G
% time in center
n.s.
H
cannula location
AAV expression area
- ChR2
+ ChR2
bregma : -3.4 mm
bregma : 0.5 mm

Supplementary Figure 18. Extended data for the motor effect of optogenetic activation of SNc terminals in the dStr with ChR2. (A) Coronal sections indicating the ChR2 AAV injection site in the SNc (green circle) and the guide cannula implant site in the dStr (black rectangle). Control mice were implanted with a guide cannula but did not receive an AAV injection. **(B)** Shown is a schematic representation of the behavioral paradigm used to test movement in the ChR2 mice. The mice were placed in an open field and stimulated for five minutes with blue light (450 nm, ~7 mW at fiber tip, 3 ms pulses, 20 Hz). For the statistical analyses below, the speed of each mouse was averaged over the following two-minute periods: (i) just before exposure to blue light (*dark pre*), (ii) three minutes after exposure to blue light (*blue*), (iii) and one minute after exposure to blue light (*dark post*). Shown for ChR2 mice during the *dark pre* and *blue* periods is **(C)** average percent of the time in motion**, (D)** average initiations per second, **(E)** average movement bout duration**, (F)** the average maximum movement bout velocity, and **(G)** the percentage of the time that the mice were in the center of the open field. Paired two-tailed t-test, p-values from left to right: **p = 0.002, **p = 0.003, 0.216, 0.071, 0.211. n from left to right: 8, 8, 7, 7, 8 mice. For Supplementary Fig. 18e and f, mice were excluded if there were no movement bouts in the dark. **(H)** Summary of the AAV expression profile and/or cannula locations of mice with and without ChR2. Each color is associated with a single mouse within a group.

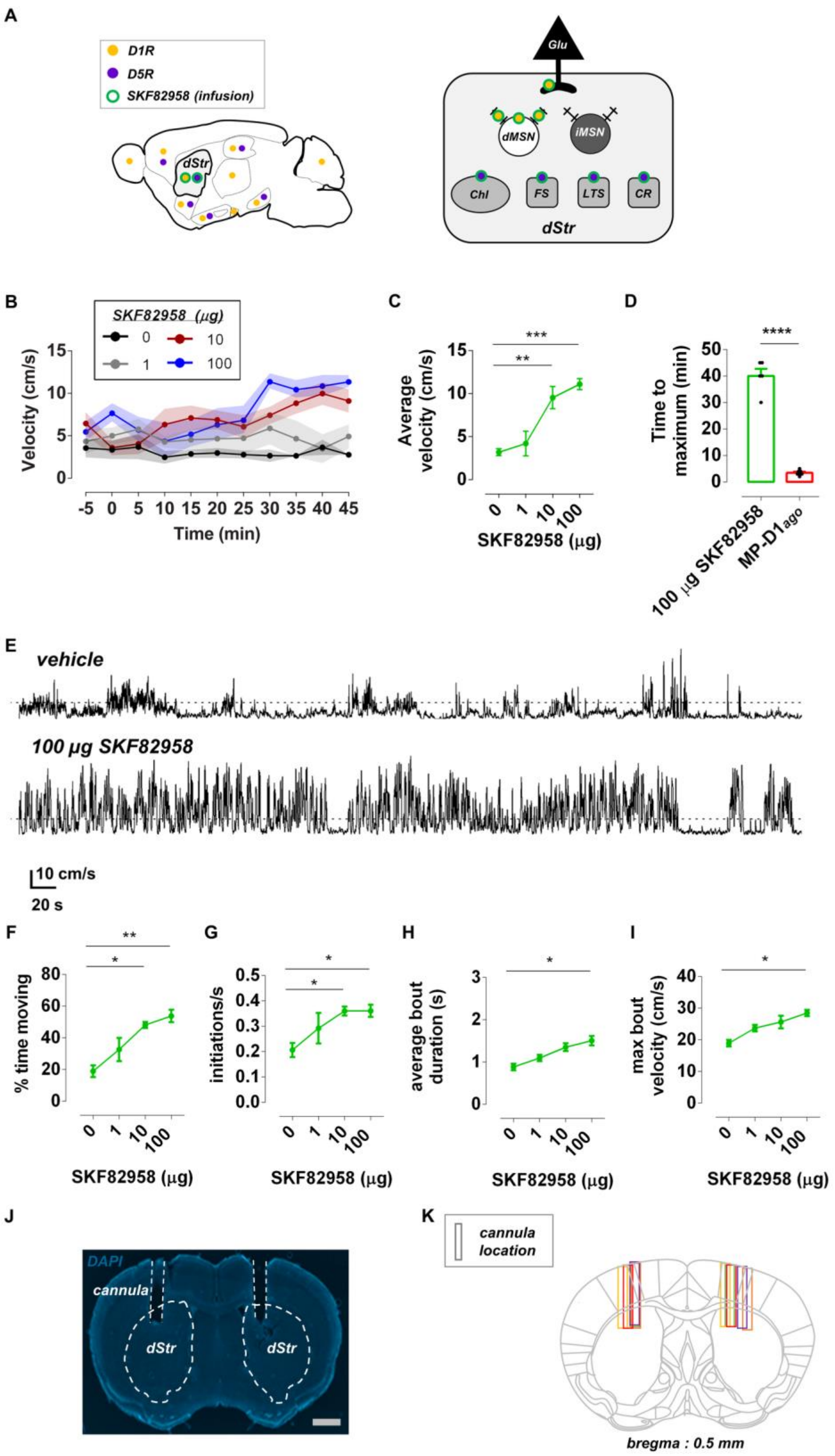

A
D1R
D5R
SKF82958 (infusion)
dStr
Glu
dMSN
iMSN
ChI
FS
LTS
CR
dStr
B
SKF82958 (μg)
0
1
10
100
Velocity (cm/s)
Time (min)
C
Average velocity (cm/s)
SKF82958 (μg)
D
Time to maximum (min)
100 μg SKF82958
MP-D1ago
E
vehicle
100 μg SKF82958
10 cm/s
20 s
F
% time moving
SKF82958 (μg)
G
initiations/s
SKF82958 (μg)
H
average bout duration (s)
SKF82958 (μg)
I
max bout velocity (cm/s)
SKF82958 (μg)
J
DAPI
cannula
dStr
dStr
K
cannula location
bregma : 0.5 mm

Supplementary Figure 19. Comparison of the motor effect of a dStr-infusion of the full D1R agonist SKF82958 and dStr-dMSN D1R activation with MP-D1$_{ago}$. (A) An infusion of SKF82958 directly to the dorsal striatum (dStr) activates D1R and D5R in various cell types of this brain area and could diffuse into other brain areas. **(B)** Time course of the effect of a bilateral dStr-infusion of SKF82958 (400 nL per hemisphere) on the locomotion of D1-Cre mice in an open field (vehicle of 100% DMSO). n = 5 mice per condition. Error bands indicate S.E.M. **(C)** Average velocity in the last 10 minutes of the open field test. One-way ANOVA, F = 14.3, Bonferroni, **p < 0.01, ***p < 0.001. n = 5 mice per condition. **(D)** Time to maximum locomotion with SKF82958 or MP-D1$_{ago}$. Unpaired two-sided t-test, ****p < 0.0001. n = 5 for SKF82958, n = 7 mice for MP-D1$_{ago}$. **(E)** Example movement dynamics of a mouse treated with vehicle or 100 µg SKF82958. Shown for SKF82958-treated mice is the **(F)** average percent of the time in motion**, (G)** average initiations per second, **(H)** average movement bout duration**,** and **(I)** the average maximum movement bout velocity. RM one-way ANOVA, F-values from left to right: 17.67, 8.61, 6.97, 8.88. Bonferroni, *p < 0.05, **p < 0.01. n = 5 mice. **(J)** Representative coronal brain slice from a mouse infused with SKF82958. grey bar = 1 mm. blue = DAPI staining. Representative of brains from n = 5 mice. **(K)** Summary of cannula locations for individual mice. Each color is associated with a single mouse. Error bars indicate S.E.M.

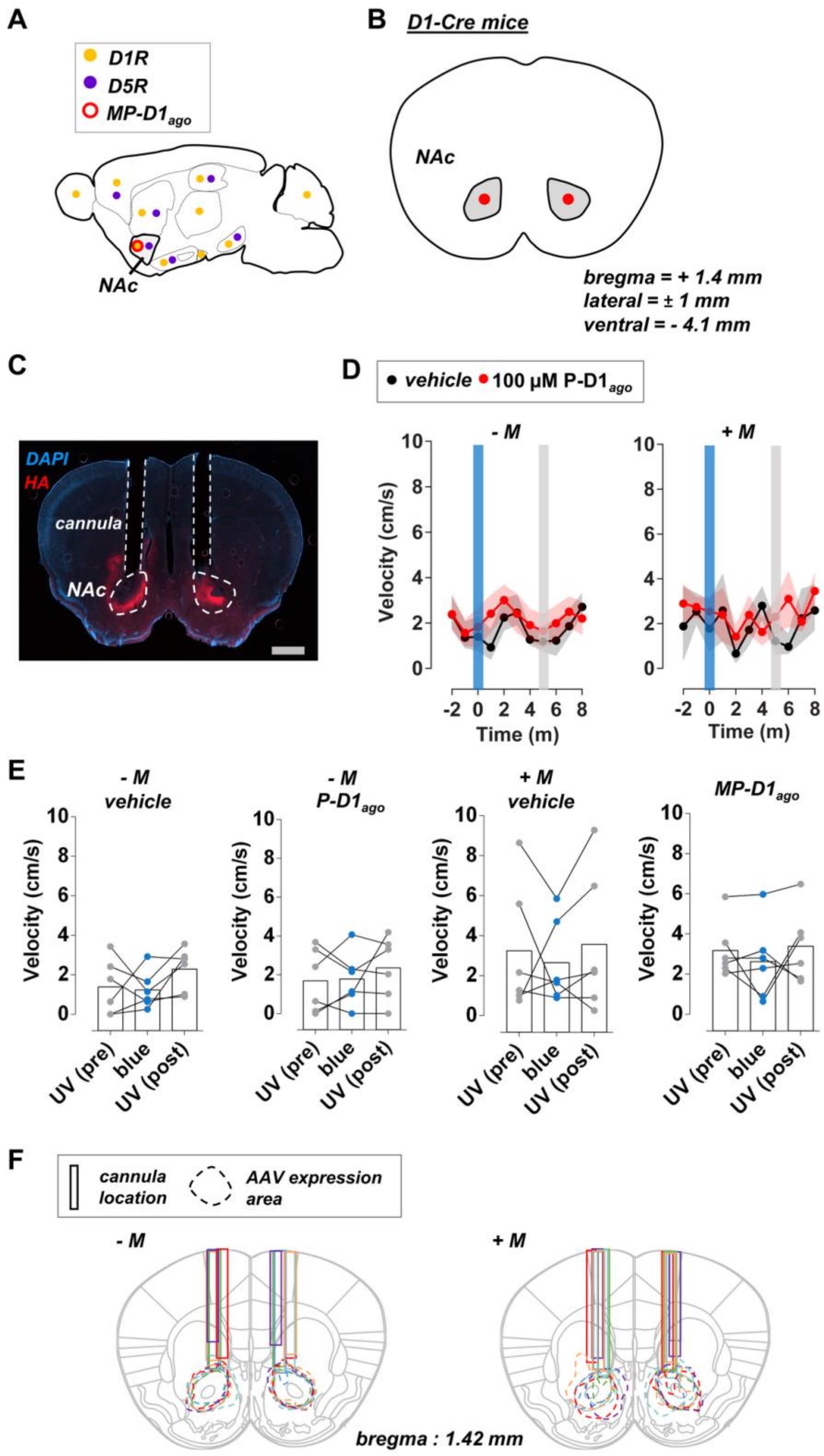

Supplementary Figure 20. Activation of D1Rs in MSNs of the vStr with MP-D1$_{ago}$ has no effect on movement. (A) D1Rs in NAc-dMSNs were selectively targeted with MP-D1$_{ago}$. **(B)**

Schematic of mouse brain (coronal section) highlighting the location of the nucleus accumbens (NAc). An AAV encoding mVenus or the membrane-anchor $M_{EAAAK:ERE}$ and mVenus was injected into the NAc of D1-Cre mice (red dot). **(C)** Expression of $M_{EAAAK:ERE}$ in the NAc. grey bar = 1 mm. blue = DAPI staining, red = HA-tag ($M_{EAAAK:ERE}$) staining. Representative of brains from n = 6 mice. **(D)** The speed of mice with MP-$D1_{ago}$ in NAc-dMSNs increased in response to a brief flash of blue light (450 nm, ~6 mW, 1 s) and returned to baseline after a brief flash of UV light (375 nm, ~9 mW, 1 s). There was no effect under any condition. **(E)** The speed of each mouse was averaged over the following two-minute periods: (i) just before exposure to blue light (*UV pre*), (ii) three minutes after exposure to blue light (*blue*), (iii) and one minute after exposure to the second flash of UV light (*UV post*). There was no significant difference between any condition. RM one-way ANOVA, F-values from left to right: 1.9, 0.6, 0.4, 0.8, Bonferroni. n = 6 mice for each condition. **(F)** Summary of the AAV expression profile and cannula locations in -*M* or +*M* mice. Each color is associated with a single mouse within a group. Error bars indicate S.E.M.

References

1. Zheng, J. et al. A generic magnetic microsphere platform with "clickable" ligands for purification and immobilization of targeted proteins. *ACS applied materials & interfaces* **7**, 7241-7250 (2015).
2. Fenno, L., Yizhar, O. & Deisseroth, K. The Development and Application of Optogenetics. *Annual Review of Neuroscience, Vol 34* **34**, 389-412 (2011).
3. Rossi, M.A., Sukharnikova, T., Hayrapetyan, V.Y., Yang, L.C. & Yin, H.H. Operant Self-Stimulation of Dopamine Neurons in the Substantia Nigra. *PloS one* **8** (2013).
4. Tsai, H.C. et al. Phasic Firing in Dopaminergic Neurons Is Sufficient for Behavioral Conditioning. *Science* **324**, 1080-1084 (2009).
5. Beier, K.T. et al. Circuit Architecture of VTA Dopamine Neurons Revealed by Systematic Input-Output Mapping. *Cell* **162**, 622-634 (2015).
6. Lerner, T.N. et al. Intact-Brain Analyses Reveal Distinct Information Carried by SNc Dopamine Subcircuits. *Cell* **162**, 635-647 (2015).
7. Stuber, G.D., Hnasko, T.S., Britt, J.P., Edwards, R.H. & Bonci, A. Dopaminergic Terminals in the Nucleus Accumbens But Not the Dorsal Striatum Corelease Glutamate. *Journal of Neuroscience* **30**, 8229-8233 (2010).
8. Lammel, S. et al. Input-specific control of reward and aversion in the ventral tegmental area. *Nature* **491**, 212-217 (2012).
9. Threlfell, S. et al. Striatal Dopamine Release Is Triggered by Synchronized Activity in Cholinergic Interneurons. *Neuron* **75**, 58-64 (2012).
10. Zhang, H. & Sulzer, D. Glutamate spillover in the striatum depresses dopaminergic transmission by activating group I metabotropic glutamate receptors. *The Journal of neuroscience : the official journal of the Society for Neuroscience* **23**, 10585-10592 (2003).
11. Matsuda, W. et al. Single Nigrostriatal Dopaminergic Neurons Form Widely Spread and Highly Dense Axonal Arborizations in the Neostriatum. *Journal of Neuroscience* **29**, 444-453 (2009).
12. Garris, P.A., Ciolkowski, E.L., Pastore, P. & Wightman, R.M. Efflux of Dopamine from the Synaptic Cleft in the Nucleus-Accumbens of the Rat-Brain. *Journal of Neuroscience* **14**, 6084-6093 (1994).
13. Ford, C.P. The Role of D2-Autoreceptors in Regulating Dopamine Neuron Activity and Transmission. *Neuroscience* **282**, 13-22 (2014).
14. Dumartin, B., Doudnikoff, E., Gonon, F. & Bloch, B. Differences in ultrastructural localization of dopaminergic D1 receptors between dorsal striatum and nucleus accumbens in the rat. *Neuroscience letters* **419**, 273-277 (2007).
15. Wang, H. & Pickel, V.M. Dopamine D2 receptors are present in prefrontal cortical afferents and their targets in patches of the rat caudate-putamen nucleus. *The Journal of comparative neurology* **442**, 392-404 (2002).
16. Tritsch, N.X., Ding, J.B. & Sabatini, B.L. Dopaminergic neurons inhibit striatal output through non-canonical release of GABA. *Nature* **490**, 262-+ (2012).
17. Joyce, J.N. & Millan, M.J. Dopamine D3 receptor antagonists as therapeutic agents. *Drug discovery today* **10**, 917-925 (2005).
18. Sun, F. et al. A Genetically Encoded Fluorescent Sensor Enables Rapid and Specific Detection of Dopamine in Flies, Fish, and Mice. *Cell* **174**, 481-496 e419 (2018).
19. Patriarchi, T. et al. Ultrafast neuronal imaging of dopamine dynamics with designed genetically encoded sensors. *Science* **360** (2018).
20. Traynelis, S.F. et al. Glutamate receptor ion channels: structure, regulation, and function. *Pharmacological reviews* **62**, 405-496 (2010).

21. Hull, K., Morstein, J. & Trauner, D. In Vivo Photopharmacology. *Chem Rev* **118**, 10710-10747 (2018).
22. Atasoy, D. & Sternson, S.M. Chemogenetic Tools for Causal Cellular and Neuronal Biology. *Physiological reviews* **98**, 391-418 (2018).
23. Urban, D.J. & Roth, B.L. DREADDs (Designer Receptors Exclusively Activated by Designer Drugs): Chemogenetic Tools with Therapeutic Utility. *Annual review of pharmacology and toxicology* **55**, 399-417 (2015).
24. Airan, R.D., Thompson, K.R., Fenno, L.E., Bernstein, H. & Deisseroth, K. Temporally precise in vivo control of intracellular signalling. *Nature* **458**, 1025-1029 (2009).
25. Broichhagen, J.D., A;, Levitz, J.; Sokol K.; Leippe, P.; Konrad, D.; Isacoff, EY; Trauner, D. Orthogonal Optical Control of a G Protein-Coupled Receptor with a SNAP-Tethered Photochromic Ligand. *ACS Central Science* **1**, 383-393 (2015).
26. Levitz, J. et al. Dual optical control and mechanistic insights into photoswitchable group II and III metabotropic glutamate receptors. *Proceedings of the National Academy of Sciences of the United States of America* **114**, E3546-E3554 (2017).
27. Acosta-Ruiz, A. et al. Branched Photoswitchable Tethered Ligands Enable Ultra-efficient Optical Control and Detection of G Protein-Coupled Receptors In Vivo. *Neuron* **105**, 446-463 e413 (2020).
28. Ibanez-Tallon, I. et al. Tethering naturally occurring peptide toxins for cell-autonomous modulation of ion channels and receptors in vivo. *Neuron* **43**, 305-311 (2004).
29. Shields, B.C. et al. Deconstructing behavioral neuropharmacology with cellular specificity. *Science* **356** (2017).
30. Schmidt, D., Tillberg, P.W., Chen, F. & Boyden, E.S. A fully genetically encoded protein architecture for optical control of peptide ligand concentration. *Nature communications* **5**, 3019 (2014).
31. Nakamura, T. et al. Distinct motor impairments of dopamine D1 and D2 receptor knockout mice revealed by three types of motor behavior. *Front Integr Neurosci* **8**, 56 (2014).
32. Karlsson, R.M., Hefner, K.R., Sibley, D.R. & Holmes, A. Comparison of dopamine D1 and D5 receptor knockout mice for cocaine locomotor sensitization. *Psychopharmacology* **200**, 117-127 (2008).
33. Gallardo, C.M. et al. Dopamine receptor 1 neurons in the dorsal striatum regulate food anticipatory circadian activity rhythms in mice. *Elife* **3**, e03781 (2014).
34. Kobayashi, M. et al. Simultaneous absence of dopamine D1 and D2 receptor-mediated signaling is lethal in mice. *Proceedings of the National Academy of Sciences of the United States of America* **101**, 11465-11470 (2004).
35. McDougall, S.A. et al. Importance of D(1) receptors for associative components of amphetamine-induced behavioral sensitization and conditioned activity: a study using D(1) receptor knockout mice. *Psychopharmacology* **183**, 20-30 (2005).
36. Abraham, A.D., Neve, K.A. & Lattal, K.M. Effects of D1 receptor knockout on fear and reward learning. *Neurobiol Learn Mem* **133**, 265-273 (2016).
37. Tran, A.H. et al. Dopamine D1 receptors involved in locomotor activity and accumbens neural responses to prediction of reward associated with place. *Proceedings of the National Academy of Sciences of the United States of America* **102**, 2117-2122 (2005).
38. McNamara, F.N. et al. Congenic D1A dopamine receptor mutants: ethologically based resolution of behavioural topography indicates genetic background as a determinant of knockout phenotype. *Neuropsychopharmacology : official publication of the American College of Neuropsychopharmacology* **28**, 86-99 (2003).
39. Drago, J. et al. Altered striatal function in a mutant mouse lacking D1A dopamine receptors. *Proceedings of the National Academy of Sciences of the United States of America* **91**, 12564-12568 (1994).

40. Urs, N.M., Daigle, T.L. & Caron, M.G. A dopamine D1 receptor-dependent beta-arrestin signaling complex potentially regulates morphine-induced psychomotor activation but not reward in mice. *Neuropsychopharmacology : official publication of the American College of Neuropsychopharmacology* **36**, 551-558 (2011).
41. El-Ghundi, M. et al. Spatial learning deficit in dopamine D(1) receptor knockout mice. *European journal of pharmacology* **383**, 95-106 (1999).
42. Karasinska, J.M., George, S.R., El-Ghundi, M., Fletcher, P.J. & O'Dowd, B.F. Modification of dopamine D(1) receptor knockout phenotype in mice lacking both dopamine D(1) and D(3) receptors. *European journal of pharmacology* **399**, 171-181 (2000).
43. Horn, A.S., Tepper, P., Kebabian, J.W. & Beart, P.M. N-0434, A very potent and specific new D-2 dopamine receptor agonist. *European journal of pharmacology* **99**, 125-126 (1984).
44. Horn, A.S. et al. Synthesis and radioreceptor binding activity of N-0437, a new, extremely potent and selective D2 dopamine receptor agonist. *Pharm Weekbl Sci* **7**, 208-211 (1985).
45. Seiler, M.P. & Markstein, R. Further characterization of structural requirements for agonists at the striatal dopamine D-1 receptor. Studies with a series of monohydroxyaminotetralins on dopamine-sensitive adenylate cyclase and a comparison with dopamine receptor binding. *Molecular pharmacology* **22**, 281-289 (1982).
46. Seiler, M.P. & Markstein, R. Further characterization of structural requirements for agonists at the striatal dopamine D2 receptor and a comparison with those at the striatal dopamine D1 receptor. Studies with a series of monohydroxyaminotetralins on acetylcholine release from rat striatum. *Molecular pharmacology* **26**, 452-457 (1984).
47. Sumners, C., Dijkstra, D., de Vries, J.B. & Horn, A.S. Neurochemical and behavioural profiles of five dopamine analogues. *Naunyn-Schmiedeberg's archives of pharmacology* **316**, 304-310 (1981).
48. Van der Weide, J., De Vries, J.B., Tepper, P.G. & Horn, A.S. Pharmacological profiles of three new, potent and selective dopamine receptor agonists: N-0434, N-0437 and N-0734. *European journal of pharmacology* **125**, 273-282 (1986).
49. Donthamsetti, P.C. et al. Genetically Targeted Optical Control of an Endogenous G Protein-Coupled Receptor. *Journal of the American Chemical Society* **141**, 11522-11530 (2019).
50. Koehl, A. et al. Structural insights into the activation of metabotropic glutamate receptors. *Nature* **566**, 79-+ (2019).
51. Lau, T.L., Dua, V. & Ulmer, T.S. Structure of the integrin alpha IIb transmembrane segment. *Journal of Biological Chemistry* **283**, 16162-16168 (2008).
52. Causserand, C., Rouaix, S., Akbari, A. & Aimar, P. Improvement of a method for the characterization of ultrafiltration membranes by measurements of tracers retention. *J Membrane Sci* **238**, 177-190 (2004).
53. Riedl, B., Vohl, M.J. & Calve, L. Molecular-Size and Solvation of Low-Molecular Weight Poly(Ethylene Oxide) and Phenol Formaldehyde Resols in Different Solvents. *J Appl Polym Sci* **39**, 341-353 (1990).
54. Fee, C.J. & Van Alstine, J.M. Prediction of the viscosity radius and the size exclusion chromatography behavior of PEGylated proteins. *Bioconjugate Chem* **15**, 1304-1313 (2004).
55. Thomas, D.K. & Charlesby, A. Viscosity relationship in solutions of polyethylene glycols. *Journal of Polymer Science* **42**, 195-202 (1960).
56. Tam, C.M. & Tremblay, A.Y. Membrane pore characterization—comparison between single and multicomponent solute probe techniques. *J Membrane Sci* **57**, 271-287 (1991).

57. Dohmen, M.P.J., Pereira, A.M., Timmer, J.M.K., Benes, N.E. & Keurentjes, J.T.F. Hydrodynamic radii of polyethylene glycols in different solvents determined from viscosity measurements. *J Chem Eng Data* **53**, 63-65 (2008).
58. Geurts, M., Hermans, E. & Maloteaux, J.M. Assessment of striatal D1 and D2 dopamine receptor-G protein coupling by agonist-induced [35S]GTP gamma S binding. *Life sciences* **65**, 1633-1645 (1999).
59. Jiang, L.I. et al. Use of a cAMP BRET sensor to characterize a novel regulation of cAMP by the sphingosine 1-phosphate/G13 pathway. *The Journal of biological chemistry* **282**, 10576-10584 (2007).
60. Luscher, C. & Slesinger, P.A. Emerging roles for G protein-gated inwardly rectifying potassium (GIRK) channels in health and disease. *Nature Reviews Neuroscience* **11**, 301-315 (2010).
61. Donthamsetti, P.C. et al. Optical Control of Dopamine Receptors Using a Photoswitchable Tethered Inverse Agonist. *Journal of the American Chemical Society* **139**, 18522-18535 (2017).
62. Gunaydin, L.A. et al. Natural neural projection dynamics underlying social behavior. *Cell* **157**, 1535-1551 (2014).
63. Sidhu, A. Coupling of D1 and D5 dopamine receptors to multiple G proteins: Implications for understanding the diversity in receptor-G protein coupling. *Mol Neurobiol* **16**, 125-134 (1998).
64. Le Moine, C. & Bloch, B. Expression of the D3 dopamine receptor in peptidergic neurons of the nucleus accumbens: comparison with the D1 and D2 dopamine receptors. *Neuroscience* **73**, 131-143 (1996).
65. Ridray, S. et al. Coexpression of dopamine D-1 and D-3 receptors in islands of Calleja and shell of nucleus accumbens of the rat: opposite and synergistic functional interactions. *European Journal of Neuroscience* **10**, 1676-1686 (1998).
66. Schwartz, J.C. et al. Functional implications of multiple dopamine receptor subtypes: the D1/D3 receptor coexistence. *Brain research. Brain research reviews* **26**, 236-242 (1998).
67. Michino, M. et al. What can crystal structures of aminergic receptors tell us about designing subtype-selective ligands? *Pharmacological reviews* **67**, 198-213 (2015).
68. Ferre, S. et al. Dopamine D1 receptor-mediated facilitation of GABAergic neurotransmission in the rat strioentopenduncular pathway and its modulation by adenosine A1 receptor-mediated mechanisms. *The European journal of neuroscience* **8**, 1545-1553 (1996).
69. Oude Ophuis, R.J., Boender, A.J., van Rozen, A.J. & Adan, R.A. Cannabinoid, melanocortin and opioid receptor expression on DRD1 and DRD2 subpopulations in rat striatum. *Frontiers in neuroanatomy* **8**, 14 (2014).
70. Bernard, V., Normand, E. & Bloch, B. Phenotypical characterization of the rat striatal neurons expressing muscarinic receptor genes. *The Journal of neuroscience : the official journal of the Society for Neuroscience* **12**, 3591-3600 (1992).
71. Banghart, M.R., Neufeld, S.Q., Wong, N.C. & Sabatini, B.L. Enkephalin Disinhibits Mu Opioid Receptor-Rich Striatal Patches via Delta Opioid Receptors. *Neuron* **88**, 1227-1239 (2015).
72. Kerner, J.A., Standaert, D.G., Penney, J.B., Jr., Young, A.B. & Landwehrmeyer, G.B. Expression of group one metabotropic glutamate receptor subunit mRNAs in neurochemically identified neurons in the rat neostriatum, neocortex, and hippocampus. *Brain Res Mol Brain Res* **48**, 259-269 (1997).
73. Misgeld, U. Innervation of the substantia nigra. *Cell and tissue research* **318**, 107-114 (2004).
74. Yizhar, O., Fenno, L.E., Davidson, T.J., Mogri, M. & Deisseroth, K. Optogenetics in neural systems. *Neuron* **71**, 9-34 (2011).

75. Al-Juboori, S.I. et al. Light scattering properties vary across different regions of the adult mouse brain. *PloS one* **8**, e67626 (2013).
76. Aravanis, A.M. et al. An optical neural interface: in vivo control of rodent motor cortex with integrated fiberoptic and optogenetic technology. *Journal of neural engineering* **4**, S143-156 (2007).
77. Owen, S.F., Liu, M.H. & Kreitzer, A.C. Thermal constraints on in vivo optogenetic manipulations. *Nature neuroscience* **22**, 1061-1065 (2019).
