# Supplementary material for: Cell specific photoswitchable agonist for reversible control of endogenous dopamine receptors
Source: Nat Commun. 2021 Aug 6;12:4775. doi: 10.1038/s41467-021-25003-w (PMC8346604; doi:10.1038/s41467-021-25003-w)
Supplement: Supplementary file 2 — nr-reporting-summary [file 41467_2021_25003_MOESM2_ESM.pdf]

nature portfolio

Corresponding author(s): Ehud Isacoff

Last updated by author(s): Jun 29, 2021

Reporting Summary

Nature Portfolio wishes to improve the reproducibility of the work that we publish. This form provides structure for consistency and transparency in reporting. For further information on Nature Portfolio policies, see our Editorial Policies and the Editorial Policy Checklist.

Statistics

For all statistical analyses, confirm that the following items are present in the figure legend, table legend, main text, or Methods section.

n/a	Confirmed	
☐	☒	The exact sample size (*n*) for each experimental group/condition, given as a discrete number and unit of measurement
☐	☒	A statement on whether measurements were taken from distinct samples or whether the same sample was measured repeatedly
☐	☒	The statistical test(s) used AND whether they are one- or two-sided<br>*Only common tests should be described solely by name; describe more complex techniques in the Methods section.*
☐	☒	A description of all covariates tested
☐	☒	A description of any assumptions or corrections, such as tests of normality and adjustment for multiple comparisons
☐	☒	A full description of the statistical parameters including central tendency (e.g. means) or other basic estimates (e.g. regression coefficient) AND variation (e.g. standard deviation) or associated estimates of uncertainty (e.g. confidence intervals)
☐	☒	For null hypothesis testing, the test statistic (e.g. *F*, *t*, *r*) with confidence intervals, effect sizes, degrees of freedom and *P* value noted<br>*Give P values as exact values whenever suitable.*
☒	☐	For Bayesian analysis, information on the choice of priors and Markov chain Monte Carlo settings
☒	☐	For hierarchical and complex designs, identification of the appropriate level for tests and full reporting of outcomes
☒	☐	Estimates of effect sizes (e.g. Cohen's *d*, Pearson's *r*), indicating how they were calculated

Our web collection on statistics for biologists contains articles on many of the points above.

Software and code

Policy information about availability of computer code

Data collection	Data were collected using Clampex 10 (Axon instruments), Viewer3 (Biobserve), BD FACSDiva 6.0 (BD Biosciences), and Zen Black 3.0 (Zeiss).
Data analysis	Data were analyzed using Graph Pad Prism 6.01 (Graph Pad), Clampfit 10.7.0.3. (Axon instruments), Origin SE (Origin Lab), Zen Blue 3.0 (BD Biosciences), ImageJ 1.52b, and custom code written in MATLAB R2020a (MathWorks).

For manuscripts utilizing custom algorithms or software that are central to the research but not yet described in published literature, software must be made available to editors and reviewers. We strongly encourage code deposition in a community repository (e.g. GitHub). See the Nature Portfolio guidelines for submitting code & software for further information.

Data

Policy information about availability of data

All manuscripts must include a data availability statement. This statement should provide the following information, where applicable:

- Accession codes, unique identifiers, or web links for publicly available datasets
- A description of any restrictions on data availability
- For clinical datasets or third party data, please ensure that the statement adheres to our policy

The data that support the findings of this study are available in the Source data file and in the Figshare database as the file MP-D_data.xlsx.

Field-specific reporting

Please select the one below that is the best fit for your research. If you are not sure, read the appropriate sections before making your selection.

[x] Life sciences [ ] Behavioural & social sciences [ ] Ecological, evolutionary & environmental sciences

For a reference copy of the document with all sections, see nature.com/documents/nr-reporting-summary-flat.pdf

Life sciences study design

All studies must disclose on these points even when the disclosure is negative.

Sample size	Sample sizes for were determined based on our previous work or similar work in the literature. n = 3 was the minimum replicate number for the entire study. For in vitro experiment the replicates ranged 3-12 samples. These samples sizes are consistent with our previous work (Donthamsetti et al., JACS, 2017; Donthamsetti et al., JACS, 2019). For behavior experiments, replicates ranged from 4-8 animals. These samples sizes are consistent with our previous work (Lammel, S. et al., Neuron, 2008; Lammel, S. et al., Nature, 2012; Lammel, S. et al., Neuron, 2015).
Data exclusions	There were no exclusions for the flow cytometry and imaging studies. For electrophysiology, cells were excluded if an unstable baseline signal was observed. For behavior, mice were excluded if the viral injection or cannula was not in the correct location.
Replication	All replication attempts for the same condition was performed with transfected cells or animal cohorts on at least two separate days.
Randomization	For flow cytometry and imaging, randomization was not relevant, and experiments were performed with the appropriate controls. For electrophysiology, we randomly selected among cells that expressed a fluorescent protein as a marker of transfection, appeared healthy based on size and shape, and had a stable baseline signal. For animal behavior, animal cohorts were randomly divided to age-matched control groups and treated groups. Groups within a cohort were run in different weeks to ensure that environmental changes within the animal facility testing room did not affect outcomes.
Blinding	The investigators were not blinded in this study. Movement tracking was automatically captured by software rather than manually by an investigator, minimizing the need for blinding. Furthermore, blinding was not feasible due to a lack of funds and staffing.

Reporting for specific materials, systems and methods

We require information from authors about some types of materials, experimental systems and methods used in many studies. Here, indicate whether each material, system or method listed is relevant to your study. If you are not sure if a list item applies to your research, read the appropriate section before selecting a response.

Materials & experimental systems

n/a	Involved in the study	
[ ]	[x]	Antibodies
[ ]	[x]	Eukaryotic cell lines
[x]	[ ]	Palaeontology and archaeology
[ ]	[x]	Animals and other organisms
[x]	[ ]	Human research participants
[x]	[ ]	Clinical data
[x]	[ ]	Dual use research of concern

Methods

n/a	Involved in the study	
[x]	[ ]	ChIP-seq
[ ]	[x]	Flow cytometry
[x]	[ ]	MRI-based neuroimaging

Antibodies

Antibodies used	The following primary antibodies were used: rabbit anti-HA tag (Cell Signaling; C29F4), rat anti-D1R (Sigma-Aldrich; D2944), chicken anti-GFP (Abcam; ab13970). The following secondary antibodies were used: goat anti-rabbit IgG-Alexa Flour 647 (Thermo Fisher Scientific; A-27040), goat anti-rat IgG-Alexa 546 (Thermo Fisher Scientific; A-11081), goat anti-chicken IgG-Alexa Flour 488 (Thermo Fisher Scientific; A-11039).
Validation	For rabbit anti-HA tag (Cell Signaling; C29F4), see: https://www.cellsignal.com/products/primary-antibodies/ha-tag-c29f4-rabbitmab/3724<br>For rat anti-D1R (Sigma-Aldrich; D2944), see: https://www.sigmaaldrich.com/catalog/product/sigma/d2944?lang=en&region=US<br>For chicken anti-GFP (Abcam; ab13970), see: https://www.abcam.com/gfp-antibody-ab13970.html

Eukaryotic cell lines

Policy information about cell lines

Cell line source(s)	HEK293T (ATCC CRL-3216)
Authentication	The cells were authenticated by the University of California, Berkeley Cell Culture Facility using STS analysis and DNA sequencing.
Mycoplasma contamination	HEK293T cells tested negative for mycoplasma by the University of California, Berkeley Cell Culture Facility.
Commonly misidentified lines (See ICLAC register)	No commonly misidentified cell lines were used in the study.

Animals and other organisms

Policy information about studies involving animals; ARRIVE guidelines recommended for reporting animal research

Laboratory animals	The following mouse lines were used for the experiments: D1-Cre (GENSAT, stock number: 017264-UCD, strain code: Tg(Drd1-cre) EY262Gsat/Mmucd) and DAT::IRES-Cre (Jackson Laboratory, stock number: 006660, strain code: B6.SJL-Slc6a3tml.l(cre)Bkmn/J). Males and females were counterbalanced across conditions with no effects of sex observed. The mice were 8-20 weeks old.
Wild animals	The study did not include wild animals.
Field-collected samples	The study did not include samples collected from the field.
Ethics oversight	All procedures complied with the animal care standards set forth by the National Institutes of Health and were approved by University of California Berkeley's Administrative Panel on Laboratory Animal Care.

Note that full information on the approval of the study protocol must also be provided in the manuscript.

Flow Cytometry

Plots

Confirm that:

- [x] The axis labels state the marker and fluorochrome used (e.g. CD4-FITC).
- [ ] The axis scales are clearly visible. Include numbers along axes only for bottom left plot of group (a 'group' is an analysis of identical markers).
- [ ] All plots are contour plots with outliers or pseudocolor plots.
- [ ] A numerical value for number of cells or percentage (with statistics) is provided.

Methodology

Sample preparation	HEK293T cells were labeled with BG-TMR or BG-Alexa647, washed, and then measured by flow cytometry.
Instrument	BD LSR II (BD Biosciences)
Software	FACSDiva 6.0
Cell population abundance	This type of analysis is not relevant to this study because transiently transfected monogenic HEK293T cells were used rather than a heterogeneous mixture of different cell types.
Gating strategy	This type of analysis is not relevant to this study because transiently transfected monogenic HEK293T cells were used rather than a heterogeneous mixture of different cell types.

- [ ] Tick this box to confirm that a figure exemplifying the gating strategy is provided in the Supplementary Information.
